# DNA damage response regulator ATR licenses PINK1-mediated mitophagy

**DOI:** 10.1093/nar/gkaf178

**Published:** 2025-03-19

**Authors:** Christian Marx, Xiaobing Qing, Yamin Gong, Joanna Kirkpatrick, Kanstantsin Siniuk, Galina V Beznoussenko, Gururaj Rao Kidiyoor, Murat Kirtay, Katrin Buder, Philipp Koch, Martin Westermann, Christopher Bruhn, Eric J Brown, Xingzhi Xu, Marco Foiani, Zhao-Qi Wang

**Affiliations:** L eibniz Institute on Aging—Fritz Lipmann Institute (FLI), Beutenbergstr. 11, 07745 Jena, Germany; Center for Pandemic Vaccines and Therapeutics (ZEPAI), Paul Ehrlich Institute (PEI), Paul-Ehrlich-Str. 51-59, 63225 Langen, Germany; L eibniz Institute on Aging—Fritz Lipmann Institute (FLI), Beutenbergstr. 11, 07745 Jena, Germany; L eibniz Institute on Aging—Fritz Lipmann Institute (FLI), Beutenbergstr. 11, 07745 Jena, Germany; Faculty of Basic Medicine, Shenzhen University Medical School, 518055 Shenzhen, China; L eibniz Institute on Aging—Fritz Lipmann Institute (FLI), Beutenbergstr. 11, 07745 Jena, Germany; L eibniz Institute on Aging—Fritz Lipmann Institute (FLI), Beutenbergstr. 11, 07745 Jena, Germany; IFOM, the FIRC Institute of Molecular Oncology, 20139 Milan, Italy; IFOM, the FIRC Institute of Molecular Oncology, 20139 Milan, Italy; L eibniz Institute on Aging—Fritz Lipmann Institute (FLI), Beutenbergstr. 11, 07745 Jena, Germany; L eibniz Institute on Aging—Fritz Lipmann Institute (FLI), Beutenbergstr. 11, 07745 Jena, Germany; L eibniz Institute on Aging—Fritz Lipmann Institute (FLI), Beutenbergstr. 11, 07745 Jena, Germany; Electron Microscopy Center, Jena University Hospital, Ziegelmühlenweg 1, 07743 Jena, Germany; L eibniz Institute on Aging—Fritz Lipmann Institute (FLI), Beutenbergstr. 11, 07745 Jena, Germany; IFOM, the FIRC Institute of Molecular Oncology, 20139 Milan, Italy; Perelman School of Medicine, University of Pennsylvania, Philadelphia 19104, United States; Faculty of Basic Medicine, Shenzhen University Medical School, 518055 Shenzhen, China; IFOM, the FIRC Institute of Molecular Oncology, 20139 Milan, Italy; Department of Oncology, Università degli Studi di Milano, 20122 Milan, Italy; L eibniz Institute on Aging—Fritz Lipmann Institute (FLI), Beutenbergstr. 11, 07745 Jena, Germany; Faculty of Biological Sciences, Friedrich-Schiller-University of Jena, Bachstraße 18k, 07743 Jena, Germany; State Key Laboratory of Microbial Technology, Shandong University, 266237 Qingdao, China

## Abstract

Defective DNA damage response (DDR) and mitochondrial dysfunction are a major etiology of tissue impairment and aging. Mitochondrial autophagy (mitophagy) is a mitochondrial quality control (MQC) mechanism to selectively eliminate dysfunctional mitochondria. ATR (ataxia-telangiectasia and Rad3-related) is a key DDR regulator playing a pivotal role in DNA replication stress response and genomic stability. Paradoxically, the human Seckel syndrome caused by ATR mutations exhibits premature aging and neuropathies, suggesting a role of ATR in nonreplicating tissues. Here, we report a previously unknown yet direct role of ATR at mitochondria. We find that ATR and PINK1 (PTEN-induced kinase 1) dock at the mitochondrial translocase TOM/TIM complex, where ATR interacts directly with and thereby stabilizes PINK1. ATR deletion silences mitophagy initiation thereby altering oxidative phosphorylation functionality resulting in reactive oxygen species overproduction that attack cytosolic macromolecules, in both cells and brain tissues, prior to nuclear DNA. This study discloses ATR as an integrated component of the PINK1-mediated MQC program to ensure mitochondrial fitness. Together with its DDR function, ATR safeguards mitochondrial and genomic integrity under physiological and genotoxic conditions.

## Graphical Abstract

**Graphical Abstract:**
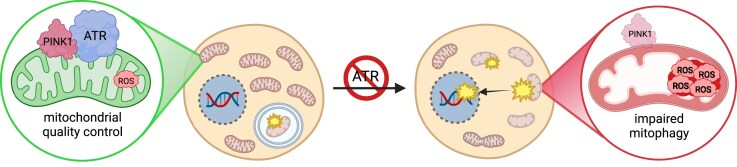

## Introduction

ATR (ataxia-telangiectasia and Rad3-related) is a member of the phosphatidylinositol-3-kinase-related kinase (PIKK) family, which also includes other members such as ATM (ataxia-telangiectasia mutated), DNA-PK (DNA-dependent protein kinase), SMG1 (nonsense-mediated mRNA decay associated PIKK), and mTOR (mechanistic target of rapamycin). In belief of the current knowledge, ATR is activated mainly and directly by DNA lesions comprising single-strand DNA breaks (SSBs) and stalled replication forks. Activated ATR then phosphorylates many downstream substrates, including RAD9, TopBP1, and CHK1, to elicit the ATR-CHK1 axis-mediated DNA damage response (DDR) [1–4]. Thus, ATR conducts a very early step of the nuclear DDR and plays a vital role in safeguarding the genome integrity by controlling specifically the S-phase checkpoint, DNA repair, apoptosis, and transcription [2, 3]. It is also known that the aberrant DDR disrupts mitochondrial function and alters mitochondrial reactive oxygen species (ROS) production, which can further elicit retrogradely nuclear DDR [4–8].

Mitochondria are essential organelles involved in various key cellular processes, including metabolism, energy production, and cell signaling. One of the major functions of mitochondria is to conduct the oxidative phosphorylation (OXPHOS), which, while generates ATP, produces most of the intracellular ROS as a side-product of the electron transport chain (ETC) [4, 5]. ROS are bioactive molecules for cellular signaling, while they can also damage organelles and intracellular macromolecules, including mitochondrial DNA (mtDNA) and nuclear DNA (nDNA) as well as proteins [4, 6, 7]. Hence, ROS homeostasis is vital for cell life; defects in their production and clearance result in ROS accumulation, which is often associated with age-related pathologies and aging [6].

The maintenance of mitochondrial fitness and functionality is vital for cellular integrity and cell fate. Mitochondrial autophagy (mitophagy) conducts a mitochondrial quality control (MQC) program by targeting damaged or dysfunctional mitochondria for degradation, which otherwise produces high levels of ROS, to prevent the spread of oxidative damage to cellular components, including mtDNA and nDNA [8–15]. A prominent mitophagy pathway is mediated by PINK1 (PTEN-induced kinase 1, gene: *PARK6*). In response to mitochondrial injury and disruption of mitochondrial membrane potential (MMP), PINK1 accumulates at the outer membrane of mitochondria to phosphorylate ubiquitin chains and the E3 ligase Parkin (gene: *PARK2*) to initiate mitophagy [9, 10]. This process further recruits other proteins, such as OPTN, LC3B, and p62, needed for forming autophagic vesicles around mitochondria (mitophagosomes) [9, 11–13]. PINK1-mediated mitophagy surveilles and removes dysfunctional or damaged mitochondria, to maintain proper mitochondrial homeostasis [11, 14, 15]. Defects or mutations of mitophagy key genes, such as PINK1 and Parkin, lead to the human neurodegenerative Parkinson’s disease (PD) [16, 17].

Because of its role in handling stalled replication forks in replicative cells, ATR is essential for cell survival [18, 19]. Yet, hypomorphic mutations of the *ATR* gene lead to the autosomal recessive human Seckel syndrome (ATR-SS), characterized by growth retardation, dwarfism, premature aging, neurological symptoms, such as primary microcephaly, seizure, and intellectual disabilities [20–27]. Mouse models carrying hypomorphic mutations of the *Atr* gene imitate human ATR-SS including microcephaly and accelerated aging [21, 22]. The function of ATR in the pathogenesis of the ATR-SS and Seckel mouse models is believed to lie mainly in ATR-mediated DDR to replicative stress [1, 23, 24]. The neurological symptoms of SS patients, such as seizure and intellectual disabilities, may reflect abnormal neuron activities [25, 26–33] and suggest a potential role for ATR in postmitotic neurons. Interestingly, apart from its location in the nucleus, ATR is a HSP90 client in the cytosol [27, 28] and ATR has been implicated in mitochondria-related apoptosis in yeast and cancer models [29, 30]. We recently found that ATR is associated with presynaptic proteins SYT2 and PROT to regulate neuronal vesicle release and firing [31]. All these observations argue for multifaceted yet unknown functions of ATR in pathophysiological processes beyond its role in nuclear DDR. Given the heterogeneity of clinical and experimental phenotypes of the ATR-SS and its relevant models, the etiological mechanism responsible for the developmental defects, tissue impairment (loss of tissue regenerative homeostasis), premature aging, and neuropathies, remains enigmatic.

In this study, we discover that ATR is an integral component of mitochondrial membrane-bound protein complexes under physiological conditions. Via its scaffold, ATR stabilizes and thereby licenses PINK1-mediated mitophagy. ATR loss causes malfunctional OXPHOS resulting in high levels of ROS, which attack cytosol macromolecules and mtDNA prior to the activation of the nuclear DDR. Hence, ATR has a fundamental and physiological function in the MQC program to maintain mitochondrial fitness and prevent the abnormal ROS release from sick mitochondria, which otherwise damage cellular components, including genomic DNA.

## Materials and methods

### Mice

Mice carrying the conditional (floxed) *Atr* allele [1, 23] were crossed with CreER^T2^ (CER) in order to generate mice with tamoxifen-inducible deletion of ATR [ATR inducible knockout (ATR-iKO)]; with CamKII-Cre [32] to delete ATR in pyramidal neurons of the forebrain (designated as ATR-FBΔ) starting after birth. All animal experiments and breeding were conducted according to the German animal welfare legislation and approved by the Thüringer Landesverwaltungsamt (animal license nr.: 03-042/16). The ATR and Cre genotypes of mice were determined by polymerase chain reaction (PCR) on DNA extracted from tail biopsies as previously described [31, 33].

### Cell culture

Primary mouse embryonic fibroblasts (pMEFs) were isolated as previously described in [33] from embryos in developmental stage E13.5 of the ATR-CER colony. pMEFs were maintained in a humidified incubator at 37°C and 3% oxygen with 5% CO_2_ in Dulbecco’s modified Eagle’s medium (DMEM; 4.5 g/l glucose; Thermo Fisher Scientific) supplemented with 10% fetal bovine serum (FBS), 2 mM l-glutamine, 1 mM sodium pyruvate, and 100 U/ml penicillin and 100 U/ml streptomycin (Pen/Strep) (all from Thermo Fisher). Mouse neuroblastoma cell line N2A and human HeLa, HEK293T, U2OS, and HCT116 cells were maintained in a humidified incubator at 37°C and 20% oxygen with 5% CO_2_ in DMEM supplemented with 10% FCS, 1 mM sodium pyruvate, and Pen/Strep. Cells were routinely passaged at 90% confluency. HCT116 cells were a gift from Dr B. Vogelstein (Baltimore, MD, USA).

### Construction of vectors

For imaging proximity ligation assay (PLA) and biochemical experiments, human ATP5A, TIM23, and TOM40 complementary DNAs were sub-cloned into the HA vector (pcDNA3.0-HA-VEC; NOVO Bio). PINK1-GFP truncations (PINK1-D1 to D3) were generated using PINK1-GFP plasmid (#13316, pcDNA-DEST47 PINK1 C-GFP, Addgene). GFP-PINK1-FL (#13316, pcDNA-DEST47 PINK1 C-GFP), HSP90α-mCherry (#108222, pCherry.90 alpha), FLAG-ATR-WT (#31611, pcDNA3-ATR-WT), FLAG-ATR-KD (#31612, pcDNA3-ATR kinase dead), FLAG-ATR-N (#53767, pcDNA4-ATR-N), FLAG-ATR-M (#53768, pcDNA4-ATR-M), and FLAG-ATR-C (#53769, pcDNA4-ATR-C) plasmids were purchased from Addgene.

Primers used for construction of expression vectors are listed below:

**Table:** 

PINK1-D1-forward:	TTCGGTACCATGGTGGTGCGGGCCTGGGG
PINK1-D1-reverse:	CACCACCATGGTACCGAATTCCTTCAAGC
PINK1-D2-forward:	CTAAGCCTCAAGCTCGAGTGCGGCCGCAA
PINK1-D2-reverse:	CTCGAGCTTGAGGCTTAGATGAAGCACAT
PINK1-D3-forward:	TTGCAGGGCTCACGCCTCCACCCTGAAGG
PINK1-D3-reverse:	GAGGCGTGAGCCCTGCAAGCGTCTCGTGT
HA-ATP5A-forward:	GACGTTCCAGATTACGCTGAATTCATGC TGTCCGTGCGCGTTGC
HA-ATP5A-reverse:	GTGTGATGGATATCTGCAGAATTCTTAA GCTTCAAATCCAGCCAA
HA-TIM23-forward:	GACGTTCCAGATTACGCTGAATTCATGG AAGGAGGCGGGGGAA
HA-TIM23-reverse:	TGTGATGGATATCTGCAGAATTCTCAGA GTGACTGTTGGAGCAA
HA-TOM40-forward:	GACGTTCCAGATTACGCTGAATTCATGG GGAACGTGTTGGCTGC
HA-TOM40-reverse:	TGTGATGGATATCTGCAGAATTCTCAGC CGATGGTGAGGCCAAA

### Cell treatment and transfection

ATR^f/f^-CER and ATR^f/+^-CER MEFs were treated for 3 days with 1 μM 4-hydroxytamoxifen (4-OHT, in ethanol; Sigma–Aldrich) or ethanol (mock) to induce the deletion of ATR or as control, respectively. Afterwards, cells were re-plated into suitable experimental formats and were analyzed at the indicated days post 4-OHT (dpo).

We used Lipofectamine 2000 (11668030; Thermo Fisher) for HeLa and HCT116 cell transfection and Lipofectamine 3000 (L3000001; Thermo Fisher) for transfection of HEK293T and U2OS cells, using the protocols recommended by the manufacturer. ATR small hairpin RNA (shRNA) and control (pLKO1) plasmids were a gift from Dr O.F. Capetillo (CNIO, Spain).

We used Lipofectamine RNAiMAX (13778075; Thermo Fisher) to transfect small interferring RNAs (siRNAs) into HCT116 cells, using the protocol recommended by the manufacturer. Scrambled siRNAs (siCtl: equal mixture of sc-37007, sc-44230, and sc-44231) and targeted siRNAs against ATR (siATR#1: sc-29763), PINK1 (siPINK1#1: sc-44598), TIM23 (sc-44155), TOM7 (sc-89537), and HSP90 (sc-35608) were purchased from Santa Cruz Biotechnologies, and (siATR#2: EHU040341-50UG) (siATR mix: equal mixture of siATR#1 and #2) and (siPINK1#2: EHU057101-5OUG) (siPINK1 mix: equal mixture of siPINK1#1 and #2) from Sigma–Aldrich.

For assays, cells were treated with 200 μM of *t*-butyl hydrogenperoxide (tBHP; 458139), 450 ng/ml of ethidium bromide (EtBr; E1385), 100–500 μM of hydroxyurea (HU; H8627; all from Sigma–Aldrich), 2 μM of carbonylcyanide 4-(trifluoromethoxy)-phenylhydrazone (FCCP; ab120081), 10 μM of MitoParaquat (MitoPQ; ab146819), 10 μM of MitoTempol (MitoT; ab144644; all from Abcam), 10 μM of MG-132 (S2619), 5 μM Mdivi-1 (S7162), or 2 μM of VE821 (S8007; all from Selleck Chemicals) for up to 48 h. Dimethyl sulfoxide or phosphate-buffered saline (PBS) was used as control treatment for all mentioned experiments.

### TEM of shRNA-transfected HeLa cells

The electron microscopic analysis was performed as previously described in [34, 35].

### Immune electron microscopy (EM) gold-labeling of HeLa cells

Electron microscopic examination and immune EM gold-labeling based on pre-embedding were performed as previously described in [35, 36]. The number of ATR-tagged gold particles in different compartments of the cell was counted manually and percentages were calculated. The labeling density of ATR on different cellular structures was assessed and calculated as described in [37]. For this, we used the following criteria: gold particles were considered to label the nuclear envelope or the endoplasmic reticulum (ER), when these particles were observed over lumens or membranes of these compartments; gold particles were considered as a label over mitochondrial membrane when these particles were observed over the plasma membrane.

### TEM of pMEFs and human HCT116 cells

The electron microscopic analysis was performed as previously described in [28, 38, 39].

### Flow cytometry analysis

Analysis of mitochondrial functions: for all following single flow cytometry analyses, cells were incubated with the corresponding dye for 30 min at 37°C. Mitochondrial ROS: 1 μM Mitosox (M36008); mitochondrial mass: 100 nM MitoTracker Deep Red FM (M22426); and MMP: 50 nM 3,3′-dihexyloxacarbocyanine iodide (DiOC_6_(3); D273; all from Thermo Fisher). Afterwards, cells were harvested using StemPro Accutase (A1110501; Thermo Fisher) and 10 000 cells per sample were analyzed on a FACS Canto II (BD Bioscience); data were gated to exclude debris. Apoptosis assays were performed as described in [40]. Cellular autophagy levels were measured with the autophagy detection kit (ab139484; Abcam) following the manufacturer’s instruction. Total 10 000 cells per sample were analyzed on a FACS Canto II; data were gated to exclude debris.

### β-Galactosidase staining

Assays were performed as described in [39].

### Fluorescence microscopy

Before fixation, cells were incubated with 100 nM MitoTracker Deep Red FM at 37°C for 30 min. Cells were fixed with 4% paraformaldehyde (PFA; in PBS), washed with PBS and incubated for 1 h at room temperature (RT) in blocking solution (1% bovine serum albumin (BSA), 0.4% Triton X-100 in PBS). Following, samples were incubated overnight at 4°C in primary antibody solutions against PINK1 (1:250; sc517353; Santa Cruz), LC3B (1:250; 2775S; Cell Signaling), p62 (1:500 in BS1; PM045; MBL), or ATR (1:250; ab2905; Abcam). On the next day, cells were washed three times in PBS and incubated for 2 h at RT in Alexa Fluor 555-conjugated secondary anti-mouse (1:500; ab150114) or anti-rabbit (1:500; ab150078) or Alexa Fluor 488-conjugated secondary anti-rabbit (1:500; ab150077; all from Abcam) antibody solutions. Afterwards, cells were washed once with PBS, incubated for 15 min with 1 μg/ml DAPI and washed again with PBS before mounting with ProLong Gold Antifade Mountant (P10144; Thermo Fisher). Images were examined using a Zeiss AxioImager ApoTome microscope (structured illumination) (Carl Zeiss). Zen software (Carl Zeiss) was used to analyze the pictures.

### Generation of inducible shATR stable cell lines

The shRNA constructs were annealed and ligated into the pLKO-Tet-On vector (Addgene, #21915). Sequences for shATR-1 and shATR-2 are from the sequence as reported [41]. The shRNA was introduced into U2OS cells using lentiviral infection followed by selection with puromycin (1.0 μg/ml) to generate stable shATR-expressing cell lines.

### PLA

The assays were performed in U2OS cells following the manufacturer’s instruction using a Duolink^TM^ In Situ Red Starter Kit Mouse/Rabbit (DU092101-1KT; Sigma–Aldrich). Cells were incubated overnight at 4°C with antibodies against ATR (1:1000; ab2905), GFP (1:5000; sc-9996), TIM23 (1:500; sc-514463), PINK1 (1:500; ab232374), TOM40 (1:500; sc-365467), total OXPHOS rodent WB antibody cocktail (1:2500; ab110413), or FLAG (1:800; ab110413). Images were examined using a Zeiss AxioImager ApoTome microscope (structured illumination) (Carl Zeiss). Zen software (Carl Zeiss) was used to analyze the pictures. For image analysis, 30 cells from three independent experiments were randomly chosen to quantify the number of PLA foci manually.

### 1D blue native polyacrylamide gel electrophoresis

The method refers to recent publications [42, 43]. Mitochondria were isolated from N2A cells and lysated in sample buffer [75 mM Bis–Tris (Sigma, B9754) (pH 7.0), 750 mM 6-aminocaproic acid (Sigma, A2504), 10% Glycerol, 1% Triton X-100]. The lysate was divided into two aliquots, one of which was used for immunoprecipitation (IP) by rabbit anti-ATR antibody (1:1000; ab2905; Abcam). The IP samples were incubated at 99°C for 10 min to disrupt mitochondrial complexes. To negatively charge proteins without destroying structure, 1‰ Coomassie blue G-250 was added to mitochondrial lysates and IP samples. Pre-cast native 4–15% Mini-PROTEAN^®^ TGX™ Precast Protein Gels (Bio-Rad, #4561086) were used for blue native polyacrylamide gel electrophoresis (BN–PAGE). After electrophoresis, the gel was incubated in 1× sodium dodecyl sulfate (SDS) running buffer for 15 min and was transferred onto a 0.45-μm PVDF membrane. Mouse antibodies against ATR (1:250; sc515173; Santa Cruz), TOM22 (1:1000; ab134274; Abcam), ATP5A (1:2500; ab14748; Abcam), and PINK1 (1:100; sc-517353; Santa Cruz), were used to detect the mobility shift of ATR-probe mitochondrial complex.

### Autophagy assay (dual-fluorescence reporter assay)

For mitophagy analysis, we constructed stable cells by transfecting the mCherry-EGFP-LC3B plasmid (Addgene, 110060) into U2OS cells. After selection by puromycin (2 μg/ml), stable single cell lines were established and verified for expression of GFP and RFP. For autophagic examination, siRNAs against ATR were transfected with or without ATR expressing vectors via Lipofectamine 3000 into the constructed cell lines. Cells expressing mCherry-EGFP-LC3B are subject to 1 μM FCCP for 20 h followed by imaging cells of alive under a Zeiss Apotome microscope with a 63× objective. To analyze the maturation of phagosomes, the 488- and 561-nm laser lines were used to excite mCherry-GFP. The number of mCherry single-positive and mCherry/GFP double-positive puncta from >90 cells of each condition and three independent single cell lines was scored under ImageJ.

### Analysis of poly(ADP-ribose) formation

Cells were fixed with 4% PFA, washed trice with PBS, and incubated for 2 h at RT in blocking solution (5% normal donkey serum, 1% BSA, 0.4% Triton X-100 in PBS; filtered). Afterwards, samples were incubated overnight at 4°C in anti-PAR antibody (1:400; 51-8114KC; BD Pharmingen). On following day, cells were washed trice in PBS and incubated for 1 h at RT in Alexa Fluor 555-conjugated secondary anti-rabbit antibody (1:400; Abcam) and 1 μg/ml DAPI. Coverslips were then washed four times with PBS and mounted using ProLong Gold Antifade Mountant (Thermo Fisher). Images were examined using a Zeiss AxioImager ApoTome microscope (structured illumination) (Carl Zeiss). Zen software (Carl Zeiss) was used to analyze the pictures. For image analysis, 200 cells from three independent experiments were randomly chosen to quantify the number of poly(ADP-ribose) (PAR) foci manually.

### Cell Mito Stress Test and Seahorse analysis

The mitochondrial respiration was performed using the Cell Mito Stress Test as described previously [44] using a Seahorse XFe96 Analyzer (Agilent Technologies).

### Seahorse analysis of brain biopsies

Whole brains of 3-month-old ATR-FBΔ mice were isolated and hippocampus tissues excavated. Tissue biopsies (∼1–2 mm in diameter) were directly put into single wells of Seahorse XF24 Islet Capture Microplates (101122-100, Agilent Technologies) previously filled with 0.5 ml pre-warmed Seahorse XF Base Medium (supplemented with 10 mM d-glucose, 2 mM l-glutamine, and 1 mM sodium pyruvate). Brain biopsies were covered with small nets included with the Islet Capture Microplates and incubated for 30 min in a CO_2_-free incubator at 37°C. A Seahorse XFe24 Analyzer (Agilent Technologies) was used to measure basal oxygen consumption rate (OCR) and extracellular acidification rate (ECAR) as pmoles/min and mpH/min, respectively, in four cycles of 3 min mix, 3 min hold, and 3 min measure periods at 37°C. At the end of the measurement, protein concentration per well was quantified using the Pierce BCA Protein Assay Kit (23225, Thermo Fisher) following the manufacturer’s instructions and the observed OCR and ECAR were normalized to corresponding protein concentrations. Wave software was used to analyze the datasets.

### Preparation of protein lysates

After harvesting by scraping, cells were re-suspended in RIPA buffer [50 mM Tris–HCl (pH 8.0), 150 mM NaCl, 1 mM ethylenediaminetetraacetic acid, 1% Triton X-100, 1% sodium deoxycholate, 0.1% SDS] supplemented with PhosphoStop and protease inhibitor cocktail (PIC; both from Roche), followed by brief sonication in a Bioruptor (Diagenode) (five cycles with 30 s on and 30 s off with high intensity at 4°C). Protein quantification was done using the Pierce BCA Protein Assay Kit following the manufacturer’s instruction.

### Cell fractionation and Accutase treatment

Mitochondrial fractions were isolated as described in [39]. Half of the mitochondrial pellet was directly re-suspended in RIPA buffer and the other half was incubated at RT for 15 min in pre-warmed StemPro Accutase. Accutase reaction was stopped by adding the four-times volume of RIPA buffer. All samples were briefly sonicated. Final volumes of mitochondrial fractions ± Accutase treatment were identical.

### Co-immunoprecipitations

After harvesting by scraping, cells were re-suspended in NETN buffer [50 mM Tris–HCl (pH 8.0), 150 mM NaCl, 1% NP-40] supplemented with PhosphoStop and PIC, followed by brief sonication. Inputs were taken and 10 μl Dynabeads (10006D, A and 10007D, G; Thermo Fisher; 1:1 mixture) pre-incubated with 1 μg of primary antibodies against Rabbit IgG (ab172730), Mouse IgG1 (ab18447), PINK1 (ab232374), HSP90 (ab13492), ATP5A (ab14748; all from Abcam), FLAG (F-1804, Sigma–Aldrich), or ATR (sc-515173; all from Santa Cruz) were added to the lysates. IPs were performed for 3 h at 4°C with rotation. Afterwards, beads were washed three times in NETN buffer. Proteins were separated from the beads by heating at 95°C for 10 min in 2× SDS-sample buffer [12% β-mercaptoethanol, 120 mM Tris–HCl (pH 6.8), 10% glycerol, 3% SDS, 0.08% bromophenol blue].

### Immunoblotting

Sample preparation was done as described in [40, 45]. Antibodies used were as follows: BNIP3L/Nix (1:2500; 12396S), phospho-S65 ubiquitin (1:2500; 62802S), PINK1 (1:1000; 6946S, for use in human samples), CHK1 (1:1000; 2345S), phospho-S317 CHK1 (1:1000; 12302P; above all from Cell Signaling); ATR (1:250; sc515173; for murine samples), GFP (1:5000; sc-9996), TIM23 (1:2500; sc-514463), PINK1 (1:1000; sc517353; Santa Cruz, for use in murine samples), TOM22 (1:1000; ab134274; Abcam), TOM40 (1:1000; sc-365467) (above all from Santa Cruz); ATR (1:1000; 13934S; Santa Cruz, for human samples), LC3B (1:1000; 2775S), BIM (1:2500; 2933S), ATP5A (1:2500; ab14748; Abcam), HSP90 (1:5000; ab13492), ubiquitin (1:5000; ab19247), COX IV (1:5000; ab16056), VDAC1 (1:2500; ab15895), total OXPHOS rodent WB antibody cocktail (1:2500; ab110413) (above all from Abcam); p62 (1:2500; PM045; MBL); TOM7 (1:1000; PA5-110508; Thermo Fisher), FLAG (1:5000; F-1804; Sigma–Aldrich), HA (1:1000; A190-208A; Bethly), and phospho-S139 H2AX (γH2AX; 1:2500; 07164; Merck Millipore). Protein loading was controlled by antibodies against vinculin (1:5000; sc25336; Santa Cruz), β-actin (1:10000; A5441), and α-tubulin (1:10000; T5168; both from Sigma–Aldrich). Peroxidase-conjugated anti-mouse (1:5000; 5220-0341) anti-rabbit (1:5000; 5220-0336; both from KPL Sera Care) secondary antibodies were used for detection of specific signals with Pierce ECL Western Blotting Substrate (32106; Thermo Fisher) and WesternBright Sirius HRP substrate (K-12043-D10; Advansta) on an Amersham Imager 600 (GE Healthcare Lifesciences). For IP samples, peroxidase-conjugated VeriBlot for IP Detection Reagent (HRP) (1:2500; ab131366) and anti-mouse IgG for IP (HRP) (1:2500; ab131368; both from Abcam) secondary antibodies were used. Protein expression levels were quantified using ImageJ.

### Detection of carbonylated proteins by western blotting

Carbonylated proteins in cell and brain lysates were labeled with the Oxidized Protein Western Blot Detection Kit (ab178020; Abcam) following the manufacturer’s instruction.

### mtDNA isolation and mtDNA damage assay

The mtDNA was isolated from mitochondrial fractions as described for genomic DNA [33]. To detect mtDNA breaks, we used mouse mtDNA-specific primers (forward: 5′-CCCAGCTACTACCATCATTCAAGT-3′, reverse: 5′-GAGAGATTTTATGGGTGTATTGCGG-3′) to amplify a 16-kb fragment of mtDNA, and (forward: 5′-CCCAGCTACTACCATCATTCAAGT-3′, reverse: 5′-GATGGTTTGGGAGATTGGTTGATG-3′) to amplify a short 117-bp mtDNA amplicon for normalization as described in [46]. mtDNA breaks were determined by the grey scale ratio intensity of 16 kb over the 117-bp PCR products as described previously in [47].

### RNA sequencing

Total RNA was extracted using RNeasy Mini Kit (Qiagen). Sequencing of RNA samples was done using Illumina’s next-generation sequencing methodology [48]. In detail, quality check and quantification of total RNA was done using the Agilent Bioanalyzer 2100 in combination with the RNA 6000 Nano Kit (Agilent Technologies). Library preparation was done using TruSeq Stranded Total RNA RiboZero Gold Kit (Illumina) following the manufacturer’s description. Quantification and quality check of libraries was done using the Agilent Bioanalyzer 2100 in combination with the DNA 7500 kit. Libraries were sequenced on a HiSeq2500 running in 51 cycle/single-end/high-output mode using sequencing by synthesis (SBS) sequencing chemistry v3. All libraries were pooled and sequenced in four lanes. Sequence information was extracted in FastQ format using Illumina’s bcl2fastq v1.8.4. Sequencing resulted in around 39mio reads per sample.

### Detection of differentially expressed genes

The RNA sequencing (RNA-seq) reads were mapped with STAR (v2.5.4b, parameters: –alignIntronMax 100 000 –outSJfilterReads –outSAMmultNmax 1 Unique –outFilterMismatchNoverLmax 0.04) [49] to the *Mus musculus* genome (GRCm38) with the Ensembl genome annotation (Release 91). For each Ensembl gene, reads that map uniquely to one genomic position were counted with FeatureCounts (v1.5.0, multimapping or multi-overlapping reads were not counted, stranded mode was set to “–s 2”, Ensembl release 91 gene annotation) [50].

The raw counts per gene per sample were analyzed with R (v3.5.0) using the package DESeq2 (v1.20.0) [51] for differential expression. ATR-iKO MEF samples were compared with control samples while the ATR-iKO group being the reference level. For each gene of the comparison, the *P*-value was calculated using the Wald significance test. Resulting *P*-values were adjusted for multiple testing with Benjamini & Hochberg correction. The log_2_ fold changes (L2FC) were shrunk with lfcShrink to control for variance of L2FC estimates for genes with low read counts.

### Protein mass spectrometry (proteomics)

#### Sample preparation

After harvesting by scraping, cell pellets were lyzed by addition of lysis buffer [4% SDS, 100 mM HEPES (pH 8.5), 50 mM DTT] followed by sonication in a Bioruptor (Diagenode) (10 cycles, 1 min on/30 s off, 20°C). Samples were heated (95°C, 10 min) and sonication steps repeated. The lysates were clarified by brief centrifugation, incubated with iodacetamide 15 mM) at RT in the dark. Each sample was treated with 4 volumes ice cold acetone to precipitate the proteins (overnight, −20°C). The samples were centrifuged at 20800 × *g* (30 min, 4°C). Supernatant was removed and the pellets washed twice with 400 μl of ice cold 80% acetone (20% water). Pellets were air-dried before dissolving in digestion buffer (3 M urea in 0.1 M HEPES, pH 8.0) at 1 μg/μl. A 1:100 w/w amount of LysC (Wako sequencing grade) was added to each sample before incubation (4 h, 37°C, 1000 rpm). Samples were diluted 1:1 with milliQ water and incubated with a 1:100 w/w amount of trypsin (Promega sequencing grade) (overnight, 37°C, 650 rpm). Digests were acidified with 10% trifluoroacetic acid and desalted with Waters Oasis HLB μElution Plate 30 μm in the presence of a slow vacuum according to manufacturer’s instructions. The eluates were dried down with the speed vacuum centrifuge and dissolved in 5% acetonitrile, 95% milliQ water, 0.1% formic acid at a concentration of 1 μg/μl. A total of 10 μl was transferred to a mass spectrometry (MS) vial and 0.25 μl of HRM kit peptides (Biognosys) were spiked into each sample prior to analysis by liquid chromatography–tandem mass spectrometry (LC–MS/MS).

#### LC–MS/MS

Peptides were separated using the nanoAcquity UPLC system (Waters) fitted with a trapping (nanoAcquity Symmetry C18, 5 μm, 180 μm × 20 mm) and an analytical column (nanoAcquity BEH C18, 1.7 μm, 75 μm × 250 mm). The outlet of the analytical column was coupled directly to Orbitrap Fusion Lumos (Thermo Scientific) using the Proxeon nanospray source. The samples (∼1 μg for Data-dependent acquisition (DDA) and ∼3 μg for Data-independent acquisition (DIA)) were loaded with a constant flow of solvent A (0.1% formic acid) (5 μl/min) onto the trapping column for 6 min. Peptides were eluted via the analytical column with a constant flow of 0.3 μl/min. During the elution step, the percentage of solvent B (acetonitrile, 0.1% formic acid) increased in a nonlinear fashion from 0% to 40% in 90 min. Total runtime was 115 min. Samples were introduced to the MS via a Pico-Tip Emitter 360 μm OD × 20 μm ID; 10 μm tip (New Objective) with a spray voltage of 2.2 kV. Capillary temperature was 300°C. The RF ion funnel was 30%. Data from pools of each condition/each sample were first acquired in DDA mode to contribute to a sample specific spectral library as follows: full scan MS (350–1500 *m/z*) was acquired in profile mode in the Orbitrap with resolution of 60K. Fill time was set to 50 ms with AGC of 2 × 10^5^ ions. “Top Speed” method was employed with an intensity threshold of 5 × 10^4^ for fragmentation (HCD, 30%) and quadrupole isolation (1.4 Da) and MS2 measurement in the Orbitrap (resolution 15K, fixed first mass 120 *m/z*), with a cycle time of 3 s. Monoisotopic Precursor Selection (MIPS) algorithm was employed. MS/MS data were acquired in centroid mode. Only multiply charged (2+–7+) precursor ions were selected, and dynamic exclusion was employed (15 s, relative mass window 10 ppm). Isotopes were excluded. For data acquisition and processing of the raw data Xcalibur 4.0 (Thermo Scientific) and Tune v2.1 were employed.

For the DIA data acquisition, the same gradient conditions were applied to the LC as for the DDA and the MS conditions were varied as described: full scan MS were acquired in the Orbitrap with resolution of 120K. The default charge state was set to 4+. The fill time was set at maximum of 20 ms with limitation of 5 × 10^5^ ions. DIA scans were acquired with 34 mass window segments of differing widths across the MS1 mass range. MS/MS spectra were acquired in the Orbitrap with a resolution of 30K over the mass range 200–2000 *m/z* after accumulation of 1 × 10^6^ ions or after filling time of 70 ms (whichever occurred first). Ions were injected for all available parallelizable time. Data were acquired in profile mode.

#### Data analysis

For library creation, the DDA data (pools, individual samples or high pH data) were searched using MaxQuant (v1.5.3.28). The data were searched against a species specific (*M. musculus*) Uniprot database with a list of common contaminants appended, as well as the HRM peptide sequences. The data were searched with the following modifications: Carbamidomethyl (C) (Fixed) and Oxidation (M) / Acetyl (Protein N-term) (Variable). The mass error tolerance for the full scan MS and MS/MS spectra was set at 20 ppm. A maximum of 1 missed cleavage was allowed. The identifications were filtered to satisfy the false discovery rates (FDR) of 1% on peptide and protein level. A spectral library was created from the MaxQuant output of the DDA runs using Spectronaut (v10, Biognosys AG). This library contained 42 240 precursors, corresponding to 4180 protein groups using Spectronaut protein inference. DIA data were then uploaded and searched against this spectral library in Spectronaut. Relative quantification was performed in the software for each pairwise comparison using the replicates from each condition. The candidate table and data reports were then exported to excel and further data analysis and visualization were performed with R-studio (v0.99.902) using in-house pipelines and scripts.

The datasets were post-analyzed with a cutoff of *q* < 0.05. Gene ontology analysis was done with Gorilla [52] and REVIGO [53] web tools, Cytoscape [54], and ingenuity pathway analysis (IPA) software with a cutoff of *q* < 0.05.

### Statistical analysis

Statistical analyses were done using two-tailed unpaired (western blot quantifications) or paired (all other quantifications) parametric *t*-test with GraphPad Prism 8 (GraphPad Software). *P*-values are indicated within individual graphs with *P* < .05 or lower being statistically significant.

## Results

### ATR locates at mitochondria and maintains mitochondrial structures

**Figure 1.**
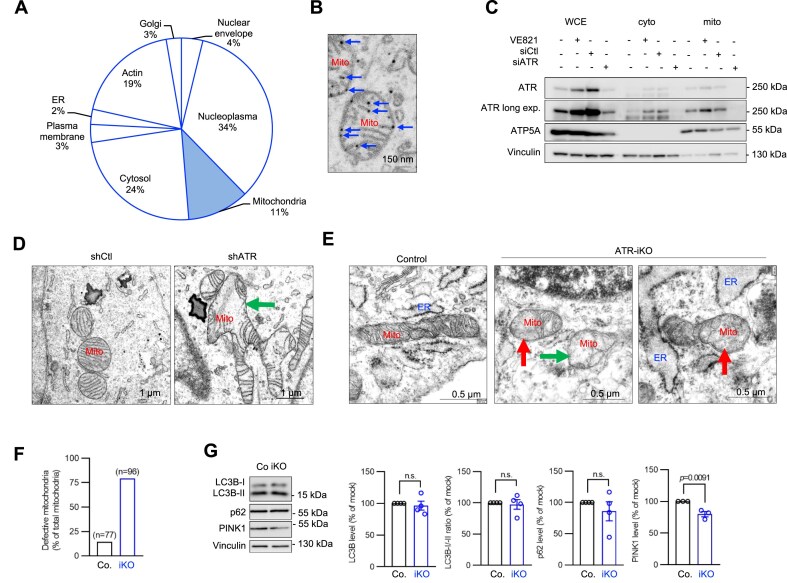
ATR anchors at mitochondria and its deletion causes mitochondrial malformations. (**A**) Human HeLa cells were analyzed by TEM. Quantification of ATR-immunogold labeling from TEM analysis and graphical presentation of ATR’s intracellular distribution. (**B**) A representative TEM image of mitochondria. ATR was labeled using nano-gold particle-conjugated anti-ATR antibody. Blue arrows point gold particles within mitochondria (Mito). (**C**) Western blot analysis of the indicated proteins in WCEs, cytosolic (cyto), and mitochondrial (mito) cellular fractionations of human HCT116 cells. ATR knockdown experiments was conducted using RNA interference (RNAi) targeted against ATR (siATR). The crambled siRNA (siCtl) is a control. VE821 is an ATR inhibitor. Vinculin and ATP5A are controls for protein loading in the corresponding fractionation. *n* = 3. (**D**) Mitochondrial morphologies viewed by TEM in ATR-KD HeLa cells. Green arrow points swollen mitochondria with *cristae* defects. (**E**) ATR-iKO in pMEFs was induced by 4-OHT treatment (see Supplementary Fig. S2A and B). Mitochondrial morphologies are analyzed by TEM at 5 dpo. Green arrows mark mitochondria lacking “cristae” structures and red arrows point to mitochondria with a fused appearance of intact and malformed “cristae”. (**F**) Quantification of malformed mitochondria in ATR-iKO pMEFs. The number (*n*) of mitochondria counted. (**G**) Western blot analysis of the indicated proteins in ATR-iKO pMEFs and quantified using ImageJ, which are shown on right. Vinculin was used to control protein loading. *n* = 3. Error bars in all subfigures show standard error of the mean (SEM). The statistical analysis was performed using two-tailed *t*-tests. *P*-values are indicated within individual graphs. n.s.: not significant.

The function of ATR has been so far mainly associated with nuclear DDR [1, 23]. Yet, it is implicated in other cytoplasmic activities [27, 28, 31]. These scarce observations hence raise a possibility that ATR has functions beyond its role in nuclear DDR. To explore ATR’s novel functions, we first performed transmission electron microscopy (TEM) to visualize nano-gold particle-stained ATR in human HeLa cells. Apart from its well-documented location in the nucleus, ATR was also found in the cytosol, membranous compartments, and other organelles, with about 11% of total ATR particles at mitochondria, notably associated with outer and inner membranes (Fig. 1A and B, blue arrows). Naturally, the subcellular distribution frequency of ATR may vary depending on cell types and organisms. The specificity of ATR labeling was controlled by ATR knockdown (ATR-KD) via shRNA, which greatly reduced gold-labeled ATR (Supplementary Fig. S1A and B). In addition, cell fractionation experiments corroborated the presence of ATR in cytosol as well as in the mitochondrial fraction of human HCT116 cells, which was abrogated after siRNA-mediated ATR-KD in both the cytosolic and mitochondrial compartment, but not by the ATR kinase inhibitor VE821 (Fig. 1C and Supplementary Fig. S1C).

Interestingly, TEM analysis revealed that ATR deletion resulted in prominently enlarged, abnormal mitochondria with deformed “cristae” in HeLa cells (Fig. 1D). Next, we extended our analysis to pMEFs carrying the floxed *Atr*^f/f^ allele and the CreER^T2^ transgene. Addition of 4-OHT enables an inducible deletion of ATR in these cells (designated as ATR-iKO) and provides a good opportunity for time course experiments, which is critical for this study (see below). We found an efficient deletion of ATR readily at 5 dpo (Supplementary Fig. S2A and B) and thus carried out most of mitochondrial experiments at 5 dpo, unless specified. Similar to those in ATR-KD HeLa cells, ATR-iKO mitochondria were deformed, characterized by either being devoid of “cristae” (Fig. 1E, green arrow) or having a partially normal “cristae” structure, indicative of abnormal mitochondrial homeostasis (Fig. 1E, red arrows). About 75% of mitochondria in ATR-iKO cells had aberrant morphologies, in sharp contrast to only 14% in controls (Fig. 1F). Moreover, fluorescence microscopy of mitochondrial networks revealed a mild, yet significantly, increase of interconnectivity (the area per perimeter ratio) (Supplementary Fig. S2C and D). The elevated interconnectivity suggests that defective mitochondria tend to fuse in order to cope with (or compensate for) dysfunctional mitochondria in ATR mutant cells, which correlated with an increased mitochondrial mass (Supplementary Fig. S2E). Finally, ATR-KD in HCT116 cells also resulted in severely distorted mitochondria (green arrows), which phenocopied the mitochondrial morphology in cells after knocking down the mitophagy regulator PINK1 (Supplementary Fig. S2F and G). These observations suggest that ATR plays a role in mitochondrial homeostasis likely by affecting the MQC, which is responsible for removing damaged mitochondria [11, 14].

Consistent with this hypothesis, we found that despite malformed mitochondria, ATR deficiency did not change autophagy profile as analyzed by flow cytometry (Supplementary Fig. S3A). Immunofluorescence microscopy revealed no obvious co-localization of LC3B and p62 within the mitochondrial networks in ATR mutant cells (Supplementary Fig. S3B and C). Furthermore, western blot analysis detected a similar amount of autophagosome proteins LC3B and p62, as well as the LC3B-I/LC3B-II ratio, between ATR-iKO and control pMEFs (Fig. 1G). All these suggest that ATR deficiency silences mitophagy.

### ATR interacts with and stabilizes PINK1 at mitochondria

**Figure 2.**
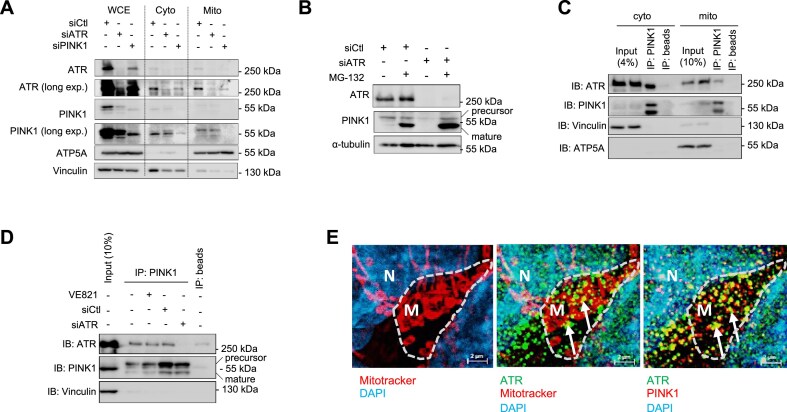
ATR associates with PINK1 at mitochondria. (**A**) Western blot analysis of the indicated proteins in WCEs, cytosolic, and mitochondrial cell fractionations of ATR-KD and PINK1-KD HCT116 cells. Vinculin and ATP5A were used to control protein loading in the corresponding fractionation. *n* = 3. (**B**) Western blot analysis of the indicated proteins in HCT116 cells after ATR-KD treated or not with 10 μM of the proteasome inhibitor MG-132 for 4 h. α-tubulin was used to control protein loading. *n* = 3. (**C**) Co-IPs using a PINK1 antibody in cytosolic (cyto) and mitochondrial (mito) fractionations of HCT116 cells followed by Western blotting using the indicated antibodies. Vinculin and ATP5A were used to control protein loading within the corresponding inputs. *n* = 3. (**D**) Co-IPs of PINK1 with ATR in WCEs of HCT116 cells after siRNA or siATR treatment followed by Western blotting using the indicated antibodies. VE821 is an ATR inhibitor. Vinculin was used to control protein loading in input. *n* = 3. (**E**) Immunofluorescence microscopy by anti-ATR and anti-PINK1 antibody staining of ATR co-localization with PINK1 within mitochondrial networks in HCT116 cells. The mitochondrial networks (MitoTracker staining) and the co-localization of ATR with mitochondria (arrow in mid panels) or PINK1 (arrow in right panels) are shown. M: mitochondrion; N: nucleus. *n* = 3.

While investigating if ATR deficiency affects mitophagy directly, we found a great reduction of the upstream mitophagy executers PINK1 in ATR-iKO MEFs (Fig. 1G). Likewise, siATR downregulated PINK1 in HCT116 cells (Fig. 2A). Reciprocally, PINK1 KD also reduced the ATR level in the mitochondria fraction as well as in cytosol and whole cell extracts (WCEs) (Fig. 2A). Treatment of ATR-deficient cells with the proteasome inhibitor MG-132 restored the precursor of PINK1 and its cleavage isoform (Fig. 2B), which is usually dedicated for proteasome degradation during its turnover [9, 10, 55]. These findings suggest a role for ATR in PINK1 stabilization.

Next, we performed co-immunoprecipitation (Co-IP) experiments using HCT116 cells to address whether ATR would interact with PINK1. IPs against PINK1 could pull down a profound amount of ATR in the mitochondrial fraction (Fig. 2C). Of note, siATR, but not the ATR kinase inhibitor VE821, abolished ATR–PINK1 interactions (Fig. 2D). Furthermore, IPs against FLAG in HEK293T cells transfected with either FLAG-ATR-WT (wild type) or FLAG-ATR-KiD (ATR kinase-dead mutant) showed equal binding of wild-type and kinase-dead mutant ATR with GFP-PINK1 (Supplementary Fig. S4A). Fluorescence microscopy revealed that ATR co-localized together with PINK1 within mitochondrial networks marked by MitoTracker (Fig. 2E). Collectively, these results indicate a physical interaction between ATR and PINK1, independent of ATR kinase activity.

### ATR anchors at mitochondrial membrane via TOM/TIM

**Figure 3.**
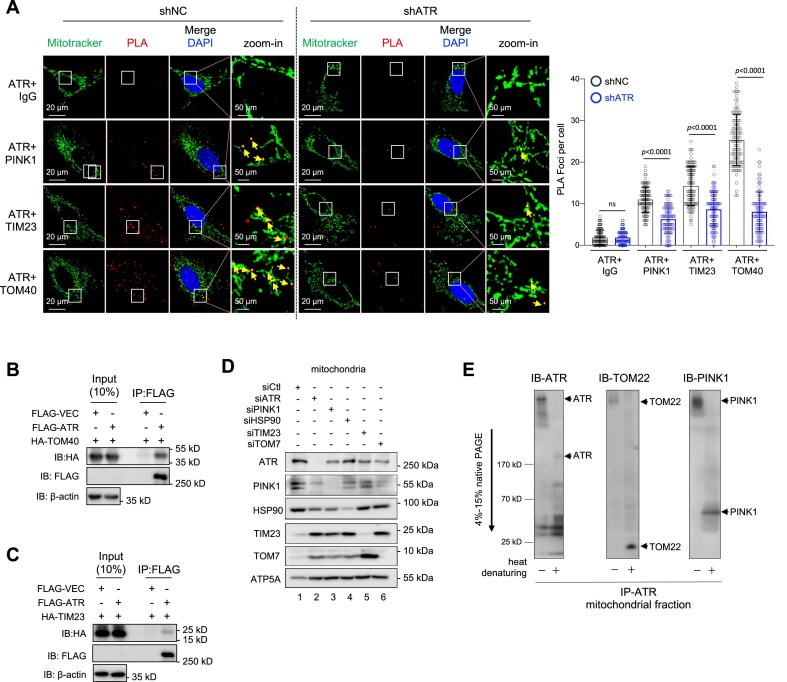
ATR docks at mitochondria by direct interaction with PINK1 and TOM/TIM. (**A**) PLAs of ATR interaction with PINK1, TIM23, and TOM40 in U2OS cells without (shNC) or with ATR-KD (shATR). Left panel: Representative images of PLA-positive signals (red foci) after co-staining with mitochondrial marker MitoTracker. Yellow arrows indicate PLA foci in mitochondria. Right panel: Quantification of PLA-positive foci from >200 cells scored. Statistics were performed using a two-way ANOVA with a Tukey’s multiple comparisons test. *n* = 3. (**B**) Co-IPs by the FLAG antibody in FLAG-ATR and HA-TOM40 transfected HEK239T cells followed by western blot analysis of the indicated proteins. β-actin was used to control protein loading. *n* = 3. (**C**) Co-IPs by the FLAG antibody in FLAG-ATR and HA-TIM23 transfected HCT116 cells followed by western blot analysis of the indicated proteins. β-actin was used to control protein loading. *n* = 3. (**D**) HCT116 cells with targeted siRNA against ATR (siATR), PINK1 (siPINK1), HSP90α (siHSP90), TIM23 (siTIM23), and TOM7 (siTOM7) are analyzed by Western blotting for the indicated proteins in mitochondrial fractionations. ATP5A was used to control protein loading. *n* = 3. (**E**) 1D BN–PAGE analysis of ATR, TOM22, and PINK1 from the purified mitochondrial fraction of N2A cells. Under native conditions, ATR, TOM22, and PINK1 migrate at the same speed in native PAGE gel. After heat treatment, denatured samples were pulled down by an anti-ATR ab. The native and denatured samples were blotted with the indicated antibodies, which detect the respective proteins in denatured gels (lane “heat denaturing +”). *n* = 3.

TEM and fractionation experiments located ATR at mitochondria; yet, ATR has no mitochondrial targeting signal (MTS) sequence according to the MitoMiner4.0 database [56]. PINK1 locates at mitochondria via its MTS where it interacts with the mitochondrial translocases TOM/TIM complex members TOM7, TOM40, and TIM23 [57, 58, 55]. We found an interaction of ATR and PINK1. These notions raised an attractive scenario that ATR via its interaction with PINK1 locates at mitochondria. To test this hypothesis, we first performed an endogenous PLA in human U2OS cells including co-staining with MitoTracker, to visualize the co-localization of ATR and PINK1 at mitochondria together with TOM40 and TIM23. ATR formed a considerably high level of PLA foci with PINK1 compared with IgG controls (Fig. 3A). Notably, ATR also formed very high levels of PLA foci together with TOM40 and TIM23 at mitochondria (Fig. 3A). shATR knockdown (Supplementary Fig. S4B) however greatly reduced PLA signals of ATR with TOM40 and TIM23, as well as with PINK1 (Fig. 3A). Furthermore, Co-IP experiments in GFP-ATR and HA-TOM40 or HA-TIM23 transfected HEK293T cells corroborated the interaction of ATR with TOM40 and TIM23 (Fig. 3B and C). Interestingly, RNAi-based knockdown of TOM7 and TIM23 in HCT116 cells reduced PINK1 at mitochondria, as expected, and, simultaneously, ATR (Fig. 3D). Furthermore, consistent with the notion that both ATR and PINK1 are known clients of HSP90 [27, 28, 59, 60], the HSP90α antibody pulled down both ATR and PINK1 (Supplementary Fig. S4C and D) and HSP90α knockdown decreased the presence of both ATR and PINK1 at mitochondria (Fig. 3D). To further examine endogenous ATR localization at the mitochondrial membrane, we performed a BN–PAGE assay using an anti-ATR antibody to pulldown complexes from the purified mitochondrial fraction and detected a direct interaction of endogenous ATR with TOM22, as well as with PINK1 (Fig. 3E). Taken together aforementioned results, both ATR and PINK1 are brough by HSP90 to mitochondria where they dock at the TOM/TIM translocase complex.

### ATR stabilizes PINK1 at mitochondria and enables mitophagy

**Figure 4.**
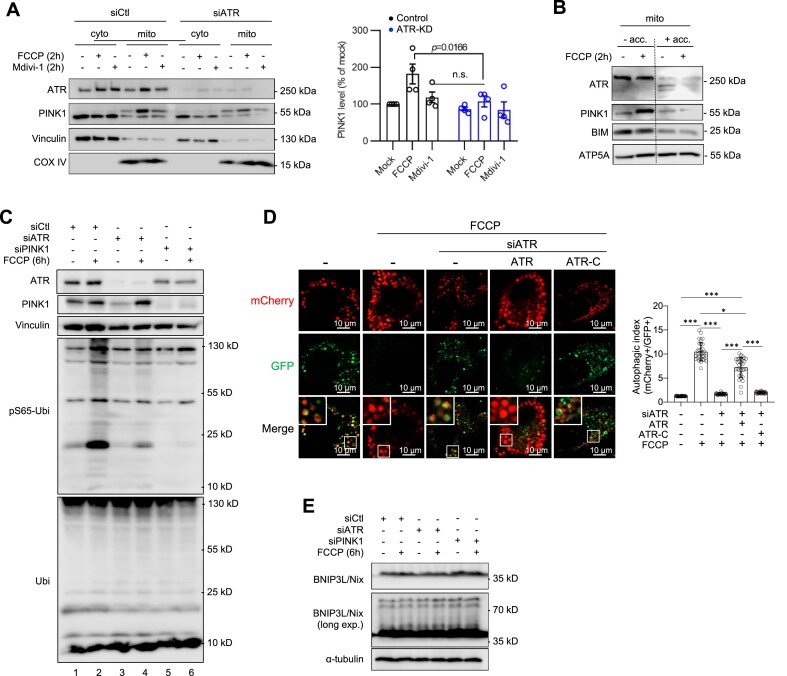
ATR together with PINK1 resides at the mitochondrial membrane for mitophagy. (**A**) Mitophagy initiation of HCT116 cells treated with either (siCtl) or siATR (ATR-KD) was induced by 2 h treatment of 2 μM FCCP or blocked by 5 μM Mdivi-1. Western blot analysis of the indicated proteins in cytosolic and mitochondrial cell fractionations. COX IV and vinculin were used to control protein loading in the corresponding fractionation. Quantification of mitochondrial PINK1 was done using ImageJ. Error bars show SEM. The statistical analysis was performed using two-tailed *t*-test. *P*-values are indicated within individual graphs. n.s.: not significant. *n* = 4. (**B**) Western blot analysis of mitochondrial fractionation of HCT116 cells treated with 2 μM FCCP for 2 h and blotted with the indicated proteins. Gentle treatment by Accutase (acc) is to remove proteins from mitochondrial surfaces. BIM was used as the indicator of outer surface-bound mitochondrial proteins. ATP5A serves as an inner membrane marker. *n* = 3. (**C**) ATR-KD (siATR) and PINK1-KD (siPINK1) HCT116 cells were treated with FCCP for 6 h to induce mitophagy and analyzed by Western blotting using the indicated antibodies. Vinculin was used to control protein loading. *n* = 3. (**D**) Mitophagy induced by 1 μM FCCP in mCherry-EGFP-LC3B stably transfected U2OS cells after siATR KD, which were reconstituted with wild-type or mutant ATR-C that is deficient for PINK1-binding (see Supplementary Fig. S6H). Autophagic vesicles (puncta) were analyzed by microscope for the indicated fluorescence colors. FCCP induces autophagy as visualized by an increase of mCherry^+^-only puncta because of GFP^+^ signal quench in autolysosome. Right panel: The autophagic index indicates the ratio of the number of mCherry^+^ puncta to GFP^+^ puncta. More than 90 cells from three single cell lines were analyzed. Statistics were performed using paired Student’s *t*-test. *P < .05; ***P < .001. (**E**) Western blot analysis of ATR-KD and PINK1-KD HCT116 cells after treatment with FCCP for 6 h for the indicated proteins. α-tubulin was used to control protein loading. *n* = 3.

PINK1 is a master regulator of mitophagy in response to mitochondrial injury or depolarization [9, 10]. We next investigated the meaning of ATR–PINK1 interaction at the mitochondrial membrane. Because ATR-deleted cells were refractory to mitophagy, we first explored the potential mechanism by which ATR is involved in PINK1-mediated mitochondrial stress response. To this end, we treated cells with mitophagy inducer FCCP, which disrupts the MMP and stabilizes PINK1 at mitochondria. Western blotting showed that the FCCP-induced mitochondrial accumulation of PINK1 was greatly attenuated when ATR was knocked down by siRNA, which behaved similarly to cells treated with the Drp1 inhibitor Mdivi-1 that represses PINK1 recruitment to mitochondrial membranes [61] (Fig. 4A). While FCCP elevated PINK1 levels, as expected, it also concurrently increased ATR in mitochondria (Fig. 4A). PINK1 accumulation at the mitochondrial outer membrane is a key event for mitophagy initiation [9, 58]. Then, we examined the situation of PINK1 and ATR at the outer membrane after mitophagy induction by FCCP. To this end, we mildly treated mitochondrial fractions with the protease Accutase, which preserved inner membrane proteins judged by ATP5A, simultaneously removed ATR and PINK1 together with the outer membrane-bound protein BIM from mitochondria (Fig. 4B). These observations, together with the Co-IP, PLA, and native PAGE data, indicate that ATR helps PINK1 stabilization at the outer mitochondrial membrane in response to mitochondrial injury.

To further investigate whether ATR would modulate PINK1’s function in mitophagy, we monitored mitophagy activation in HCT116 cells by analyzing phosphorylation of PINK1 substrate ubiquitin (pS65-Ubi), an early step of PINK1-mediated mitophagy [57, 9, 62]. As expected, FCCP treatment in wild-type cells induced a strong pS65-Ubi signal (ladders), which however was greatly repressed when ATR was knocked down (Fig. 4C, lanes 2 and 4). Further, we performed fluorescence microscopy of pMEFs and found that FCCP induced co-localization of PINK1 and LC3B at mitochondria, indicating mitophagy initiation [62] (Supplementary Fig. S4E; yellow arrows), which however was abolished by ATR deletion, similar to the samples treated by Mdivi-1 (Supplementary Fig. S4E). Next, we adopted a dual-fluorescence autophagy reporter assay in conjugation with FCCP treatment to monitor mitophagy [63]. To this end, we engineered stable U2OS cell lines after transfection with the mCherry-EGFP-LC3B vector. After puromycin selection, five cell lines with a stable expression of mCherry-EGFP-LC3B were established and confirmed by double positivity for GFP and mCherry (denoted as CEL lines). We used siRNA to knockdown ATR in three CEL cell lines to explore the involvement of ATR in mitophagy. FCCP induced a high level of the autophagic index (ratio of mCherry+ versus GFP+ puncta, indicative of the fusion of LC3-containing vesicles with lysosomes), which was greatly repressed by ATR knockdown (Fig. 4D), indicating that ATR depletion compromises mitophagy. This mitophagy defect, however, could be significantly reversed by overexpression of full-length ATR, but not by ATR-C mutant deficient of PINK1-binding (Fig. 4D and Supplementary Fig. S6H). The ubiquitin- and PINK1-independent mitophagy pathway, namely BNIP3L/Nix [62] was apparently unaltered in ATR-KD cells (Fig. 4E), meaning that it could not compensate for the defective PINK1-mediated mitophagy in ATR mutant cells. Altogether, ATR is required for PINK1-mediated mitophagy.

### ATR deletion disrupts the ETCC assembly affecting mitochondrial respiration

**Figure 5.**
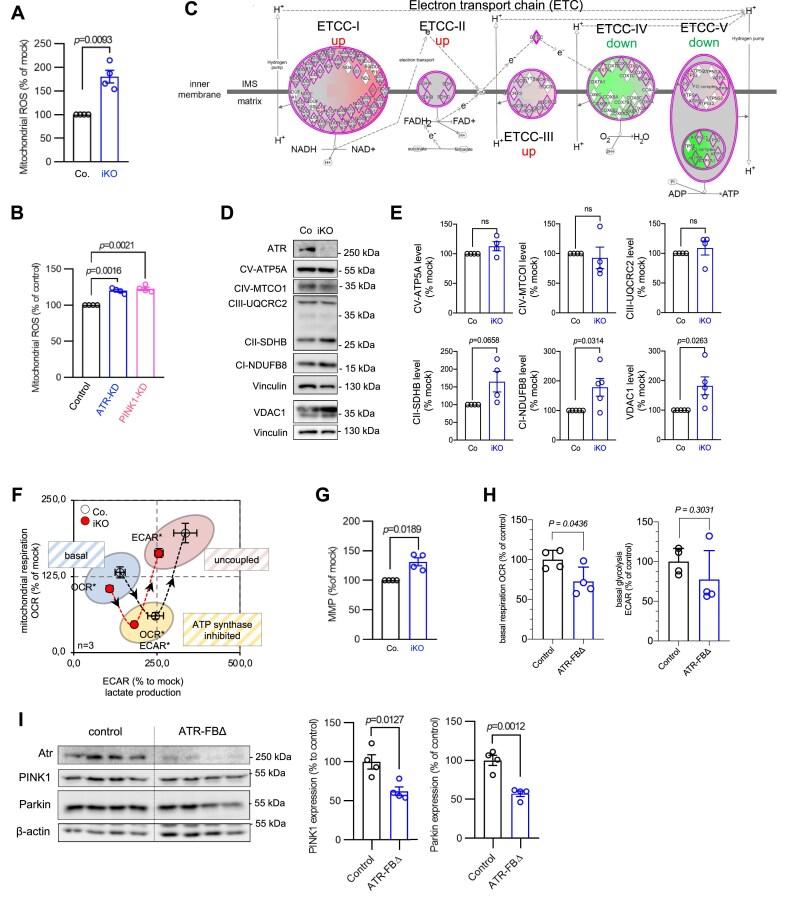
ATR deletion alters mitochondrial respiration and induces ROS. (**A**) The accumulation of mitochondrial ROS was determined by flow cytometry analysis of MitoSox at 5 dpo. *n* = 4. (**B**) The mitochondrial ROS in ATR-KD and PINK1-KD HCT116 cells were analyzed by flow cytometry of Mitosox. *n* = 4. (**C**) Proteome changes in ATR-iKO pMEFs were analyzed using MS. Graphical representation of altered ETC complex (ETCC) in ATR-iKO pMEFs (refer to Supplementary File S1). Upregulated proteins are displayed in red and downregulated proteins in green. The color intensity reflects the value of changes in the complex. Three ATR-wild type and three ATR-iKO cell lines were analysed. (**D**) Western blot analysis of ETCC subunits in pMEFs. For the detection of ETCC I-V, a total OXPHOS rodent WB antibody cocktail was used. Vinculin was used to control the protein loading. (**E**) Quantification of the protein levels by ImageJ. *n* = 4. (**F**) Analysis of mitochondrial respiration of ATR-iKO pMEF at 5 dpo. The metabolic phenotype was generated from Cell Mito Stress Test using a Seahorse analyzer. The graphs show the OCR plotted against ECAR from the “basal” state into oligomycin treatment (ETCC-V inhibitor, “ATP synthase inhibited”) and FCCP uncoupling (“uncoupled”). *n* = 3. (**G**) The MMP was determined by flow cytometry analysis of DiOC_6_(3) at 5 dpo. *n* = 4. (**H**) The basal OCR and ECAR of ATR-FBΔ hippocampi were analyzed using a Seahorse XFe24 Analyzer. *n* = 4. (**I**) Western blot analysis of the indicated proteins in ATR-FBΔ and control hippocampi. β-actin was used to control protein loading. The right panel shows the quantification of the protein levels using ImageJ. *n* = 4. The error bars show SEM. Statistics were done using a two-tailed Student’s *t*-test. *P*-values are indicated within individual graphs.

It is well known that PINK1 activation and subsequent mitophagy specifically eliminate mitochondria with high ROS levels, preventing the spread of oxidative damage to cellular components [8, 14, 15]. Consistent with this notion, we found that ATR-iKO pMEFs (Fig. 5A) as well as ATR-KD HCT116 cells (Fig. 5B) contained significantly higher levels of mitochondrial superoxide (O_2_^−^) radicals (mitochondrial ROS (mtROS), ∼20%) compared with controls, which phenocopies PINK1-KD cells (Fig. 5B).

To investigate the basis of mtROS overproduction in ATR-deficient cells, we measured mitochondrial respiration. To this end, we first performed a quantitative MS to analyze the proteome of ATR-iKO and control pMEFs. IPA (cutoff *q* < 0.05) of the proteome dataset revealed mitochondrial dysfunctions as the second-most significant process affected, together with the altered MMP and mitochondrial biogenesis (−log(*P*-value) > 1.3) (Supplementary Fig. S5A). Notably, a general disturbance of mitochondrial proteins, prominently of the ETC, was evident after ATR deletion (Supplementary File S1 and Fig. 5C). The subunits of ETCC-I, -II, and -III were more abundant in ATR-iKO pMEFs compared with controls (Fig. 5C), which correlate well with high levels of ROS production in ATR mutant cells [64, 65]. Western blot analysis using available antibodies detected an alteration of some ETCC subunits (Fig. 5D and E). In addition, the outer membrane pore protein VDAC1, which facilitates mtROS release, was highly upregulated in ATR-iKO pMEFs (Fig. 5D and E). Interestingly, VDAC1 is a primary target of PINK1/Parkin-mediated ubiquitination [66, 67] and its level reversely correlated with a low PINK1 level (see Fig. 1G).

The imbalanced expression of ETCC proteins often impair respiration [5, 68]. Concurrently, Cell Mito Stress Test using a Seahorse analyzer revealed that ATR-iKO pMEFs had a significantly lower basal level of mitochondrial respiration (OCR) and cellular lactate secretion (ECAR), compared with controls (Fig. 5F and Supplementary Fig. S5B). This was accompanied by a significantly higher MMP in ATR-iKO cells (Fig. 5G) and ATR-KD HCT116 (Supplementary Fig. S5C), as well as in PINK1-KD cells (Supplementary Fig. S5C), compared with controls. All these data indicate that ATR deficiency, mirroring PINK1 defects, is associated with mitochondrial dysfunction.

### ATR deletion causes mitochondrial dysfunction in the postmitotic brain tissue

Neural cells have a highly active glycolysis and respiration rate, and thus rely on robust mitochondrial functionality [69–77]. To further address the mitochondrial functions of ATR in neural tissues, which represent a major target organ of ATR-SS patients, we generated mice in which ATR was deleted in the pyramidal neurons of the forebrain of *Atr*^f/f^ mice by expressing CamKII-Cre transgene (ATR-FBΔ mice) [31]. Seahorse analysis of ATR-FBΔ hippocampus biopsies revealed a significant reduction of basal OCR, as well as a trend of reduced basal ECAR, compared with controls (Fig. 5H). Like in cellular models, the expression of PINK1 in the hippocampus of ATR-FBΔ brains was significantly reduced (Fig. 5I). Thus, the mitochondrial defects caused by the ATR deletion are common between different cell types and, importantly, also present in postmitotic tissues, highlighting a fundamental importance of ATR–PINK1 in mitochondrial homeostasis.

### ATR interacts with the activation domain of PINK1 to support mitophagy

**Figure 6.**
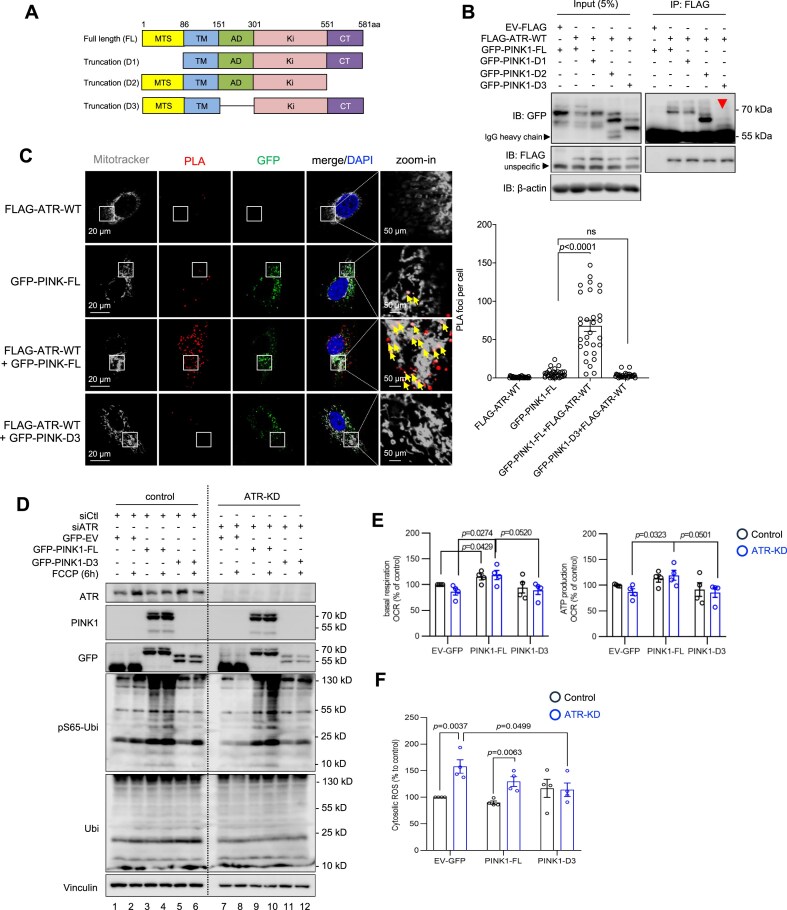
Interaction of PINK1 and ATR is required for mitophagy initiation. (**A**) Schematic view of PINK1 full length and truncation mutants D1–D3. Abbreviations: mitochondrial targeting signal (MTS), transmembrane domain (TM), activation domain (AD), kinase domain (Ki), and C-terminus (CT). The amino acids (aa) of the border of the indicated domains are marked on the top of the full length PINK1 protein (FL). (**B**) FLAG-tagged empty vector (FLAG-EV), ATR wild type (FLAG-ATR-WT), GFP-tagged full length PINK1 (GFP-PINK1-FL), and the PINK1 domain truncation mutants D1–D3 were transfected into human HEK293T cells, respectively. Western blotting was performed on IPs of FLAG using the indicated antibodies. β-actin was used to control protein loading in input. Red arrow indicates the missing band of Co-IP of GFP-PINK1-D3. *n* = 3. (**C**) PLAs of FLAG-ATR-WT, GFP-PINK1-FL, and GFP-PINK1-D3 in human U2OS cells. Representative images of PLA-positive signals (red foci) are shown. Mitochondrial networks (gray) are visualized by MitoTracker staining. Yellow arrows marked PLA foci in the mitochondrial network (gray). Right panel: Quantification from 30 randomly chosen cells per group were performed manually. Error bars show SEM. Statistics are performed with a two-way ANOVA with a Tukey’s multiple comparisons test. n.s.: not significant. *n* = 3. (**D**) Western blot analysis of transiently transfection of FLAG-EV, GFP-PINK1-FL, and GFP-PINK1-D3 into RNAi-induced ATR-KD HCT116 cells following by 2 μM FCCP treatment for 6 h. The indicated proteins were analyzed by respective antibodies. Vinculin was used to control protein loading. *n* = 3. (**E**) Mitochondrial respiration analysis using a Cell Mito Stress Test of HCT116 cells after transfections with the indicated vectors. The basal respiration and ATP production were calculated from OCR datasets. *n* = 4. (**F**) The cytosolic ROS were determined by flow cytometry analysis after cells incubating with 2 μM 6-carboxy-2′,7′-dichlorodihydrofluorescein diacetate. *n* = 4. Error bars show SEM. The statistical analysis was performed using two-tailed *t*-test. *P*-values are indicated within individual graphs.

To understand the structure–function basis of the ATR–PINK1 interaction, we first performed molecular modeling to predict domains in PINK1 (PDB used: 6EQI) that interact with ATR (PDB used: 5YZ0) using the SWISS-MODEL workspace [70, 71]. We used PINK1-FL (full length) and the truncation mutants PINK1-D1, -D2, and -D3 [9, 55] (Fig. 6A). PINK1-FL has several α-helices and a central β-sheet domain (Supplementary Fig. S6A); the latter was changed only moderately in PINK1-D1 and -D2 mutants (Supplementary Fig. S6B and C), but strongly in the PINK1-D3 mutant that lacks the central activation domain (AD) (Supplementary Fig. S6D). The N-terminal ATR mainly consists of α-helices and the interaction surface for its genuine partner ATRIP [72] (Supplementary Fig. S6E), an essential site for its nuclear DDR function. The SPRING server [73] predicted a high probability of the N-terminal ATR binding with the β-sheet domain of PINK1-FL, but not with PINK1-D3 lacking the AD domain (Supplementary Fig. S6F and G). Indeed, Co-IP experiments confirmed an interaction of PINK1-FL predominantly with the N-terminus, but less or without obvious interaction with the middle part and the C-terminus of ATR (Supplementary Fig. S6H).

We next investigated the interaction of ATR and PINK1 and its impact in mitophagy. Consistent with the model prediction, FLAG-ATR bound GFP-tagged PINK1-FL, -D1, and -D2, but not PINK1-D3 in HEK293T cells (Fig. 6B). Moreover, the PLA assay revealed that ATR co-transfection with PINK1-D3 yielded no PLA foci in human U2OS cells in contrast to a very high level of PLA foci formed by ATR and PINK1-FL (Fig. 6C; see also Fig. 3A). Next, we examined if this specific interaction is required for PINK1-mediated mitophagy by western blotting of pS65-Ubi. Forced expression of PINK1-FL, but not PINK1-D3, overrode the mitophagy defects of ATR-KD cells (Fig. 6D), indicating that ATR binding PINK1 via its AD domain, is necessary for PINK1 stabilization and thereby its mitophagy execution. This conclusion is further supported by the mitophagy reporter assay showing that PINK1-binding mutant ATR (ATR-C) was insufficient for mitophagy execution (see Fig. 4D). Moreover, Seahorse analysis revealed that reconstitution of ATR-KD cells with PINK1-FL, but not PINK1-D3, restored the mitochondrial respiration and mitochondrial ATP production (Fig. 6E) and ameliorated ROS overproduction (Fig. 6F). Taken together, ATR interaction with PINK1 is required for mitophagy that secures proper mitochondrial functionality.

### ROS hyperproduction in ATR-deficient cells causes oxidative damage primarily in cytosol and mitochondria compartment, but not nDNA

**Figure 7.**
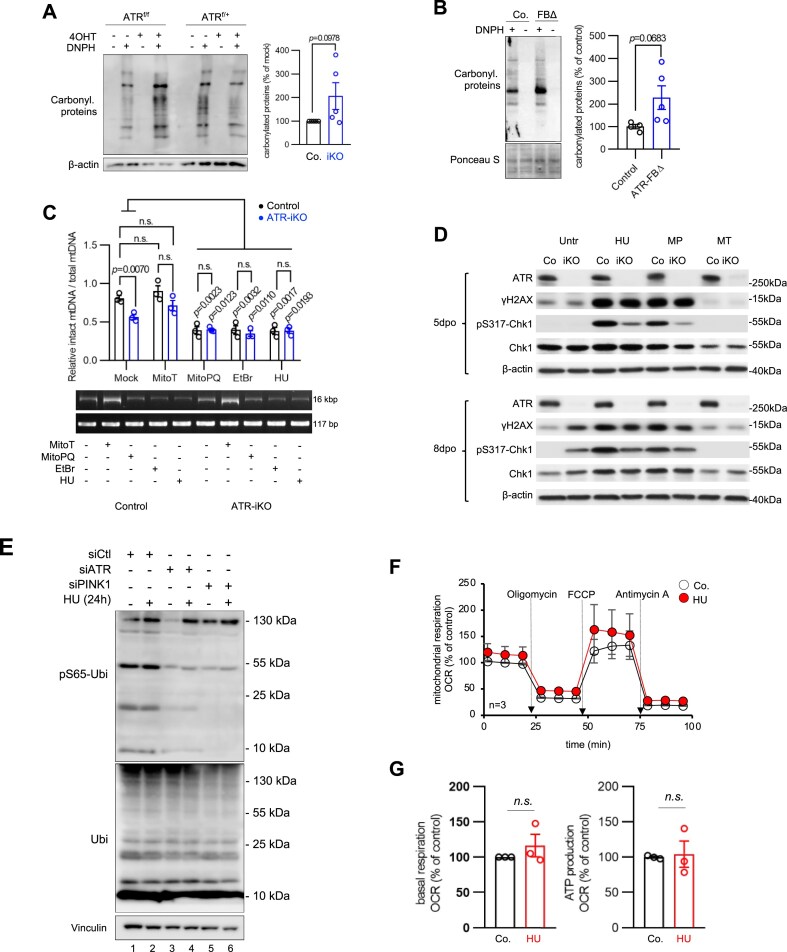
Cross talk of dysfunctional mitochondria and nuclear DDR in ATR-deleted cells. (**A**) Protein carbonylation was analyzed by western blotting in ATR-iKO pMEFs at 5 dpo and quantified using ImageJ. Oxidized proteins were detected via the incorporation of DNPH. β-actin is used to control protein loading. *n* = 5. (**B**) Brain lysates of 3-month-old ATR-FBΔ mice were analyzed by western blotting. Oxidized proteins were detected via the incorporation of DNPH. Ponceau S staining controls the protein loading. The quantification of carbonylated proteins was determined using ImageJ. *n* = 5. (**C**) Analysis of mtDNA damage in ATR-iKO pMEF at 5 dpo and 24 h after treatment with 10 μM of the ROS scavenger MitoT, 10 μM of the ROS inducer MitoPQ, 450 ng/ml of the mtDNA-damaging agent EtBr, and 0.2 mM of HU. mtDNA breaks were analyzed by PCR. The quantification of the 16-kb PCR product was performed using ImageJ. *n* = 3. (**D**) Western blotting of ATR-iKO pMEFs at 5 dpo (initiation phase of mitochondrial dysfunctions) and 8 dpo (post-mitochondrial dysfunctions) were treated 24 h before analysis with 0.2 mM HU, 10 μM MitoPQ (MP), 10 μM MitoT (MT), and 5 μM UK5099 (UK, MPC inhibitor). The indicated proteins were blotted by respective antibodies. β-actin was used to control protein loading. *n* = 3. (**E**) Mitophagy initiation assay in ATR-KD and PINK1-KD HCT116 cells after exposure to 0.5 mM HU for 24 h followed by western blotting of the indicated proteins. Vinculin was used to control protein loading. *n* = 3. (**F**) Mitochondrial respiration analysis of HCT116 cells treated with 0.5 mM HU for 24 h, using a Cell Mito Stress Test. *n* = 3. (**G**) Quantification of the basal respiration and ATP production of HCT116 cells were calculated from OCR (see panel F). *n* = 3. Error bars in all subfigures show SEM. The statistical analysis was performed using two-tailed *t*-test. *P*-values are indicated within individual graphs. n.s.: not significant.

ATR conducts an early step of the nuclear DDR [1, 23] and nDNA damage could affect mitochondrial function that disturb mitochondrial ROS production, which can in turn elicit retrograde the nuclear DDR [74]. To differentiate if mitochondrial defects in ATR-deleted cells would be secondary to the nuclear DDR, we took advantage of the inducible deletion of ATR in ATR-iKO pMEFs and performed a series of time course experiments (see Supplementary Fig. S2A and B). We first monitored whether ROS overproduction causes oxidative damages at 5 dpo when mitochondrial defects were already evident in ATR-iKO pMEFs (see Fig. 1E and F and Supplementary Fig. S2C–E) and found a higher level of oxidized proteins in ATR-iKO pMEFs compared with controls (Fig. 7A), judged by the amount of carbonylated proteins [2,4-dinitrophenylhydrazone (DNPH) incorporation] [75]. Similarly, an increase of oxidized proteins was also detected in the cortices of 3-month-old ATR-FBΔ mice (Fig. 7B). At the same timepoint, we measured mtDNA damage using a PCR-based approach [46]. The number of amplicons derived from the intact mtDNA genome (16 kb) was lower in ATR-iKO cells compared with controls (Fig. 7C), reflecting a high level of mtDNA breaks. Interestingly, the mtROS scavenger MitoTempol (MitoT) ameliorated mtDNA breaks (Fig. 7C). As a control, the mtROS inducer MitoParaquat (MitoPQ) and HU (inducing DNA breaks) as well as EtBr (degrading mtDNA templates) all significantly reduced the number of mtDNA amplicons (Fig. 7C). Although ATR plays a role in solving R-loop that formed during transcription of the nuclear genome [2, 76], RNA-seq analysis did not detect overt alteration of mitochondria-encoded transcripts in ATR-deficient cells (Supplementary Fig. S7A), suggesting a neglectable role of ATR in transcription of the mitochondrial genome.

Despite the accumulation of mtDNA damage at 5 dpo, fluorescence microscopy of the sensitive nDNA damage marker PAR, which is synthesized by PARP1 in response to SSB or replicative stress [77], did not detect difference between ATR-iKO pMEFs and controls (Supplementary Fig. S7B). Nonetheless, the PAR level was elevated in ATR-iKO pMEFs after short exposure to the pro-oxidant tBHP, indicating fully functional PARP1-mediated DNA damage recognition without ATR. At this stage, ATR-iKO pMEFs showed normal DDR hallmarks, such as apoptosis (Annexin-V+ cells) (Supplementary Fig. S7C) or senescence (β-galactosidase+ cells) (Supplementary Fig. S7D). In addition, western blot analysis showed only low and, more importantly, similar amounts of the nDNA damage marker γH2AX in both ATR-iKO and control cells at 5 dpo (Fig. 7D). Similar to HU, the ROS inducer MitoPQ induced γH2AX signals irrespective of the ATR status (Fig. 7D) and also phosphorylated Chk1 (p-Chk1, an ATR substrate), which was however lower in ATR-iKO cells, as expected (Fig. 7D). Interestingly, MitoT and the inhibitor of the mitochondrial pyruvate carrier (MPC) UK5099 that represses the tricarboxylic acid (TCA) cycle activity fuelling the OXPHOS, abolished γH2AX and p-Chk1 signals (Fig. 7D). All these findings demonstrate that when ATR deletion already altered mitochondrial function, nuclear DDR signaling remained silent at this stage.

### Mitochondria-generated ROS in ATR-deleted cells eventually damage nDNA

We then examined the cross talk between mtROS and nDNA at late timepoints after ATR deletion. At 8 dpo, ATR-iKO pMEFs had a higher level of γH2AX and p-Chk1 than controls (Fig. 7D). Intriguingly, MitoT and UK5099 blunted γH2AX signals and p-Chk1 in both ATR-iKO pMEFs and controls (Fig. 7D), indicating that mitochondria-derived ROS are the primary source of nDNA damage in ATR-iKO cells. Although p-Chk1 in untreated ATR-iKO pMEFs could occur via ATR-independent DDR pathways, e.g. ATM or DNA-PK [78, 79], p-Chk1 in response to HU or MitoPQ was expectedly impaired in ATR-iKO pMEFs (Fig. 7D). At later stages, the DDR defects in mutant cells became more prominent: we saw more apoptotic cells at 10 dpo (Supplementary Fig. S7C) and more senescent cells at 15 dpo (Supplementary Fig. S7D) in ATR-iKO cultures, compared with controls.

Finally, we tested whether nDNA damage would be responsible for mitochondria defects observed in ATR-deleted cells. We treated HCT116 cells with HU, a genuine activator of ATR to elicit nuclear DDR, and analyzed mitochondrial stress response. HU treatment did not trigger PINK1-mediated pS65-Ubi signals (Fig. 7E, lanes 1 and 2), nor altered mitochondrial respiration and ATP production (Fig. 7F and G). Thus, the mitochondrial phenotype of ATR-deficient cells is unlikely a direct consequence of nDNA damage originated from ATR-mediated DDR defects.

## Discussion

ATR is a well-established nuclear protein safeguarding the genome integrity by conducting the DDR in response to replication fork stall and SSBs [1–3]. Intriguingly, we find a wide spread of ATR localization in many subcellular compartments. We focused only on ATR’s role in mitochondria in this study and hence cannot rule out (perhaps rather implicate) possible roles of ATR in modulating cellular activity of other organelles. Here, we discover that ATR is a physiological component of PINK1-mediated MQC program, thereby modulating mitochondrial homeostasis and functionality.

Although ATR does not harbor a classical MTS, its mitochondrial localization is achieved by interaction with PINK1 docking at the TOM/TIM complex on the mitochondrial membrane. In unperturbed mitochondria, PINK1 precursor associates with TOM/TIM followed by cleavage at the inner mitochondrial membrane, to release the matured isoform into the cytosol for proteasome degradation [9, 58, 80]. TOM/TIM-mediated importation of mitochondrial proteins is crucial for the mitochondria maintenance and respiration: if TOM/TIM channels are impaired, nonimported mitochondrial precursor proteins often undergo proteasome degradation [81]. For example, downregulation of TOM20/TIM23 results in OXPHOS defects and accumulation of protein aggregates in the mitochondrial matrix, which assemble a cause of pathogenesis in PD [82]. Intriguingly, ATR is also associated with TOM/TIM in healthy mitochondria and, congruently, TOM/TIM knockdown reduces the presence of ATR (unknown before) as well as PINK1 (known) at mitochondria. ATR knockdown also downregulates the PINK1 level. Thus, it is plausible that ATR binding PINK1 at TOM/TIM, via its scaffold role, helps PINK1 stabilization when mitophagy elicits [11, 58, 83, 84].

The mitochondrial defects of ATR-deficient cells, such as malformed mitochondria, impaired mitochondrial respiration, and ROS overproduction phenocopy well PINK1-deficient cells (see Fig. 5B and Supplementary Figs S2G and S5C). In this regard, cells deficient for ATM, a sister of ATR in the PIKK family, and a subgroup of A-T patient cells exhibit mitophagy defects, although the underlying mechanism is poorly understood [85, 86]. Despite the presence of malformed mitochondria and dysfunctional mitochondrial respiration that alters MMP and ATR loss, similar to ATR-C mutant deficient in interaction with PINK1, restrains mitophagy initiation, analog to PINK1 deficiency or ATR interaction mutant PINK1-D3 that lacks the AD domain (Figs 4C and D and 6D). The AD domain is within the kinase domain [87] and the Ser228 residue in this region is important for PINK1 activation [42, 88]. Thus, we cannot completely rule out that the lack of rescue by PINK1-D3 in mitophagy could simply be due to a disrupted PINK1 kinase activity in this mutant. Nevertheless, the rescue effect by forced overexpression of full-length PINK1 and full-length ATR, but not ATR mutant (deficient for PINK1 binding), indeed argues for a role of ATR–PINK1 interaction, which stabilizes PINK1, in mitophagy execution. Thus, PINK1’s incapability of initiating mitophagy is because of lacking ATR assistance. Taking together, we conclude that ATR functions as an important partner of PINK1 in licensing PINK1-mediated mitophagy and safeguarding mitochondria homeostasis.

The defects of the MQC mechanism mitophagy are often associated with high levels of ROS from dysfunctional mitochondria [8–15]. ATR mutant cells, similar to PINK1-KD cells, harbor dysfunctional mitochondrial respiration generating aberrantly high mtROS, due to disturbed stoichiometry and assembly of ETCC [5, 68]. mtROS are a robust and prominent source of oxidative damage of both mtDNA and nDNA [4, 12]. We find that the high level of mtROS in ATR knockout cells primarily damages mtDNA before nDNA, reminiscent of a previous report showing that ROS-induced mtDNA damage can be an earlier event than nDNA damage [89]. Although mtDNA damage in ATR-deficient cells could be due to defects of potential ATR-mediated DNA repair, intriguingly, these mtDNA damage can be prevented by scavenging ROS, indicating that mtDNA damage is a result of oxidative damage originating from dysfunctional mitochondria. Thus, we can largely exclude a direct contribution of the DNA repair role of ATR in mitochondrial dysfunction. Nevertheless, ATR’s involvement in the MQC program can be in parallel or prior to the nuclear DDR. In this regard, it is worth noting that NBS1, a key component of the MRN (MRE11/RAD50/NBS1) complex in the DDR [90], has been shown to regulate Notch signaling during neuron arborization and migration, independent from its essential function in DNA repair [91].

ATR conducts the DDR after SSBs and replication fork stall. It is well known that DNA damage and DDR signaling can modulate mitochondrial functions [74, 92]. Yet, observations are often inconsistent, likely due to the experimental system applied. For example, the DDR regulator ATM can be activated by DNA damage, which then interferes with mitochondrial functions [93, 94]. However, others showed ATM activation by dysfunctional mitochondria [85] in the absence of DNA damage. Also, ATM can be directly activated by oxidation [95]. Yet, whether ATR can be directly activated by oxidation has not been reported. Of note, ROS can induce ATR downstream Chk1 phosphorylation independent of ATRIP/TopBP1/Claspin in *Drosophila* tracheoblast cells and also in *Xenopus* extracts via DNA repair protein APE2 [96, 97]. Remarkably, the nuclear DDR in ATR-deleted cells at a later stage can be largely repressed pharmacologically by scavenging ROS or blocking mitochondrial respiration. However, the DNA damaging agent HU, an ATR activator, does not injure mitochondria and had no overt impact on mitophagy in this experimental setting. Hence, uncontrolled mtROS, originated from defective mitochondria in ATR-deleted cells, are the origin of nDNA damage. These observations raise an interesting hypothesis that antioxidant agents may counteract the detrimental effects of malfunctional mitochondria and abnormal DDR to ameliorate the pathologies of ATR-SS patients and perhaps other DDR disorders.

We found PINK1 interaction predominantly with the N-terminus of ATR. The N-terminus of ATR provides the interaction surface for its genuine partner ATRIP which is required for nuclear DDR [72]. This interaction pattern raises an interesting scenario: as the protein–protein interaction site of ATR with ATRIP (for nuclear DDR) and PINK1 (for mitochondrial function), ATR could play a distinct role depending on its binding partner in the nucleus or cytosol, and also the physiological status. ATR’s kinase does not play a major role for ATR–PINK1 interactions, which however, does not rule out the possibility that small molecules that disrupt the interface of both proteins could alter mitochondrial functionality and metabolism, thereby presenting a sound approach for cancer treatment.

In summary, our study unravels a fundamental function of ATR outside of the nucleus. ATR, as an integral component of mitochondrial membrane-bound complexes, i.e. TOM/TIM, where it interacts and thereby stabilizes PINK1, licenses mitophagy to remove dysfunctional mitochondria and to prevent abnormal production of oxidative agent ROS that would otherwise damage other cellular components and initiate nuclear DDR (Supplementary Fig. S8). ATR in protecting the mitochondrial integrity and functionality could well be a primary task under the physiological status, particularly in postmitotic cells, which constitute most of physiological tissues in the body and which spares the essential function of ATR in replication stress. We here propose that ATR is a quality inspector and sits in a network to surveille nDNA damage (via CHK1) as well as mitochondrial quality (via PINK1). These two nonmutual exclusive mechanisms operate coordinately under the physiological and genotoxic conditions in a cell.

## Supplementary Material

gkaf178_Supplemental_Files

## Data Availability

The mass spectrometry proteomics data have been deposited to the ProteomeXchange Consortium (http://proteomecentral.proteomexchange.org) [98] via the PRIDE partner repository [99] with the data set identifier PXD013469. The RNA-seq data have been deposited in NCBI’s Gene Expression Omnibus [100] and are accessible through GEO Series accession number GSE244464 (https://www.ncbi.nlm.nih.gov/geo/query/acc.cgi?acc= GSE244464). Otherwise, all data generated or analyzed during this study are included in this published article (and its supporting information files).

## Notes

Present address: Francis Crick Institute, London, United Kingdom

Present address: Department of Cardiology, University Hospital Heidelberg, Heidelberg, Germany
