# Supplementary material for: DNA damage response regulator ATR licenses PINK1-mediated mitophagy
Source: Nucleic Acids Res. 2025 Mar 19;53(5):gkaf178. doi: 10.1093/nar/gkaf178 (PMC11920799; doi:10.1093/nar/gkaf178)
Supplement: gkaf178_Supplemental_Files [file gkaf178_supplemental_files.zip › RevNAR-SupplFig+Legend-final2.pdf]

Supplementary figures S1-S8
+ legends

Marx et al.

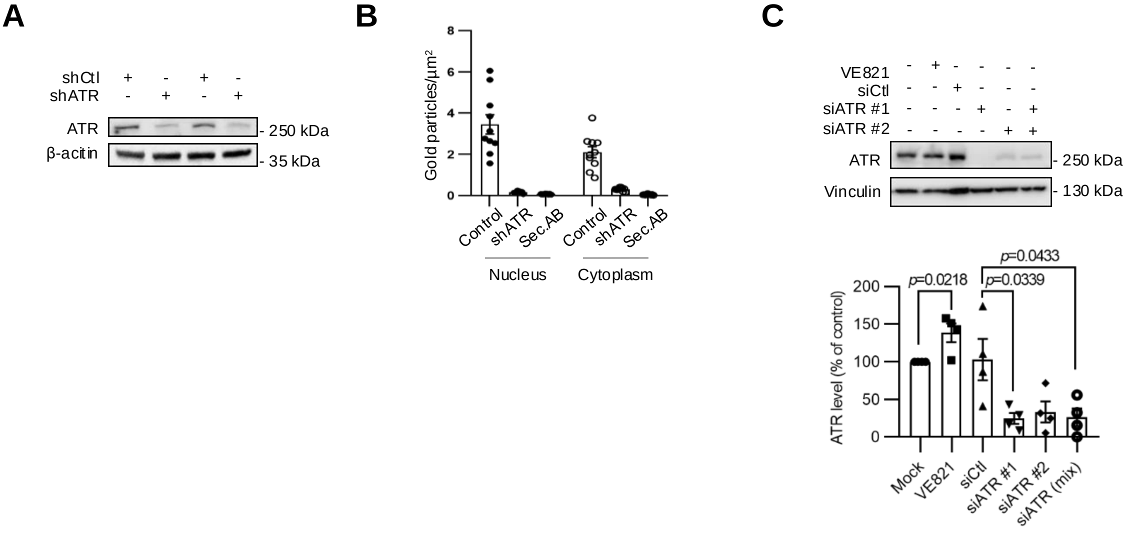

Supplementary figure S1: Localization of ATR in mitochondria. (A) Western blot analysis of ATR expression levels in HeLa cells transfected with shCtl (control) and targeted shRNA against ATR (shATR). β-actin was used to control protein loading. (B) Quantification of ATR-immunogold labeling in ATR-KD HeLa cells and controls. n=10. (C) Western blot analysis of the indicated proteins in HCT116 cells using RNAi with scrambled (siCtl), targeted siRNA against ATR1 (siATR#1, #2 or mixture of both (siATR mix)). The protein level was quantified by using ImageJ (lower panel). Vinculin was used to control protein loading. siATR#1 or mix of #1 and #2 siATR were used for all following experiments. n=4. Error bars show SEM. The statistical analysis was performed using two-tailed unpaired t test. *P*-values are indicated within individual graphs.

Figure S1-Marx et al.

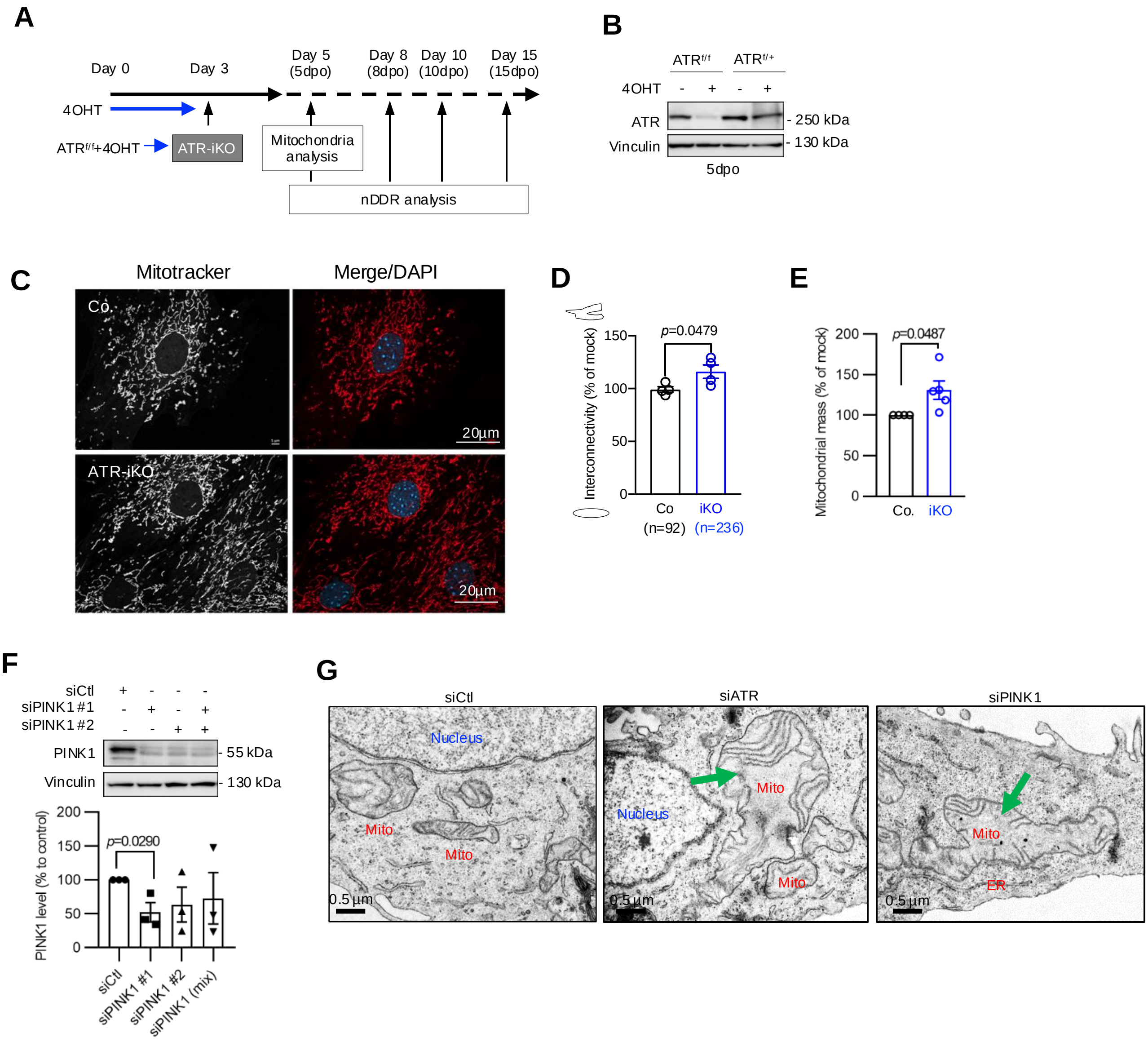

Supplementary figure S2: Deletion of ATR and PINK1 similarly causes mitochondrial malformations. (A) 4-OHT treatment and analysis regime of ATR-iKO pMEFs. ATR$^{f/f}$-CER pMEFs were incubated with 1 µM 4-hydroxytamoxifen (4-OHT) for 3 days (3 dpo) to induce ATR knockout (ATR-iKO). At the indicated days of the post 4OHT treatment (dpo) experiments were performed. (B) ATR expression levels were analyzed by Western blotting at 5 dpo. Vinculin was used to control protein loading. n=3. (C) The mitochondrial networks in ATR-iKO pMEFs and controls were visualized using Mitotracker staining and analyzed by fluorescence microscopy. (D) The mitochondrial network structures were quantified using ImageJ and the mitochondrial area per perimeter, a measure for their interconnectivity, was calculated. n=4. (E) Cellular mitochondrial mass was determined in ATR-iKO pMEFs by flow cytometry using Mitotracker Deep Red. Error bars show SEM. The statistical analysis was performed using two-tailed *t* tests. *P*-values are indicated within individual graphs. n=4. (F) Western blot analysis of the indicated proteins in HCT116 cells using RNAi with scrambled (siCtl), targeted siRNA against PINK1 (siPINK1#1, #2 or mixture of both (siPINK1 mix)). Vinculin was used to control protein loading. The protein level was quantified by using ImageJ (lower panel). Error bars show SEM. The statistical analysis was performed using two-tailed unpaired *t*-test. Due to their efficiency of knockdown, siPINK#1 or siPINK1 (mix) were used for all following experiments. n=3. (G) TEM analysis of the mitochondrial morphology in ATR-KD and PINK1-KD HCT116 cells. Green arrows point to malformed and swollen mitochondria. Cell organelles are labeled by colored text. The experiment repeated twice.

Figure S2-Marx et al.

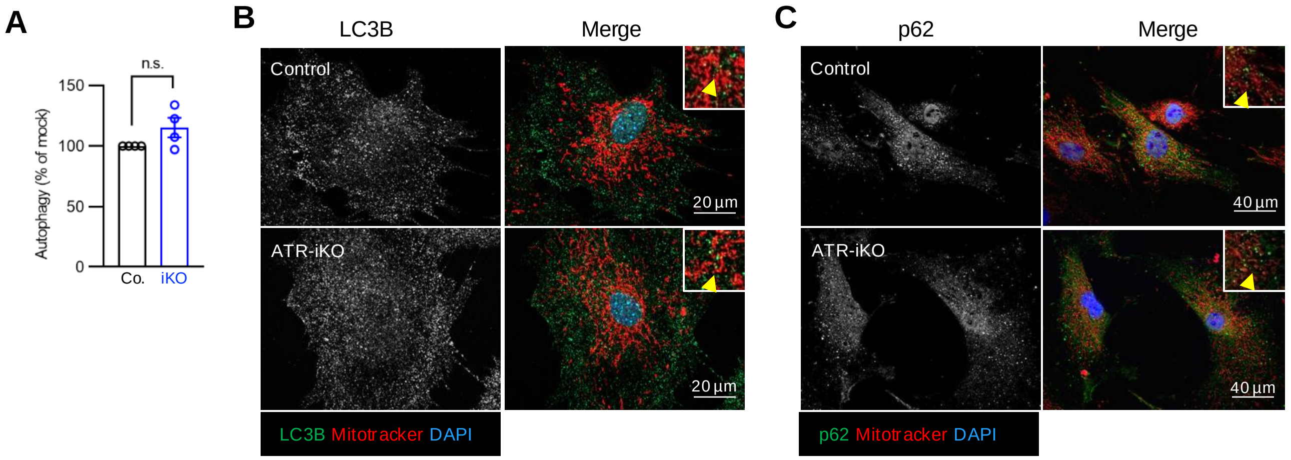

Supplementary figure S3: ATR deletion blunts autophagy initiation. (A) Cellular autophagy of ATR-iKO pMEFs was determined in by flow cytometry using an autophagy detection kit. n=4. Error bars show SEM. The statistical analysis was performed using two-tailed unpaired *t* test. n.s.: not significant. (B-C) Immunofluorescence microscopy of mitochondrial networks (Mitotracker staining) together with (B) anti-LC3B antibody or (C) anti-p62 antibody in ATR-iKO pMEFs and wildtype controls. The insets, in the upper right corners of the merged pictures show a high magnification of LC3B or p62 staining within the mitochondrial networks. n=3.

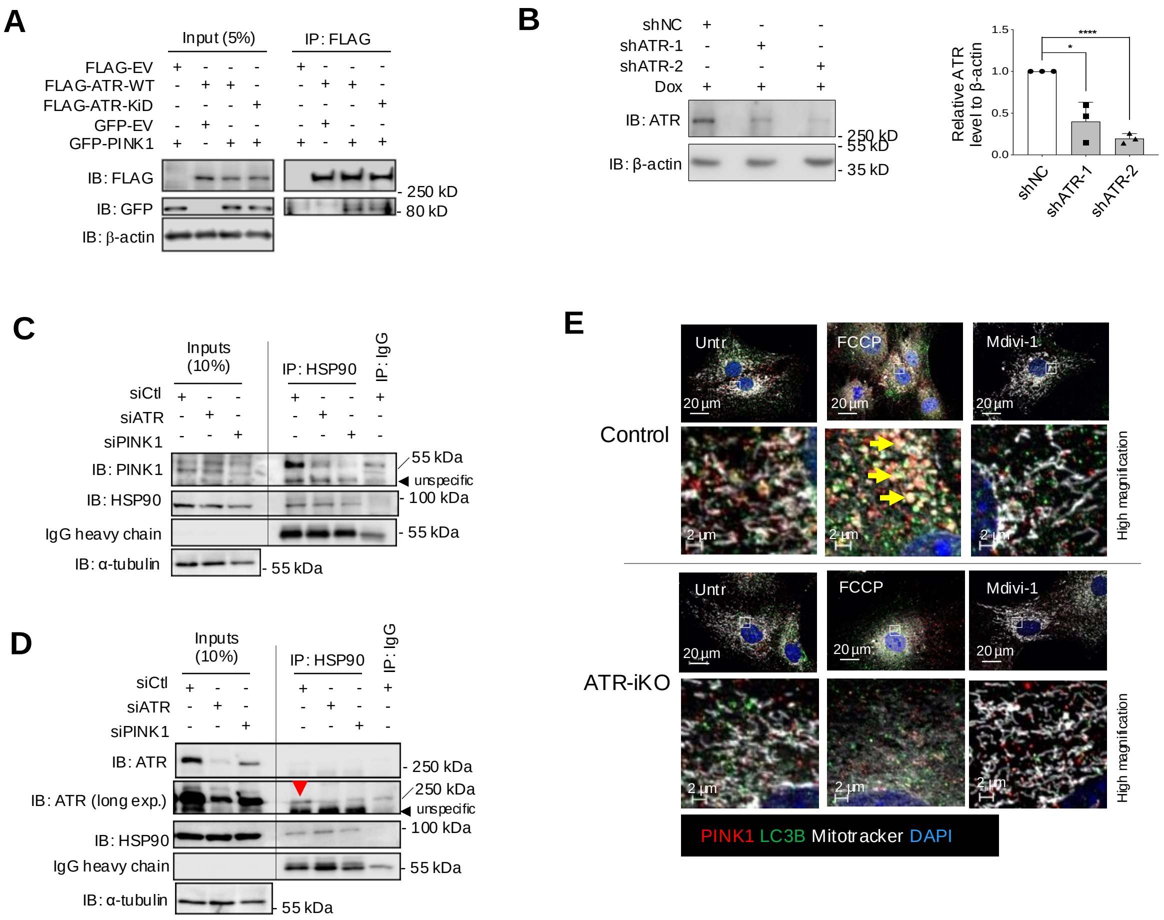

Supplementary figure S4: ATR interacts with mitochondrial proteins. (A) Western blot analysis of HEK 293T cells after transfection with Flag-ATR wildtype (ATR-WT), -kinase dead (ATR-KiD) and GFP-PINK1 constructs with IPs against FLAG (ATR) and blotted by FLAG or GFP. β-actin is a loading control for Input. n=3. (B) Representative Western blot images of ATR knockdown by shATR-1 and shATR-2 after Doxycycline (Dox) treatment (5µg/mL for 48h). shNC is a scrambled control. β-actin was used to control protein loading. Quantification of three Western blot results is performed using ImageJ (lower panel). n=3. Error bars show SEM. The statistical analysis was performed using two-tailed unpaired *t*-test. *, $p<0.05$, ****, $p<0.0001$. (C-D) Co-Immunoprecipitations (Co-IPs) of HSP90α in WCEs of ATR-KD (siATR) and PINK1-KD (siPINK1) HCT116 cells followed by Western blot analysis of the indicated proteins. Scrambled (siCtl) serves a siRNA control. α-tubulin was used to control protein loading. Red arrows indicate specific protein bands. n=3. (E) ATR-iKO pMEFs were treated with 2 µM of the mitochondrial uncoupler FCCP or 5 µM of the Drp1 inhibitor Mdivi-1. Expression and co-localization of PINK1 (pink) and LC3B (green) were analyzed by fluorescence microscopy of immune-labeled proteins at 5 dpo. Mitochondrial networks are visualized by Mitotracker (white) staining. DAPI (blue) stains DNA. Yellow arrows point co-localization of PINK1 and LC3B at mitochondria. n=3.

Figure S4-Marx et al.

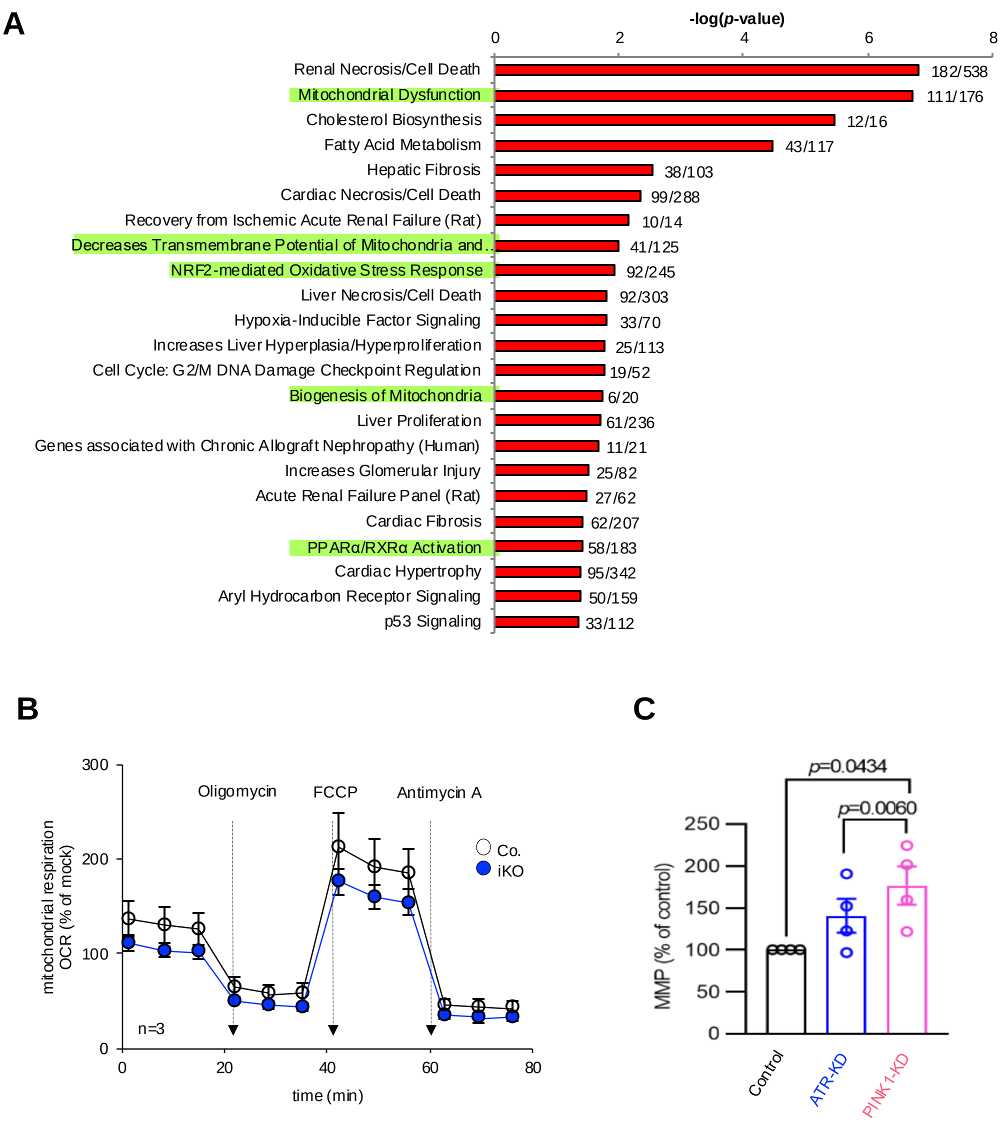

Supplementary figure S5: Mitochondrial metabolic defects in ATR deleted cells. (A) Proteome alterations in ATR-iKO pMEFs were subjected to IPA software (cutoff: q <0.05). Cellular dysfunctions are represented as sorted list according to their p-value (cutoff: -log(p-value) >1.3). Changes of mitochondrial functions are highlighted in green. Three ATR-wildtype and three ATR-iKO cell lines were analysed. (B) Analysis of mitochondrial respiration of ATR-iKO pMEF at 5 dpo using a Seahorse Analyzer. Mitochondrial respiration was modulated by consecutive injections of oligomycin (ETCC-V inhibitor), FCCP and antimycin A (ETCC-III inhibitor). n=3. (C) The mitochondrial membrane potential (MMP) in ATR-KD and PINK1-KD HCT116 cells was analyzed by flow cytometry analysis of DiOC6(3). n=4. Error bars show SEM. The statistical analysis was performed using two-tailed t test. P-values are indicated within individual graphs. n.s., not significant.

Figure S5-Marx et al.

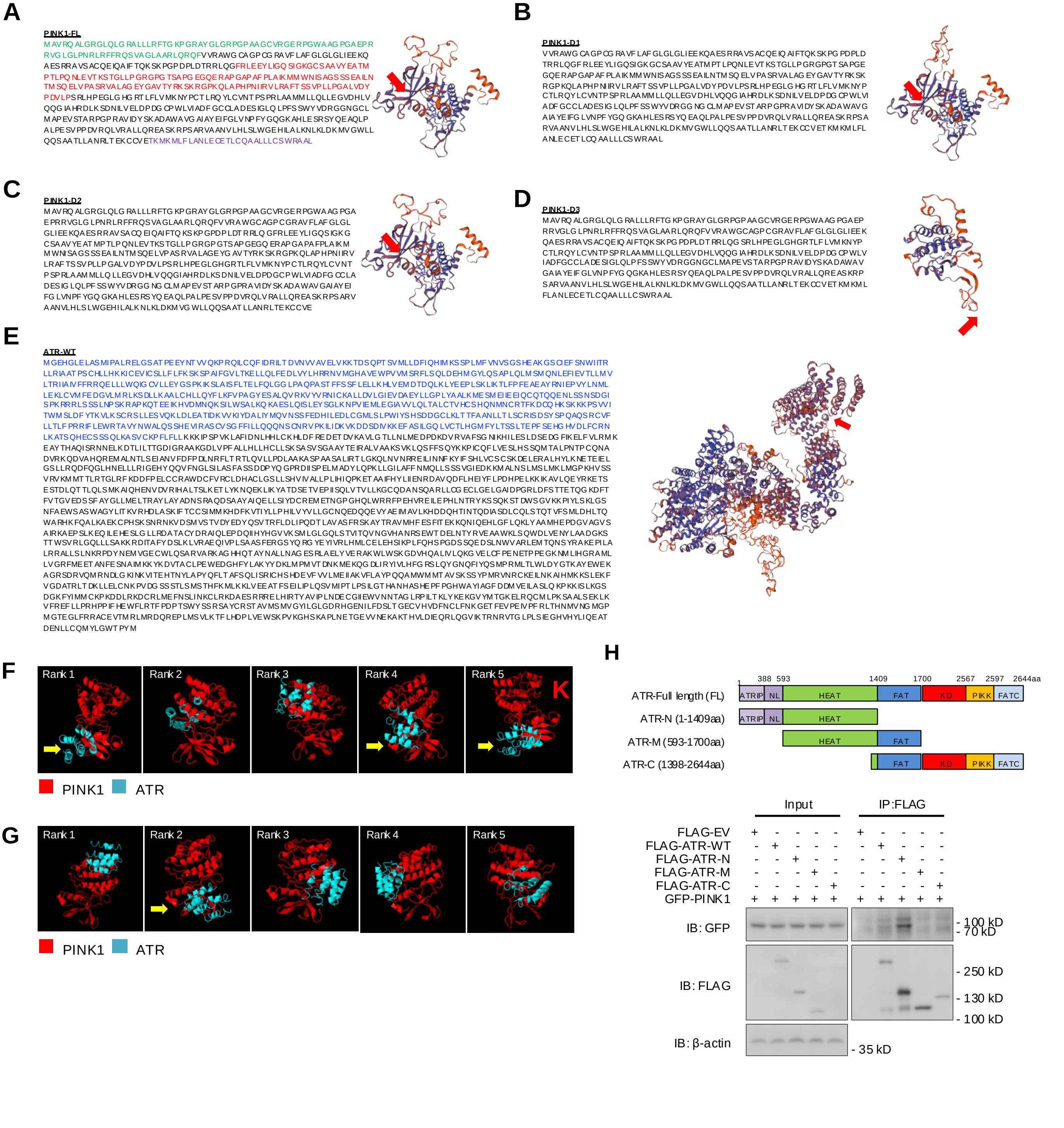

Supplementary figure S6: Modeling of ATR-PINK1 interaction. (A) The 3D structures of human PINK1 protein constructed using the SWISS-MODEL workspace based on their amino acid sequences. The green letters indicate the amino acids deleted in PINK1-D1; the purple deleted in PINK1-D2 and the red deleted in PINK1-D3. (B-D) The predicted 3D protein structure based on the amino acid sequence of human PINK-D1 (B), -D2 (C), -D3 (D), respectively. Red arrows point to the large β-sheet domain of PINK1. (E) The 3D structure of human ATR protein was constructed based on its amino acid sequence. The blue letters (left panel) indicate the N-terminal amino acids used to predict its interaction with PINK1-full length (FL) and PINK1-D3. Red arrow in the right panel points to the N-terminus of ATR, the interaction surface for its partner ATRIP. (F-G) Potential protein-protein interactions between the N-terminus of ATR (turquoise) with (F) PINK1-FL or (G) PINK1-D3 mutant (both red) were predicted using the SPRING server. The top 5 ranked interaction models referring to the SPRING Score are shown. Yellow arrows point the position of ATR binding to the β-sheet domain of PINK1. (H) Left panel: Schematic view of ATR full length (FL) and truncation mutants ATR-N, ATR-M and ATR-C. Functional domains are indicated and the amino acids (aa) at the border of the indicated domains are marked on the top of ATR-FL. Right panel: Co-IPs of HEK239T cells after transfection with FLAG-tagged ATR-FL and its truncation mutants together with GFP-PINK1 followed by Western blotting using the indicated antibodies. β-actin was used to control protein loading in Input. n=3.

A

gene symbol	mean read count	log2 fold change	adjusted p-value	Ensembl ID
ND1	20231.8	0.18292	0.68646	ENSMUSG00000064341
ND2	7325.7	0.10503	0.86970	ENSMUSG00000064345
ND4	3936.2	0.07163	0.91795	ENSMUSG00000064363
ND5	7980.9	0.05115	0.94029	ENSMUSG00000064367
ND6	340.3	-0.07117	0.91969	ENSMUSG00000064368
COX1	44984.6	0.16884	0.73465	ENSMUSG00000064351
CYTB	19154.5	0.11133	0.85841	ENSMUSG00000064370

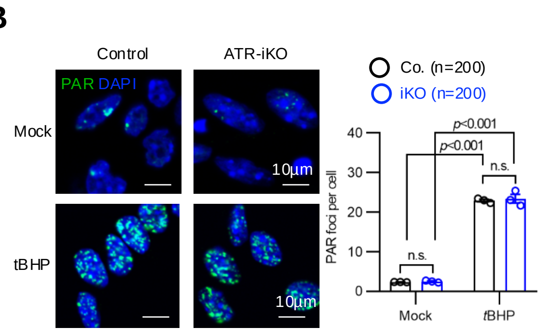

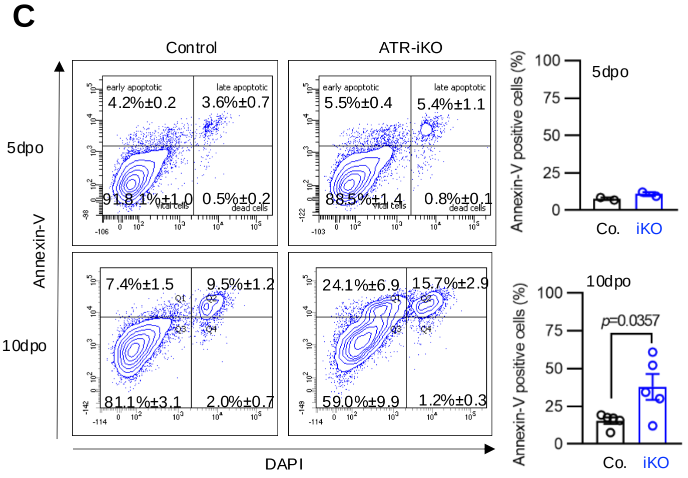

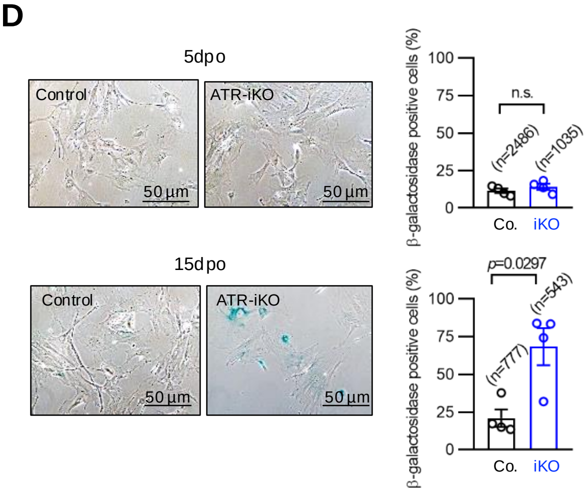

Supplementary figure S7: ATR deficient cells accumulate nDNA damages. (A) RNA-seq analysis of mitochondria-encoded genes in control and ATR-iKO MEFs conducted with STAR, FeatureCounts and DESeq2. Only genes with non-zero mean read count are considered. Three ATR-wildtype and three ATR-iKO cell lines were analysed. (B) ATR-iKO pMEFs were treated with *t*-butyl hydrogenperoxide (tBHP) to induce oxidative stress. Formation of poly(ADP-ribose) (PAR) foci was analyzed by fluorescence microscopy of immune-labeled PAR in 200 randomly chosen cells per condition. DAPI stains DNA. Quantification of PAR foci per cell was done manually. The number of cells (n) of each genotype is indicated. (C) The amount of early (Annexin-V+ / DAPI-) and late apoptotic (Annexin-V+ / DAPI+) as well as necrotic cells (Annexin-V- / DAPI+) was analyzed by flow cytometry at 5 dpo and 10 dpo in ATR-iKO pMEFs. The right panels show the total number of Annexin-V positive cells at both time points. n=2 for 5 dpo and n=5 for 10 dpo. (D) The β-galactosidase staining of ATR-iKO pMEFs and controls was analyzed by light microscopy at 5 dpo and 15 dpo, respectively. The percentage of β-galactosidase positive cells was scored manually by counting of the microscopy pictures. n=4. Error bars show SEM. The statistical analysis was performed using two-tailed unpaired *t*-test. *P*-values are indicated within individual graphs. n.s., not significant.

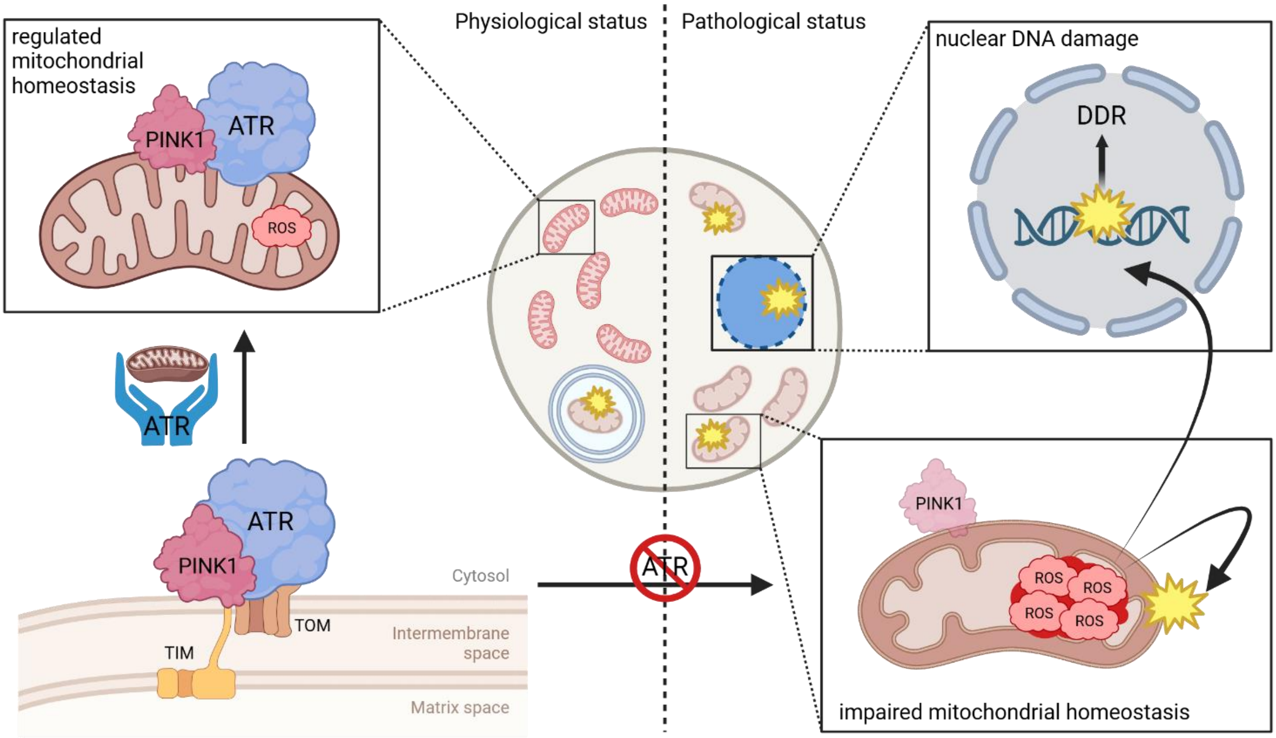

Supplementary figure S8: Working model on ATR's role in mitochondrial homeostasis and nuclear DDR. ATR and PINK1 dock at the TOM/TIM complex in mitochondria. ATR, via its scaffold, stabilises PINK1 at mitochondria, which is essential for the mitochondrial quality control (MQC) program, namely mitophagy, thereby maintaining mitochondrial homeostasis under the physiological condition. In the absence of ATR or loss its interaction, PINK1 fails to execute mitophagy, resulting in dysfunctional mitochondria metabolism and overproduction of reactive oxygen species (ROS). High ROS initially cause mitochondrial (DNA) damage, protein oxidation and subsequently nuclear DNA damage. Thus, ATR, participating in PINK1-mediated mitophagy, ensures mitochondrial quality and functionality in the physiological status, which also prevents subsequent nuclear DNA damage and other deleterious effect to cells, thereby shielding tissues from pathogenesis.
