# Supplementary material for: DNA damage response regulator ATR licenses PINK1-mediated mitophagy
Source: Nucleic Acids Res. 2025 Mar 19;53(5):gkaf178. doi: 10.1093/nar/gkaf178 (PMC11920799; doi:10.1093/nar/gkaf178)
Supplement: gkaf178_Supplemental_Files [file gkaf178_supplemental_files.zip › Suppl. file1-new-s.pdf]

Symbol	Entrez Gene Name	GenPept/UniProt/Swiss-Prot Accession(A2) Atr-iKO	GenPept/UniProt/Swiss-Prot Accession(A2) Control	Expr Log Ratio(A2) Atr-iKO	Expr Log Ratio(A2) Control	Expr False Discovery Rate (q-value)(A2) Atr-iKO	Expr False Discovery Rate (q-value)(A2) Control
ARFGEF2	ADP ribosylation factor guanine nucleotide exchange factor 2	A2A5R2	A2A5R2	-1,339	-0,005	9,48E-02	4,04E-01
Hist1h1a	histone cluster 1, H1a	P43275	--	-1,279		7,43E-03	
Scd2	stearoyl-Coenzyme A desaturase 2	P13011	--	-1,268		1,10E-02	
COG7	component of oligomeric golgi complex 7	Q3UM29	--	-1,184		3,44E-02	
**ATP5B**	**ATP synthase, H+ transporting, mitochondrial F1 complex, beta polypeptide**	P56480	--	-1,169		3,03E-05	
**TXNRD2**	**thioredoxin reductase 2**	Q9JLT4	--	-1,123		9,24E-02	
GADD45GI	GADD45G interacting protein 1	Q9CR59	Q9CR59	-1,106	-0,393	3,77E-01	4,24E-01
IL1RN	interleukin 1 receptor antagonist	P25085	--	-1,055		1,10E-03	
SLC35E1	solute carrier family 35 member E1	Q8CD26	--	-1,004		4,28E-02	
FNBP1	formin binding protein 1	Q80TY0	--	-0,999		4,19E-01	
**DNPH1**	**2'-deoxynucleoside 5'-phosphate N-hydrolase 1**	Q80VJ3	--	-0,989		1,67E-01	
**Rpl22l1**	**ribosomal protein L22 like 1**	Q9D7S7	--	-0,971		4,74E-03	
GXYLT1	glucoside xylosyltransferase 1	Q3UHH8	--	-0,97		2,00E-01	
SIRPA	signal regulatory protein alpha	P97797	--	-0,861		4,15E-02	
**MYH7**	**myosin heavy chain 7**	Q91Z83	--	-0,855		2,90E-02	
**LAMA2**	**laminin subunit alpha 2**	Q60675	--	-0,836		3,32E-01	
**TCEA1**	**transcription elongation factor A1**	P10711	P10711	-0,82	-0,174	2,39E-05	8,53E-03
KDELR1	KDEL endoplasmic reticulum protein retention receptor 1	Q99JH8	--	-0,819		1,97E-01	
MCM3	minichromosome maintenance complex component 3	P25206	--	-0,801		1,08E-02	
VCAN	versican	Q62059	Q62059	-0,782	-0,182	5,24E-04	5,47E-01
NUDT16L1	nudix hydrolase 16 like 1	Q8VHN8	--	-0,776		2,34E-01	
HMGB2	high mobility group box 2	P30681	--	-0,76		7,39E-02	
CFDP1	craniofacial development protein 1	O88271	--	-0,74		7,24E-03	
DNMT1	DNA methyltransferase 1	P13864	--	-0,734		2,52E-02	
**PGP**	**phosphoglycolate phosphatase**	Q8CHP8	--	-0,717		4,92E-02	
**COX6A1**	**cytochrome c oxidase subunit 6A1**	P43024	--	-0,716		2,05E-03	
SMARCC1	SWI/SNF related, matrix associated, actin dependent regulator of chromatin subfamily c member 1	P97496	--	-0,697		4,68E-02	
CXCL12	C-X-C motif chemokine ligand 12	P40224	--	-0,648		3,86E-01	
**MRPS14**	**mitochondrial ribosomal protein S14**	Q9CR88	--	-0,633		3,72E-02	
COL6A1	collagen type VI alpha 1 chain	Q04857	Q04857	-0,631	-0,274	1,41E-02	1,69E-03

Z-score

-2 -1 0 1 2

FDFT1	farnesyl-diphosphate farnesyltransferase 1	P53798	P53798	-0,63	0,247	4,92E-02	5,63E-01
BCAT1	branched chain amino acid transaminase 1	P24288	P24288	-0,615	-0,157	5,17E-02	1,05E-02
SMC3	structural maintenance of chromosomes 3	Q9CW03	--	-0,612		5,29E-04	
YTHDF1	YTH N6-methyladenosine RNA binding protein 1	P59326	--	-0,605		1,02E-02	
TSTA3	tissue specific transplantation antigen P35B	P23591	--	-0,604		3,20E-01	
**RABL6**	**RAB, member RAS oncogene family like 6**	Q5U3K5	Q5U3K5	-0,586	-0,126	2,99E-01	3,83E-01
UBQLN1	ubiquilin 1	Q8R317	--	-0,576		2,62E-02	
Hist1h1b	histone cluster 1, H1b	P43276	P43276	-0,57	-0,15	4,52E-07	3,41E-02
Gm21596/	high mobility group box 1	P63158	P63158	-0,551	-0,32	2,70E-03	1,22E-03
MCM2	minichromosome maintenance complex component 2	P97310	P97310	-0,55	-0,014	1,49E-05	1,13E-02
**HMGCS1**	**3-hydroxy-3-methylglutaryl-CoA synthase 1**	Q8JZK9	Q8JZK9	-0,542	-0,359	8,80E-07	2,09E-04
AQR	aquarius intron-binding spliceosomal factor	Q8CFQ3	--	-0,541		1,71E-02	
CDK1	cyclin dependent kinase 1	P11440	--	-0,541		1,64E-04	
MCM7	minichromosome maintenance complex component 7	Q61881	--	-0,541		3,85E-02	
KIF23	kinesin family member 23	E9Q5G3	--	-0,531		3,59E-02	
Hmgb3	high mobility group box 3	O54879	--	-0,508		2,14E-02	
Rbmxl1	RNA binding motif protein, X-linked like-1	Q91VM5	--	-0,494		8,81E-02	
FDPS	farnesyl diphosphate synthase	Q920E5	Q920E5	-0,481	-0,293	3,49E-04	1,19E-03
MCM5	minichromosome maintenance complex component 5	P49718	--	-0,465		4,81E-05	
VPS26B	VPS26, retromer complex component B	Q8C0E2	--	-0,465		5,87E-01	
H2AFY2	H2A histone family member Y2	Q8CCK0	--	-0,461		3,34E-05	
**PARP1**	**poly(ADP-ribose) polymerase 1**	P11103	--	-0,458		4,98E-05	
MRFAP1	Morf4 family associated protein 1	Q9CQL7	--	-0,456		1,75E-02	
HDAC1	histone deacetylase 1	O09106	--	-0,454		3,43E-01	
SAFB2	scaffold attachment factor B2	Q80YR5	--	-0,454		3,93E-03	
MCM6	minichromosome maintenance complex component 6	P97311	--	-0,453		2,02E-06	
FBLN5	fibulin 5	Q9WVH9	--	-0,451		1,18E-02	
ETV6	ETS variant 6	P97360	--	-0,447		9,51E-02	
CLUH	clustered mitochondria homolog	Q5SW19	--	-0,446		1,32E-02	
CTHRC1	collagen triple helix repeat containing 1	Q9D1D6	Q9D1D6	-0,444	-0,184	6,65E-05	6,95E-03
HIST1H1C	histone cluster 1 H1 family member c	P15864	--	-0,442		6,32E-04	

PIGN	phosphatidylinositol glycan anchor biosynthesis class N	Q9R1S3	--	-0,441		1,66E-03	
LCP1	lymphocyte cytosolic protein 1	Q61233	--	-0,441		7,99E-02	
COL11A1	collagen type XI alpha 1 chain	Q61245	Q61245	-0,439	-0,199	1,93E-04	3,79E-02
PUM3	pumilio RNA binding family member 3	Q8BKS9	--	-0,436		1,37E-02	
DEK	DEK proto-oncogene	Q7TNV0	--	-0,432		7,06E-03	
CMAS	cytidine monophosphate N-acetylneuraminic acid synthetase	Q99KK2	--	-0,431		7,02E-03	
Hist1h1e	histone cluster 1, H1e	P43274	--	-0,428		4,19E-02	
**RPL39**	**ribosomal protein L39**	P62892	--	-0,424		2,89E-02	
PDS5B	PDS5 cohesin associated factor B	Q4VA53	--	-0,423		6,43E-03	
FAM160B1	family with sequence similarity 160 member B1	Q8CDM8	Q8CDM8	-0,421	-1,11	9,34E-03	5,17E-01
PSAT1	phosphoserine aminotransferase 1	Q99K85	Q99K85	-0,41	-0,156	7,91E-03	1,70E-03
CARHSP1	calcium regulated heat stable protein 1	Q9CR86	--	-0,407		5,99E-02	
**POLR3F**	**RNA polymerase III subunit F**	Q921X6	--	-0,404		7,87E-03	
NUTF2	nuclear transport factor 2	P61971	--	-0,4		8,20E-02	
NAA15	N(alpha)-acetyltransferase 15, NatA auxiliary subunit	Q80UM3	--	-0,399		1,65E-05	
C12orf10	chromosome 12 open reading frame 10	Q9JK81	--	-0,398		1,82E-03	
RND3	Rho family GTPase 3	P61588	--	-0,395		3,17E-02	
XPOT	exportin for tRNA	Q9CRT8	--	-0,395		2,13E-02	
**RPS7**	**ribosomal protein S7**	P62082	P62082	-0,394	-0,446	4,82E-02	3,54E-02
IKBKG	inhibitor of nuclear factor kappa B kinase subunit gamma	O88522	--	-0,393		4,28E-01	
RPA1	replication protein A1	Q8VEE4	--	-0,39		2,37E-02	
**HSPBP1**	**HSPA (Hsp70) binding protein 1**	Q99P31	--	-0,39		1,31E-02	
PIK3R1	phosphoinositide-3-kinase regulatory subunit 1	P26450	P26450	-0,389	-0,885	1,91E-01	2,98E-01
GATAD2A	GATA zinc finger domain containing 2A	Q8CHY6	--	-0,38		4,05E-02	
SMU1	DNA replication regulator and spliceosomal factor	Q3UKJ7	--	-0,38		9,79E-02	
**IGF2**	**insulin like growth factor 2**	P09535	--	-0,377		3,60E-01	
BTF3	basic transcription factor 3	Q64152	--	-0,376		8,27E-03	
CSE1L	chromosome segregation 1 like	Q9ERK4	--	-0,376		5,06E-04	
GSPT1	G1 to S phase transition 1	Q8R050	--	-0,375		2,26E-02	
EXOSC4	exosome component 4	Q921I9	--	-0,375		3,43E-02	
GARS	glycyl-tRNA synthetase	Q9CZD3	--	-0,374		1,58E-03	
GNAI1	G protein subunit alpha i1	B2RSH2	--	-0,371		1,07E-02	
NCBP1	nuclear cap binding protein subunit 1	Q3UYV9	--	-0,371		1,20E-03	
NOL10	nucleolar protein 10	Q5RJG1	--	-0,37		6,39E-02	
**TOP2A**	**topoisomerase (DNA) II alpha**	Q01320	--	-0,369		6,44E-04	

COL3A1	collagen type III alpha 1 chain	P08121	P08121	-0,369	-0,347	5,42E-21	1,03E-26
**FADS2**	**fatty acid desaturase 2**	Q9Z0R9	--	-0,365		2,88E-04	
YBX3	Y-box binding protein 3	Q9JKB3	--	-0,365		5,96E-06	
LSM4	LSM4 homolog, U6 small nuclear RNA and mRNA degradation associated	Q9QXA5	--	-0,363		1,03E-02	
SRSF2	serine and arginine rich splicing factor 2	Q62093	--	-0,361		1,55E-03	
**RPL3**	**ribosomal protein L3**	P27659	P27659	-0,36	-0,228	2,94E-01	1,73E-03
**RAD21**	**RAD21 cohesin complex component**	Q61550	--	-0,355		3,12E-02	
**Rpl32**	**ribosomal protein L32**	P62911	P62911	-0,355	-0,185	2,51E-02	6,97E-03
**RPL5**	**ribosomal protein L5**	P47962	--	-0,354		9,27E-02	
DHX15	DEAH-box helicase 15	O35286	--	-0,35		1,11E-02	
**PLA2G4A**	**phospholipase A2 group IVA**	P47713	--	-0,35		1,40E-02	
**RANBP3**	**RAN binding protein 3**	Q9CT10	--	-0,347		5,58E-04	
FAU	FAU, ubiquitin like and ribosomal protein S30 fusion	P62862	--	-0,347		1,51E-02	
MED24	mediator complex subunit 24	Q99K74	--	-0,345		7,97E-02	
EEF2	eukaryotic translation elongation factor 2	P58252	P58252	-0,344	-0,084	9,88E-02	1,34E-03
MAP2K4	mitogen-activated protein kinase kinase 4	P47809	--	-0,342		4,87E-01	
NOL11	nucleolar protein 11	Q8BJW5	--	-0,341		4,27E-02	
RBM3	RNA binding motif (RNP1, RRM) protein 3	O89086	O89086	-0,341	-0,199	2,55E-02	3,91E-02
KIF1B	kinesin family member 1B	Q60575	--	-0,34		2,34E-03	
RBM10	RNA binding motif protein 10	Q99KG3	--	-0,337		3,97E-02	
IDI1	isopentenyl-diphosphate delta isomerase 1	P58044	--	-0,329		2,07E-03	
YARS2	tyrosyl-tRNA synthetase 2	Q8BYL4	--	-0,325		4,96E-02	
SMC2	structural maintenance of chromosomes 2	Q8CG48	--	-0,323		2,04E-02	
**RPS8**	**ribosomal protein S8**	P62242	--	-0,322		3,56E-02	
WBP11	WW domain binding protein 11	Q923D5	--	-0,319		7,66E-04	
Rps3a1	ribosomal protein S3A1	P97351	--	-0,318		3,74E-01	
XPO1	exportin 1	Q6P5F9	Q6P5F9	-0,316	-0,231	1,73E-04	4,90E-02
UBE2K	ubiquitin conjugating enzyme E2 K	P61087	--	-0,316		7,56E-02	
PSIP1	PC4 and SFRS1 interacting protein 1	Q99JF8	--	-0,316		2,14E-05	
PPIL1	peptidylprolyl isomerase like 1	Q9D0W5	--	-0,315		6,96E-02	
GRB2	growth factor receptor bound protein 2	Q60631	--	-0,314		2,25E-02	
Chtop	chromatin target of PRMT1	Q9CY57	--	-0,314		3,55E-02	
DDX39B	DExD-box helicase 39B	Q9Z1N5	Q9Z1N5	-0,311	-0,289	1,74E-02	2,96E-02
**RPL13A**	**ribosomal protein L13a**	P19253	--	-0,311		4,95E-05	
**RACK1**	**receptor for activated C kinase 1**	P68040	--	-0,31		6,30E-02	
**POLR1D**	**RNA polymerase I subunit D**	P97304	--	-0,309		4,06E-02	
TSR1	TSR1, ribosome maturation factor	Q5SWD9	Q5SWD9	-0,307	-0,267	2,26E-04	2,21E-02

NSA2	NSA2, ribosome biogenesis homolog	Q9CR47	--	-0,302		1,28E-02	
TPBG	trophoblast glycoprotein	Q9Z0L0	--	-0,3		5,73E-03	
Otub1	OTU domain, ubiquitin aldehyde binding 1	Q7TQI3	--	-0,296		2,65E-02	
CMTM3	CKLF like MARVEL transmembrane domain containing 3	Q99LJ5	--	-0,294		5,85E-01	
INTS3	integrator complex subunit 3	Q7TPD0	--	-0,293		2,69E-02	
PUM1	pumilio RNA binding family member 1	Q80U78	--	-0,292		1,00E-02	
WDR3	WD repeat domain 3	Q8BHB4	--	-0,292		7,85E-02	
IPO4	importin 4	Q8VI75	--	-0,29		6,77E-02	
CTBP2	C-terminal binding protein 2	P56546	--	-0,29		1,53E-02	
NXF1	nuclear RNA export factor 1	Q99JX7	--	-0,289		3,47E-02	
LAP3	leucine aminopeptidase 3	Q9CPY7	--	-0,287		3,01E-04	
CWC15	CWC15 spliceosome associated protein homolog	Q9JHS9	--	-0,287		4,16E-03	
CBX3	chromobox 3	P23198	P23198	-0,286	-0,153	6,64E-05	3,33E-02
**EIF2S3**	**eukaryotic translation initiation factor 2 subunit gamma**	Q9Z0N1	Q9Z0N1	-0,286	0,115	2,66E-01	1,82E-03
COG6	component of oligomeric golgi complex 6	Q8R3I3	--	-0,285		3,25E-02	
PAFAH1B3	platelet activating factor acetylhydrolase 1b catalytic subunit 3	Q61205	--	-0,284		2,05E-01	
BABAM1	BRISC and BRCA1 A complex member 1	Q3UI43	--	-0,282		3,43E-02	
Alyref	Aly/REF export factor	O08583	--	-0,279		1,56E-02	
**RICTOR**	**RPTOR independent companion of MTOR complex 2**	Q6QI06	Q6QI06	-0,279	0,977	1,61E-01	5,02E-01
ANXA6	annexin A6	P14824	P14824	-0,275	-0,285	4,40E-02	3,18E-03
GNL1	G protein nucleolar 1 (putative)	P36916	--	-0,275		2,40E-01	
PCID2	PCI domain containing 2	Q8BFV2	--	-0,275		2,00E-01	
PRPF31	pre-mRNA processing factor 31	Q8CCF0	--	-0,274		1,13E-02	
ITGB3	integrin subunit beta 3	O54890	O54890	-0,271	-0,203	1,49E-01	2,55E-01
IL6ST	interleukin 6 signal transducer	Q00560	--	-0,27		1,41E-02	
MRTO4	MRT4 homolog, ribosome maturation factor	Q9D0I8	--	-0,269		1,59E-03	
**RPL19**	**ribosomal protein L19**	P84099	--	-0,268		7,37E-04	
**RPS13**	**ribosomal protein S13**	P62301	P62301	-0,267	-0,093	6,69E-03	1,82E-02
CHORDC1	cysteine and histidine rich domain containing 1	Q9D1P4	--	-0,266		2,19E-02	
NASP	nuclear autoantigenic sperm protein	Q99MD9	--	-0,264		2,03E-04	
YTHDF2	YTH N6-methyladenosine RNA binding protein 2	Q91YT7	--	-0,264		7,58E-04	
PPIA	peptidylprolyl isomerase A	P17742	--	-0,264		1,69E-01	

SEC13	SEC13 homolog, nuclear pore and COPII coat complex component	Q9D1M0	--	-0,264		1,49E-01	
**RPS20**	**ribosomal protein S20**	P60867	--	-0,264		6,02E-01	
**RPS19**	**ribosomal protein S19**	Q9CZX8	--	-0,261		4,98E-03	
JPT2	Jupiter microtubule associated homolog 2	Q6PGH2	--	-0,26		1,33E-02	
SF3B5	splicing factor 3b subunit 5	Q923D4	--	-0,26		2,22E-02	
NOL9	nucleolar protein 9	Q3TZX8	--	-0,26		2,10E-02	
**MICU1**	**mitochondrial calcium uptake 1**	Q8VCX5	--	-0,259		1,58E-01	
**IDH1**	**isocitrate dehydrogenase (NADP(+)) 1, cytosolic**	O88844	O88844	-0,259	-0,012	2,47E-02	5,06E-01
BLMH	bleomycin hydrolase	Q8R016	--	-0,259		9,97E-02	
UTP6	UTP6, small subunit processome component	Q8VCY6	--	-0,256		3,12E-02	
NIP7	NIP7, nucleolar pre-rRNA processing protein	Q9CXK8	--	-0,255		3,93E-02	
**EIF5A**	**eukaryotic translation initiation factor 5A**	P63242	--	-0,254		5,46E-03	
UBE2I	ubiquitin conjugating enzyme E2 I	P63280	--	-0,253		2,57E-02	
UBAP2	ubiquitin associated protein 2	Q91VX2	--	-0,253		8,22E-04	
SGPP1	sphingosine-1-phosphate phosphatase 1	Q9JI99	Q9JI99	-0,253	0,056	3,47E-01	4,50E-01
NCEH1	neutral cholesterol ester hydrolase 1	Q8BLF1	--	-0,252		2,32E-01	
PPA1	pyrophosphatase (inorganic) 1	Q9D819	--	-0,252		1,05E-02	
LDLR	low density lipoprotein receptor	P35951	P35951	-0,252	-0,037	1,92E-01	3,54E-01
**IDH2**	**isocitrate dehydrogenase (NADP(+)) 2, mitochondrial**	P54071	P54071	-0,251	0,055	4,52E-05	4,22E-02
SUPT5H	SPT5 homolog, DSIF elongation factor subunit	O55201	--	-0,25		4,93E-02	
UBTF	upstream binding transcription factor, RNA polymerase I	P25976	--	-0,25		2,23E-01	
TEX10	testis expressed 10	Q3URQ0	--	-0,25		2,54E-02	
SLC16A3	solute carrier family 16 member 3	P57787	P57787	-0,25	-0,155	3,28E-04	3,47E-03
AHCY	adenosylhomocysteinase	P50247	--	-0,249		2,57E-03	
PAFAH1B2	platelet activating factor acetylhydrolase 1b catalytic subunit 2	Q61206	--	-0,248		8,68E-02	
FUBP1	far upstream element binding protein 1	Q91WJ8	Q91WJ8	-0,247	-0,102	1,95E-07	1,12E-02
DDX54	DEAD-box helicase 54	Q8K4L0	--	-0,247		2,78E-04	
TCOF1	treacle ribosome biogenesis factor 1	O08784	O08784	-0,246	-0,066	2,98E-03	4,43E-01
EBNA1BP2	EBNA1 binding protein 2	Q9D903	--	-0,246		5,86E-01	
PSMD6	proteasome 26S subunit, non-ATPase 6	Q99JI4	Q99JI4	-0,245	-0,171	6,53E-04	1,98E-02
HYPK	huntingtin interacting protein K	Q9CR41	--	-0,245		1,33E-01	

MTHFD2	methylenetetrahydrofolate dehydrogenase (NADP+ dependent) 2, methenyltetrahydrofolate	P18155	--	-0,243		3,06E-04	
**RPS2**	**ribosomal protein S2**	P25444	P25444	-0,243	-0,11	9,30E-02	1,85E-03
**RPL27**	**ribosomal protein L27**	P61358	P61358	-0,24	-0,114	9,85E-02	7,50E-04
HDAC2	histone deacetylase 2	P70288	P70288	-0,238	-0,059	5,11E-07	4,48E-02
RUVBL2	RuvB like AAA ATPase 2	Q9WTM5	--	-0,238		5,43E-01	
**RPL7**	**ribosomal protein L7**	P14148	--	-0,238		6,66E-03	
SUPT16H	SPT16 homolog, facilitates chromatin remodeling subunit	Q920B9	--	-0,236		5,67E-04	
**PCNA**	**proliferating cell nuclear antigen**	P17918	--	-0,235		3,45E-03	
QSOX2	quiescin sulfhydryl oxidase 2	Q3TMX7	--	-0,235		3,42E-02	
**RPL35A**	**ribosomal protein L35a**	O55142	--	-0,234		5,36E-03	
DIS3	DIS3 homolog, exosome endoribonuclease and 3'-5' exoribonuclease	Q9CSH3	--	-0,234		1,45E-03	
FKBP4	FK506 binding protein 4	P30416	--	-0,233		2,83E-05	
PPID	peptidylprolyl isomerase D	Q9CR16	--	-0,232		2,56E-02	
**DNAJC2**	**DnaJ heat shock protein family (Hsp40) member C2**	P54103	--	-0,231		7,31E-03	
SENP3	SUMO1/sentrin/SMT3 specific peptidase 3	Q9EP97	--	-0,23		1,46E-02	
DDAH1	dimethylarginine dimethylaminohydrolase 1	Q9CWS0	--	-0,227		2,62E-03	
**MT-ND4**	**NADH dehydrogenase, subunit 4 (complex I)**	P03911	--	-0,226		1,64E-01	
FMR1	fragile X mental retardation 1	P35922	P35922	-0,226	0,062	3,39E-01	4,98E-01
**Eif1**	**eukaryotic translation initiation factor 1**	P48024	--	-0,226		2,36E-02	
SEC31A	SEC31 homolog A, COPII coat complex component	Q3UPL0	Q3UPL0	-0,226	-0,106	1,45E-02	4,78E-03
RCC1	regulator of chromosome condensation 1	Q8VE37	--	-0,226		2,90E-02	
RGPD4 (inc	RANBP2-like and GRIP domain containing 5	Q9ERU9	--	-0,225		1,70E-02	
AGO2	argonaute 2, RISC catalytic component	Q8CJG0	--	-0,225		3,26E-03	
ASNS	asparagine synthetase (glutamine-hydrolyzing)	Q61024	Q61024	-0,224	-0,089	9,94E-03	2,97E-02
EFTUD2	elongation factor Tu GTP binding domain containing 2	O08810	--	-0,223		9,87E-03	
FBLN2	fibulin 2	P37889	P37889	-0,223	0,013	1,19E-03	1,88E-02
**CYP1B1**	**cytochrome P450 family 1 subfamily B member 1**	Q64429	Q64429	-0,223	-0,254	5,29E-02	5,74E-02
FHL3	four and a half LIM domains 3	Q9R059	Q9R059	-0,222	-0,102	6,20E-03	2,32E-02
TNPO1	transportin 1	Q8BFY9	--	-0,221		1,23E-01	
DHX36	DEAH-box helicase 36	Q8VHK9	--	-0,221		4,65E-02	
UTP20	UTP20, small subunit processome component	Q5XG71	--	-0,22		9,43E-02	

SQLE	squalene epoxidase	P52019	--	-0,22		3,20E-02	
EMILIN1	elastin microfibril interfacer 1	Q99K41	--	-0,218		7,11E-05	
RTCB	RNA 2',3'-cyclic phosphate and 5'-OH ligase	Q99LF4	--	-0,218		4,92E-02	
Fus	fused in sarcoma	P56959	P56959	-0,218	-0,071	2,26E-05	6,50E-04
**RPS21**	**ribosomal protein S21**	Q9CQR2	--	-0,218		1,29E-01	
LGALS3	galectin 3	P16110	P16110	-0,218	0,046	1,38E-02	9,01E-02
FKBP3	FK506 binding protein 3	Q62446	--	-0,218		1,29E-02	
TMPO	thymopoietin	Q61033	Q61029	-0,217	-0,15	3,17E-02	1,20E-01
**RPSA**	**ribosomal protein SA**	P14206	P14206	-0,217	0,037	1,76E-04	1,43E-02
KRAS	KRAS proto-oncogene, GTPase	P32883	P32883	-0,216	0,366	3,06E-01	5,35E-01
TRIM28	tripartite motif containing 28	Q62318	--	-0,216		8,97E-04	
**RPS10**	**ribosomal protein S10**	P63325	--	-0,215		3,58E-03	
MBNL1	muscleblind like splicing regulator 1	Q9JKP5	--	-0,214		1,42E-02	
SAMHD1	SAM and HD domain containing deoxynucleoside triphosphate triphosphohydrolase 1	Q60710	--	-0,214		1,47E-03	
SRI	sorcin	Q6P069	--	-0,213		2,57E-01	
THRAP3	thyroid hormone receptor associated protein 3	Q569Z6	--	-0,212		1,04E-04	
**RPL15**	**ribosomal protein L15**	Q9CZM2	--	-0,212		1,34E-02	
PRKG1	protein kinase, cGMP-dependent, type I	P0C605	P0C605	-0,212	-0,083	3,29E-01	3,20E-01
SLTM	SAFB like transcription modulator	Q8CH25	--	-0,211		4,85E-02	
MOCS3	molybdenum cofactor synthesis 3	A2BDX3	--	-0,21		2,36E-02	
**GPI**	**glucose-6-phosphate isomerase**	P06745	--	-0,21		4,36E-04	
MAP2K3	mitogen-activated protein kinase kinase 3	O09110	O09110	-0,209	0,2	2,15E-01	6,08E-01
KHDRBS1	KH RNA binding domain containing, signal transduction associated 1	Q60749	Q60749	-0,208	-0,088	2,19E-02	4,00E-02
WDR82	WD repeat domain 82	Q8BFQ4	--	-0,208		2,95E-02	
NELFB	negative elongation factor complex member B	Q8C4Y3	--	-0,207		2,67E-01	
GATAD2B	GATA zinc finger domain containing 2B	Q8VHR5	--	-0,207		4,29E-02	
NDC1	NDC1 transmembrane nucleoporin	Q8VCB1	--	-0,206		9,65E-03	
UBE2M	ubiquitin conjugating enzyme E2 M	P61082	--	-0,205		1,39E-02	
FBN1	fibrillin 1	Q61554	Q61554	-0,205	-0,276	5,12E-01	4,89E-08
SRSF10	serine and arginine rich splicing factor 10	Q9R0U0	--	-0,203		6,22E-03	
SMARCA5	SWI/SNF related, matrix associated, actin dependent regulator of chromatin, subfamily a, member 5	Q91ZW3	--	-0,202		1,68E-04	
MAP4K4	mitogen-activated protein kinase kinase kinase kinase 4	P97820	P97820	-0,201	-0,022	5,57E-01	3,69E-01

Snrpg	small nuclear ribonucleoprotein polypeptide G	P62309	--	-0,201		1,79E-02	
DBNL	drebrin like	Q62418	--	-0,2		8,10E-02	
TROVE2	TROVE domain family member 2	O08848	--	-0,198		3,23E-02	
MYBBP1A	MYB binding protein 1a	Q7TPV4	Q7TPV4	-0,196	-0,13	2,05E-06	8,89E-04
**RRM1**	**ribonucleotide reductase catalytic subunit M1**	P07742	P07742	-0,196	-0,045	5,46E-04	6,32E-03
TTLL12	tubulin tyrosine ligase like 12	Q3UDE2	--	-0,195		2,79E-02	
KHSRP	KH-type splicing regulatory protein	Q3U0V1	--	-0,195		2,51E-04	
**RPL17**	**ribosomal protein L17**	Q9CPR4	--	-0,195		1,33E-01	
RNPS1	RNA binding protein with serine rich domain 1	Q99M28	--	-0,195		5,18E-02	
SEPT11	septin 11	Q8C1B7	--	-0,195		1,21E-01	
**GFPT1**	**glutamine--fructose-6-phosphate transaminase 1**	P47856	--	-0,195		3,76E-01	
COPS6	COP9 signalosome subunit 6	O88545	--	-0,195		4,72E-03	
ARPP19	cAMP regulated phosphoprotein 19	P56212	--	-0,194		3,87E-01	
DBI	diazepam binding inhibitor, acyl-CoA binding protein	P31786	--	-0,193		3,92E-01	
YBX1	Y-box binding protein 1	P62960	P62960	-0,193	-0,123	1,50E-01	4,58E-01
EHD2	EH domain containing 2	Q8BH64	Q8BH64	-0,192	-0,192	9,64E-04	3,30E-03
**PRDX6**	**peroxiredoxin 6**	O08709	--	-0,191		2,74E-04	
**RAN**	**RAN, member RAS oncogene family**	P62827	--	-0,191		5,84E-02	
DPY30	dpy-30, histone methyltransferase complex regulatory subunit	Q99LT0	--	-0,189		6,04E-02	
SDF4	stromal cell derived factor 4	Q61112	--	-0,189		3,54E-01	
**EIF3A**	**eukaryotic translation initiation factor 3 subunit A**	P23116	P23116	-0,189	-0,001	1,21E-08	4,95E-05
DDX5	DEAD-box helicase 5	Q61656	--	-0,188		3,41E-04	
HNRNPLL	heterogeneous nuclear ribonucleoprotein L like	Q921F4	--	-0,188		1,22E-03	
**FADS1**	**fatty acid desaturase 1**	Q920L1	--	-0,187		7,75E-03	
**RPL6**	**ribosomal protein L6**	P47911	P47911	-0,187	-0,169	2,87E-04	4,22E-03
SRM	spermidine synthase	Q64674	--	-0,186		1,05E-02	
**RPL7A**	**ribosomal protein L7a**	P12970	--	-0,186		2,80E-02	
BAZ1B	bromodomain adjacent to zinc finger domain 1B	Q9Z277	--	-0,185		2,39E-01	
**EIF4A1**	**eukaryotic translation initiation factor 4A1**	P60843	--	-0,185		4,42E-01	
**RPL23**	**ribosomal protein L23**	P62830	--	-0,184		5,54E-02	
UBA2	ubiquitin like modifier activating enzyme 2	Q9Z1F9	--	-0,183		2,05E-02	
ANXA4	annexin A4	P97429	--	-0,181		1,95E-01	
ATXN2L	ataxin 2 like	Q7TQH0	--	-0,18		1,07E-01	
NME1	NME/NM23 nucleoside diphosphate kinase 1	P15532	--	-0,179		2,79E-02	
**PHGDH**	**phosphoglycerate dehydrogenase**	Q61753	--	-0,179		8,94E-02	

NCOA5	nuclear receptor coactivator 5	Q91W39	--	-0,178		4,14E-02	
TUBA1A	tubulin alpha 1a	P68369	--	-0,178		2,47E-01	
NAA50	N(alpha)-acetyltransferase 50, NatE catalytic subunit	Q6PGB6	--	-0,177		3,43E-02	
IDE	insulin degrading enzyme	Q9JHR7	Q9JHR7	-0,176	0,181	2,11E-02	6,05E-01
FKBP14	FK506 binding protein 14	P59024	--	-0,175		1,46E-01	
RBM14	RNA binding motif protein 14	Q8C2Q3	--	-0,174		2,11E-01	
**CYP51A1**	**cytochrome P450 family 51 subfamily A member 1**	Q8K0C4	Q8K0C4	-0,174	-0,114	2,43E-05	1,13E-05
SHC1	SHC adaptor protein 1	P98083	--	-0,174		4,80E-01	
SUGT1	SGT1 homolog, MIS12 kinetochore complex assembly cochaperone	Q9CX34	--	-0,173		8,54E-06	
SELENOS	selenoprotein S	Q9BCZ4	--	-0,172		2,25E-01	
SNRNP70	small nuclear ribonucleoprotein U1 subunit 70	Q62376	Q62376	-0,172	0,12	2,03E-04	1,35E-01
SET	SET nuclear proto-oncogene	Q9EQU5	--	-0,172		1,95E-03	
YARS	tyrosyl-tRNA synthetase	Q91WQ3	Q91WQ3	-0,172	-0,21	2,57E-02	1,04E-01
AARS	alanyl-tRNA synthetase	Q8BGQ7	Q8BGQ7	-0,172	-0,088	2,46E-02	1,73E-02
SUMO1	small ubiquitin-like modifier 1	P63166	--	-0,171		2,08E-03	
**HSP90AA1**	**heat shock protein 90 alpha family class A member 1**	P07901	P07901	-0,171	-0,075	5,55E-03	1,54E-02
**RPS16**	**ribosomal protein S16**	P14131	P14131	-0,17	-0,093	7,13E-06	1,86E-03
IMPDH2	inosine monophosphate dehydrogenase 2	P24547	P24547	-0,169	-0,074	2,93E-03	2,07E-02
**RPL18**	**ribosomal protein L18**	P35980	--	-0,169		4,50E-04	
HDGF	heparin binding growth factor	P51859	P51859	-0,168	-0,034	9,42E-03	3,43E-01
ILK	integrin linked kinase	O55222	--	-0,168		1,46E-02	
ABCB7	ATP binding cassette subfamily B member 7	Q61102	--	-0,166		2,18E-01	
PAK2	p21 (RAC1) activated kinase 2	Q8CIN4	--	-0,166		3,74E-02	
HDGFL2	HDGF like 2	Q3UMU9	--	-0,163		2,28E-02	
ASAH1	N-acylsphingosine amidohydrolase 1	Q9WV54	Q9WV54	-0,163	-0,151	1,70E-01	4,47E-04
CTBP1	C-terminal binding protein 1	O88712	O88712	-0,162	-0,1	1,06E-02	9,31E-02
LMNB1	lamin B1	P14733	P14733	-0,162	-0,048	4,10E-01	2,32E-01
SSPN	sarcospan	Q62147	--	-0,161		1,77E-01	
STIP1	stress induced phosphoprotein 1	Q60864	--	-0,161		2,69E-02	
**RPS6**	**ribosomal protein S6**	P62754	--	-0,161		1,41E-01	
MAGED1	MAGE family member D1	Q9QYH6	--	-0,16		5,87E-01	
Srrm2	serine/arginine repetitive matrix 2	Q8BTI8	--	-0,16		3,20E-02	
GTPBP4	GTP binding protein 4	Q99ME9	Q99ME9	-0,16	-0,29	8,84E-03	3,22E-02
BTF3L4	basic transcription factor 3 like 4	Q9CQH7	--	-0,16		3,63E-02	
YAP1	Yes associated protein 1	P46938	--	-0,159		1,96E-01	
**ENO1**	**enolase 1**	P17182	--	-0,159		4,48E-02	
**RPS4Y1**	**ribosomal protein S4, Y-linked 1**	P62702	--	-0,159		3,73E-01	
NUDC	nuclear distribution C, dynein complex regulator	O35685	--	-0,158		1,56E-02	
DHX57	DExH-box helicase 57	Q6P5D3	--	-0,158		2,09E-02	

**TOP1**	**topoisomerase (DNA) I**	Q04750	Q04750	-0,157	-0,032	5,94E-01	1,35E-01
PLAU	plasminogen activator, urokinase	P06869	--	-0,156		4,82E-01	
**TKT**	**transketolase**	P40142	--	-0,156		1,96E-02	
**Rpl34 (incl**	**ribosomal protein L34**	Q9D1R9	--	-0,156		1,54E-03	
HNRNPDL	heterogeneous nuclear ribonucleoprotein D like	Q9Z130	--	-0,156		3,30E-02	
COPA	coatomer protein complex subunit alpha	Q8CIE6	Q8CIE6	-0,156	0,009	1,36E-01	3,07E-03
**PFKP**	**phosphofructokinase, platelet**	Q9WUA3	Q9WUA3	-0,156	0,157	3,66E-01	4,83E-01
**BAX**	**BCL2 associated X, apoptosis regulator**	Q07813	--	-0,155		1,01E-01	
**EIF4B**	**eukaryotic translation initiation factor 4B**	Q8BGD9	--	-0,155		2,06E-02	
GLO1	glyoxalase I	Q9CPU0	--	-0,153		1,74E-01	
KLC1	kinesin light chain 1	O88447	O88447	-0,153	0,046	2,35E-01	4,07E-01
MAT2A	methionine adenosyltransferase 2A	Q3THS6	Q3THS6	-0,152	0,014	5,94E-02	3,94E-01
H2AFY	H2A histone family member Y	Q9QZQ8	--	-0,152		3,15E-01	
SRSF7	serine and arginine rich splicing factor 7	Q8BL97	--	-0,151		4,85E-04	
TGFB1	transforming growth factor beta 1	P04202	P04202	-0,149	0,32	2,05E-01	4,70E-01
USP15	ubiquitin specific peptidase 15	Q8R5H1	--	-0,149		2,93E-01	
**TXNIP**	**thioredoxin interacting protein**	Q8BG60	Q8BG60	-0,148	0,053	9,77E-02	5,79E-01
OGT	O-linked N-acetylglucosamine (GlcNAc) transferase	Q8CGY8	--	-0,148		3,68E-01	
DDX21	DExD-box helicase 21	Q9JIK5	Q9JIK5	-0,147	-0,098	2,32E-02	1,96E-03
COL1A1	collagen type I alpha 1 chain	P11087	P11087	-0,147	-0,156	1,11E-04	2,40E-12
**RPS9**	**ribosomal protein S9**	Q6ZWN5	--	-0,147		2,38E-02	
UMPS	uridine monophosphate synthetase	P13439	--	-0,146		3,77E-02	
THUMPD1	THUMP domain containing 1	Q99J36	--	-0,146		1,18E-02	
**EIF4E**	**eukaryotic translation initiation factor 4E**	P63073	--	-0,146		1,92E-01	
**ACSL4**	**acyl-CoA synthetase long-chain family member 4**	Q9QUJ7	--	-0,145		5,11E-01	
CR1L	complement C3b/C4b receptor 1 like	Q64735	Q64735	-0,145	0,072	5,44E-02	2,48E-01
SLC7A5	solute carrier family 7 member 5	Q9Z127	Q9Z127	-0,145	-0,127	2,99E-02	2,64E-03
**PFKL**	**phosphofructokinase, liver type**	P12382	P12382	-0,145	0,038	9,52E-07	1,43E-02
**PARK7**	**Parkinsonism associated deglycase**	Q99LX0	--	-0,144		2,99E-03	
TBL3	transducin beta like 3	Q8C4J7	--	-0,144		3,63E-02	
ARHGEF2	Rho/Rac guanine nucleotide exchange factor 2	Q60875	--	-0,143		9,71E-03	
DDI2	DNA damage inducible 1 homolog 2	A2ADY9	--	-0,143		4,71E-01	
NARS	asparaginyl-tRNA synthetase	Q8BP47	Q8BP47	-0,143	0,007	2,57E-02	1,58E-02
NFYA	nuclear transcription factor Y subunit alpha	P23708	P23708	-0,143	0,157	5,04E-01	4,51E-01
**MT-CO3**	**cytochrome c oxidase III**	P00416	--	-0,142		3,06E-02	
DDX17	DEAD-box helicase 17	Q501J6	--	-0,141		3,03E-07	
**GPX8**	**glutathione peroxidase 8 (putative)**	Q9D7B7	--	-0,14		3,89E-01	

**EIF3C**	**eukaryotic translation initiation factor 3 subunit C**	Q8R1B4	Q8R1B4	-0,139	-0,04	3,90E-04	4,17E-02
G3BP1	G3BP stress granule assembly factor 1	P97855	--	-0,138		7,94E-09	
AQP1	aquaporin 1 (Colton blood group)	Q02013	--	-0,138		3,07E-01	
TJP1	tight junction protein 1	P39447	--	-0,137		4,14E-02	
RBM39	RNA binding motif protein 39	Q8VH51	--	-0,136		4,14E-02	
EXOSC6	exosome component 6	Q8BTW3	--	-0,136		1,17E-01	
IREB2	iron responsive element binding protein 2	Q811J3	--	-0,136		4,28E-01	
SRSF1	serine and arginine rich splicing factor 1	Q6PDM2	Q6PDM2	-0,135	0,285	2,52E-01	4,70E-01
CBX5	chromobox 5	Q61686	--	-0,135		3,86E-02	
**EIF5**	**eukaryotic translation initiation factor 5**	P59325	--	-0,134		7,73E-03	
SSRP1	structure specific recognition protein 1	Q08943	--	-0,134		2,72E-02	
CAB39	calcium binding protein 39	Q06138	--	-0,133		3,09E-02	
TAGLN2	transgelin 2	Q9WVA4	--	-0,133		7,53E-02	
FSCN1	fascin actin-bundling protein 1	Q61553	Q61553	-0,133	-0,08	7,89E-02	2,20E-02
GRSF1	G-rich RNA sequence binding factor 1	Q8C5Q4	--	-0,132		5,47E-02	
HDLBP	high density lipoprotein binding protein	Q8VDJ3	Q8VDJ3	-0,13	-0,059	4,46E-02	1,41E-02
CARS	cysteinyl-tRNA synthetase	Q9ER72	Q9ER72	-0,127	0,017	1,49E-02	1,72E-01
CHD4	chromodomain helicase DNA binding protein 4	Q6PDQ2	Q6PDQ2	-0,126	-0,125	1,18E-05	2,14E-02
CUL2	cullin 2	Q9D4H8	--	-0,126		4,16E-02	
GNL3	G protein nucleolar 3	Q8CI11	--	-0,126		3,80E-02	
FRS2	fibroblast growth factor receptor substrate 2	Q8C180	--	-0,126		3,43E-01	
QDPR	quinoid dihydropteridine reductase	Q8BVI4	--	-0,123		3,82E-01	
PTGIS	prostaglandin I2 synthase	O35074	--	-0,122		3,85E-03	
COL5A2	collagen type V alpha 2 chain	Q3U962	Q3U962	-0,122	-0,204	1,32E-02	4,19E-05
NAB2	NGFI-A binding protein 2	Q61127	Q61127	-0,12	0,071	2,16E-01	5,94E-01
LYAR	Ly1 antibody reactive	Q08288	Q08288	-0,12	-0,135	3,41E-03	1,80E-02
ATP1A1	ATPase Na+/K+ transporting subunit alpha 1	Q8VDN2	--	-0,12		1,45E-05	
FAM129A	family with sequence similarity 129 member A	Q3UW53	--	-0,12		3,05E-01	
SND1	staphylococcal nuclease and tudor domain containing 1	Q78PY7	--	-0,12		1,99E-04	
RBM8A	RNA binding motif protein 8A	Q9CWZ3	--	-0,119		1,37E-01	
CERS5	ceramide synthase 5	Q9D6K9	Q9D6K9	-0,119	0,176	5,71E-01	6,94E-02
LARS	leucyl-tRNA synthetase	Q8BMJ2	Q8BMJ2	-0,118	-0,086	8,85E-02	4,60E-02
HNRNPK	heterogeneous nuclear ribonucleoprotein K	P61979	P61979	-0,117	-0,085	4,07E-01	6,08E-03
CTH	cystathionine gamma-lyase	Q8VCN5	--	-0,117		5,97E-01	

SEC23A	Sec23 homolog A, coat complex II component	Q01405	--	-0,116		5,37E-02	
**PRDX4**	**peroxiredoxin 4**	O08807	--	-0,116		1,86E-01	
DYNLL2	dynein light chain LC8-type 2	Q9D0M5	--	-0,116		8,05E-02	
FBL	fibrillarin	P35550	--	-0,115		1,58E-03	
EEF1A1	eukaryotic translation elongation factor 1 alpha 1	P10126	--	-0,115		5,82E-01	
PDCD11	programmed cell death 11	Q6NS46	--	-0,114		1,35E-03	
**PGLS**	**6-phosphogluconolactonase**	Q9CQ60	--	-0,114		3,28E-01	
COPS4	COP9 signalosome subunit 4	O88544	--	-0,114		5,74E-01	
VARS	valyl-tRNA synthetase	Q9Z1Q9	Q9Z1Q9	-0,114	-0,207	9,44E-08	8,51E-03
LSM1	LSM1 homolog, mRNA degradation associated	Q8VC85	--	-0,113		6,00E-01	
SSB	Sjogren syndrome antigen B	P32067	P32067	-0,113	-0,193	1,41E-02	1,66E-02
**IGFBP7**	**insulin like growth factor binding protein 7**	Q61581	Q61581	-0,113	-0,172	5,22E-01	1,44E-02
MAPK14	mitogen-activated protein kinase 14	P47811	P47811	-0,112	0,199	5,99E-01	2,36E-01
CDC5L	cell division cycle 5 like	Q6A068	--	-0,111		2,01E-02	
SMARCE1	SWI/SNF related, matrix associated, actin dependent regulator of chromatin, subfamily e, member 1	O54941	--	-0,111		6,01E-01	
WDR1	WD repeat domain 1	O88342	--	-0,11		4,79E-01	
DDX6	DEAD-box helicase 6	P54823	--	-0,11		3,79E-02	
ANXA7	annexin A7	Q07076	--	-0,109		4,29E-01	
SMAD4	SMAD family member 4	P97471	P97471	-0,109	-0,223	3,87E-01	4,02E-01
CLIC1	chloride intracellular channel 1	Q9Z1Q5	--	-0,109		2,54E-01	
SBDS	SBDS, ribosome maturation factor	P70122	--	-0,108		3,00E-01	
PES1	pescadillo ribosomal biogenesis factor 1	Q9EQ61	Q9EQ61	-0,107	0,07	8,05E-02	2,57E-01
PDXDC1	pyridoxal dependent decarboxylase domain containing 1	Q99K01	--	-0,106		3,00E-01	
USP14	ubiquitin specific peptidase 14	Q9JMA1	--	-0,105		9,99E-03	
RNH1	ribonuclease/angiogenin inhibitor 1	Q91VI7	--	-0,105		3,66E-01	
YWHAE	tyrosine 3-monooxygenase/tryptophan 5-monooxygenase activation protein epsilon	P62259	--	-0,103		1,31E-01	
HNRNPL	heterogeneous nuclear ribonucleoprotein L	Q8R081	--	-0,102		9,52E-02	
SERBP1	SERPINE1 mRNA binding protein 1	Q9CY58	Q9CY58	-0,1	-0,023	3,09E-03	2,01E-02
CCAR1	cell division cycle and apoptosis regulator 1	Q8CH18	--	-0,099		1,39E-02	
YWHAH	tyrosine 3-monooxygenase/tryptophan 5-monooxygenase activation protein eta	P68510	--	-0,099		3,53E-02	

NAP1L1	nucleosome assembly protein 1 like 1	P28656	--	-0,099		3,44E-04	
CHMP3	charged multivesicular body protein 3	Q9CQ10	--	-0,098		2,35E-01	
UBE4B	ubiquitination factor E4B	Q9ES00	--	-0,097		5,79E-03	
CARM1	coactivator associated arginine methyltransferase 1	Q9WVG6	--	-0,096		1,97E-01	
**PKM**	**pyruvate kinase, muscle**	P52480	--	-0,094		1,82E-04	
TRAM1	translocation associated membrane protein 1	Q91V04	--	-0,093		1,08E-02	
LGALS1	galectin 1	P16045	--	-0,093		1,66E-01	
PKMYT1	protein kinase, membrane associated tyrosine/threonine 1	Q9ESG9	Q9ESG9	-0,09	0,337	3,31E-01	2,80E-01
IRF2BP2	interferon regulatory factor 2 binding protein 2	E9Q1P8	--	-0,09		5,66E-01	
NMT1	N-myristoyltransferase 1	O70310	--	-0,09		1,80E-02	
TCERG1	transcription elongation regulator 1	Q8CGF7	--	-0,09		2,43E-08	
YWHAB	tyrosine 3-monooxygenase/tryptophan 5-monooxygenase activation protein beta	Q9CQV8	--	-0,089		2,82E-02	
DHX9	DExH-box helicase 9	O70133	O70133	-0,089	-0,007	5,17E-02	2,51E-02
TFRC	transferrin receptor	Q62351	--	-0,089		3,09E-02	
**PRDX1**	**peroxiredoxin 1**	P35700	--	-0,088		5,06E-01	
**EIF3K**	**eukaryotic translation initiation factor 3 subunit K**	Q9DBZ5	--	-0,087		3,57E-02	
NT5C2	5'-nucleotidase, cytosolic II	Q3V1L4	--	-0,085		1,08E-02	
**POLDIP3**	**DNA polymerase delta interacting protein 3**	Q8BG81	Q8BG81	-0,083	-0,013	3,70E-03	3,26E-02
**HSP90AB**	**heat shock protein 90 alpha family class B member 1**	P11499	--	-0,083		4,90E-02	
VPS25	vacuolar protein sorting 25 homolog	Q9CQ80	--	-0,083		1,02E-01	
TEAD1	TEA domain transcription factor 1	P30051	--	-0,082		3,32E-01	
PGM1	phosphoglucomutase 1	Q9D0F9	--	-0,082		2,92E-02	
DYNLRB1	dynein light chain roadblock-type 1	P62627	--	-0,082		4,31E-01	
HP1BP3	heterochromatin protein 1 binding protein 3	Q3TEA8	--	-0,081		1,92E-04	
TOR1A	torsin family 1 member A	Q9ER39	--	-0,081		5,30E-01	
MAP1B	microtubule associated protein 1B	P14873	--	-0,08		6,59E-03	
PSMD7	proteasome 26S subunit, non-ATPase 7	P26516	--	-0,078		1,96E-03	
NUP155	nucleoporin 155	Q99P88	--	-0,078		1,61E-03	
**PGK1**	**phosphoglycerate kinase 1**	P09411	P09411	-0,077	0,087	2,54E-01	3,93E-02
CAD	carbamoyl-phosphate synthetase 2, aspartate transcarbamylase, and dihydroorotase	B2RQC6	B2RQC6	-0,076	0,075	9,77E-04	1,13E-02
Ewsr1	Ewing sarcoma breakpoint region 1	Q61545	--	-0,076		5,81E-02	

COPB2	coatomer protein complex subunit beta 2	O55029	--	-0,076		1,52E-02	
GMPS	guanine monophosphate synthase	Q3THK7	--	-0,075		4,72E-02	
SEC23IP	SEC23 interacting protein	Q6NZC7	--	-0,074		2,54E-01	
ANGPTL3	angiopoietin like 3	Q9R182	Q9R182	-0,073	0,044	1,34E-01	4,82E-01
FAS	Fas cell surface death receptor	P25446	P25446	-0,073	0,477	1,43E-01	2,36E-01
**RPS27A**	**ribosomal protein S27a**	P62983	--	-0,071		3,48E-01	
MAPK1	mitogen-activated protein kinase 1	P63085	--	-0,07		1,50E-01	
PNP	purine nucleoside phosphorylase	P23492	--	-0,07		9,34E-02	
**PDK3**	**pyruvate dehydrogenase kinase 3**	Q922H2	--	-0,07		2,02E-02	
**AKT1**	**AKT serine/threonine kinase 1**	P31750	P31750	-0,07	0,115	3,64E-01	4,31E-01
PREP	prolyl endopeptidase	Q9QUR6	--	-0,069		2,00E-01	
**PCK2**	**phosphoenolpyruvate carboxykinase 2, mitochondrial**	Q8BH04	--	-0,069		2,03E-02	
TSN	translin	Q62348	--	-0,069		4,22E-02	
PEPD	peptidase D	Q11136	--	-0,068		4,39E-02	
DYNLT1	dynein light chain Tctex-type 1	P51807	--	-0,068		3,09E-01	
EPHB1	EPH receptor B1	Q8CBF3	--	-0,067		1,18E-01	
SLC3A2	solute carrier family 3 member 2	P10852	P10852	-0,067	-0,07	4,35E-03	3,07E-02
NSUN2	NOP2/Sun RNA methyltransferase family member 2	Q1HFZ0	Q1HFZ0	-0,066	0,016	4,55E-03	4,55E-02
**ACTR3**	**ARP3 actin related protein 3 homolog**	Q99JY9	--	-0,066		4,57E-02	
VPS18	VPS18, CORVET/HOPS core subunit	Q8R307	--	-0,065		4,05E-01	
SLIT2	slit guidance ligand 2	Q9R1B9	--	-0,065		2,86E-01	
SLC25A13	solute carrier family 25 member 13	Q9QXX4	Q9QXX4	-0,064	0,216	3,06E-01	2,63E-01
KARS	lysyl-tRNA synthetase	Q99MN1	Q99MN1	-0,064	-0,073	1,14E-01	3,29E-02
STUB1	STIP1 homology and U-box containing protein 1	Q9WUD1	--	-0,064		4,36E-01	
**EIF3E**	**eukaryotic translation initiation factor 3 subunit E**	P60229	--	-0,063		2,74E-03	
SH3PXD2B	SH3 and PX domains 2B	A2AAY5	--	-0,063		2,88E-03	
ADSL	adenylosuccinate lyase	P54822	--	-0,061		3,57E-02	
PIN1	peptidylprolyl cis/trans isomerase, NIMA-interacting 1	Q9QUR7	Q9QUR7	-0,059	0,046	1,05E-01	5,72E-01
**TOP2B**	**topoisomerase (DNA) II beta**	Q64511	Q64511	-0,058	-0,067	4,41E-01	3,05E-01
CDKN1A	cyclin dependent kinase inhibitor 1A	P39689	--	-0,058		1,73E-01	
RPL22	ribosomal protein L22	P67984	--	-0,058		5,99E-02	
CSPG4	chondroitin sulfate proteoglycan 4	Q8VHY0	Q8VHY0	-0,058	0,025	2,48E-07	4,71E-03
BCLAF1	BCL2 associated transcription factor 1	Q8K019	Q8K019	-0,056	0,03	3,05E-02	4,53E-02
COL2A1	collagen type II alpha 1 chain	P28481	P28481	-0,056	-0,015	5,72E-01	5,86E-01
CTPS1	CTP synthase 1	P70698	--	-0,056		1,74E-01	
CDC42BPB	CDC42 binding protein kinase beta	Q7TT50	--	-0,055		6,95E-02	
FERMT2	fermitin family member 2	Q8CIB5	Q8CIB5	-0,055	0,036	2,64E-01	5,58E-01
**RPLP2**	**ribosomal protein lateral stalk subunit P2**	P99027	--	-0,054		2,18E-01	

QKI	QKI, KH domain containing RNA binding	Q9QYS9	--	-0,053		8,89E-02	
**EIF3L**	**eukaryotic translation initiation factor 3 subunit L**	Q8QZY1	--	-0,053		5,81E-03	
CDKN2A	cyclin dependent kinase inhibitor 2A	P51480	--	-0,051		1,87E-01	
NPM1	nucleophosmin	Q61937	--	-0,051		1,23E-01	
PRKCI	protein kinase C iota	Q62074	Q62074	-0,05	-0,003	5,91E-01	6,05E-01
**EIF3F**	**eukaryotic translation initiation factor 3 subunit F**	Q9DCH4	--	-0,05		1,17E-02	
KPNB1	karyopherin subunit beta 1	P70168	--	-0,05		2,81E-02	
TNPO2	transportin 2	Q99LG2	--	-0,05		2,99E-03	
**EIF2B2**	**eukaryotic translation initiation factor 2B subunit beta**	Q99LD9	--	-0,05		4,40E-01	
LMF2	lipase maturation factor 2	Q8C3X8	--	-0,049		1,00E-01	
RNMT	RNA guanine-7 methyltransferase	Q9D0L8	--	-0,048		5,61E-01	
SFPQ	splicing factor proline and glutamine rich	Q8VIJ6	Q8VIJ6	-0,048	0,088	3,92E-05	2,82E-02
**G6PD**	**glucose-6-phosphate dehydrogenase**	Q00612	--	-0,048		4,82E-02	
ADAM10	ADAM metallopeptidase domain 10	O35598	--	-0,046		5,23E-01	
F2	coagulation factor II, thrombin	P19221	P19221	-0,046	-0,233	4,38E-01	5,26E-01
SYNCRIP	synaptotagmin binding cytoplasmic RNA interacting protein	Q7TMK9	--	-0,046		1,26E-01	
ITGA11	integrin subunit alpha 11	P61622	--	-0,045		1,26E-01	
MAT2B	methionine adenosyltransferase 2B	Q99LB6	Q99LB6	-0,045	0,06	5,93E-01	3,36E-01
NUP93	nucleoporin 93	Q8BJ71	--	-0,044		7,01E-03	
VPS45	vacuolar protein sorting 45 homolog	P97390	--	-0,043		4,89E-01	
TJP2	tight junction protein 2	Q9Z0U1	--	-0,043		6,05E-01	
**EIF3I**	**eukaryotic translation initiation factor 3 subunit I**	Q9QZD9	--	-0,042		7,41E-03	
MTA2	metastasis associated 1 family member 2	Q9R190	--	-0,042		7,79E-02	
LIMS1	LIM zinc finger domain containing 1	Q99JW4	--	-0,041		5,00E-01	
**TXNL1**	**thioredoxin like 1**	Q8CDN6	--	-0,039		7,18E-02	
COL1A2	collagen type I alpha 2 chain	Q01149	Q01149	-0,039	-0,107	7,81E-05	1,50E-11
NPEPPS	aminopeptidase puromycin sensitive	Q11011	--	-0,038		3,63E-02	
CTNNBL1	catenin beta like 1	Q9CWL8	--	-0,037		3,09E-02	
LARP1	La ribonucleoprotein domain family member 1	Q6ZQ58	Q6ZQ58	-0,037	-0,036	5,92E-01	5,12E-01
UBA1	ubiquitin like modifier activating enzyme 1	Q02053	--	-0,037		1,49E-02	
ABCF2	ATP binding cassette subfamily F member 2	Q99LE6	--	-0,036		3,52E-02	
LIPA	lipase A, lysosomal acid type	Q9Z0M5	--	-0,036		1,04E-02	
SF3A1	splicing factor 3a subunit 1	Q8K4Z5	--	-0,034		3,84E-02	

**RPLP0**	**ribosomal protein lateral stalk subunit P0**	P14869	--	-0,033		4,94E-01	
ANXA5	annexin A5	P48036	P48036	-0,033	0,093	6,01E-01	5,44E-01
**RPL9**	**ribosomal protein L9**	P51410	--	-0,032		7,21E-03	
**HSPH1**	**heat shock protein family H (Hsp110) member 1**	Q61699	--	-0,031		1,19E-02	
**ACOT7**	**acyl-CoA thioesterase 7**	Q91V12	--	-0,03		9,47E-02	
PSMC6	proteasome 26S subunit, ATPase 6	P62334	P62334	-0,028	-0,136	6,14E-02	3,17E-02
LOX	lysyl oxidase	P28301	--	-0,027		7,37E-02	
**GAPDH**	**glyceraldehyde-3-phosphate dehydrogenase**	P16858	P16858	-0,027	-0,024	2,09E-02	4,78E-02
ESD	esterase D	Q9R0P3	--	-0,027		5,94E-01	
**GSK3B**	**glycogen synthase kinase 3 beta**	Q9WV60	--	-0,026		4,85E-01	
MSN	moesin	P26041	--	-0,026		7,69E-02	
CUL4B	cullin 4B	A2A432	--	-0,026		6,05E-01	
NID1	nidogen 1	P10493	--	-0,025		1,60E-01	
RBM17	RNA binding motif protein 17	Q8JZX4	Q8JZX4	-0,024	0,179	5,40E-01	3,06E-01
**STAT3**	**signal transducer and activator of transcription 3**	P42227	P42227	-0,024	0,008	1,48E-01	3,81E-01
IPO7	importin 7	Q9EPL8	--	-0,023		3,26E-02	
SNRNP200	small nuclear ribonucleoprotein U5 subunit 200	Q6P4T2	--	-0,023		3,04E-04	
IFRD1	interferon related developmental regulator 1	P19182	P19182	-0,021	-0,165	3,85E-01	6,05E-01
FHL1	four and a half LIM domains 1	P97447	P97447	-0,02	0,068	3,37E-01	6,92E-03
**HSPG2**	**heparan sulfate proteoglycan 2**	Q05793	Q05793	-0,019	-0,059	4,87E-01	4,59E-05
ARF1	ADP ribosylation factor 1	P84078	--	-0,019		5,22E-01	
IFITM3	interferon induced transmembrane protein 3	Q9CQW9	Q9CQW9	-0,019	-0,078	3,74E-02	6,17E-03
**SDHA**	**succinate dehydrogenase complex flavoprotein subunit A**	Q8K2B3	Q8K2B3	-0,017	-0,025	3,41E-01	1,88E-01
ARHGAP1	Rho GTPase activating protein 1	Q5FWK3	--	-0,016		4,67E-01	
AP3S1	adaptor related protein complex 3 sigma 1 subunit	Q9DCR2	--	-0,016		1,07E-01	
ANXA1	annexin A1	P10107	--	-0,015		5,06E-01	
MPRIP	myosin phosphatase Rho interacting protein	P97434	--	-0,015		2,99E-02	
DDX1	DEAD-box helicase 1	Q91VR5	Q91VR5	-0,015	-0,196	1,23E-01	5,43E-03
HCFC1	host cell factor C1	Q61191	Q61191	-0,015	0,162	2,81E-02	3,16E-01
COL6A2	collagen type VI alpha 2 chain	Q02788	--	-0,015		2,24E-03	
ARL1	ADP ribosylation factor like GTPase 1	P61211	--	-0,015		1,52E-01	
SERPINH1	serpin family H member 1	P19324	P19324	-0,013	-0,146	5,74E-01	2,51E-04
NQO2	N-ribosyldihydronicotinamide:quinone reductase 2	Q9JI75	--	-0,012		5,96E-01	
EHD1	EH domain containing 1	Q9WVK4	--	-0,012		4,77E-01	

PSMD2	proteasome 26S subunit, non-ATPase 2	Q8VDM4	--	-0,012		8,89E-02	
PAFAH1B1	platelet activating factor acetylhydrolase 1b regulatory subunit 1	P63005	--	-0,012		6,08E-01	
**CBR1**	**carbonyl reductase 1**	P48758	--	-0,011		1,73E-01	
**ARPC4**	**actin related protein 2/3 complex subunit 4**	P59999	--	-0,011		4,88E-02	
GYG1	glycogenin 1	Q9R062	Q9R062	-0,01	0,157	1,91E-01	3,99E-01
PREB	prolactin regulatory element binding	Q9WUQ2	--	-0,008		9,50E-02	
BAG6	BCL2 associated athanogene 6	Q9Z1R2	--	-0,008		1,74E-03	
ARHGDIB	Rho GDP dissociation inhibitor beta	Q61599	--	-0,007		3,79E-01	
EDIL3	EGF like repeats and discoidin domains 3	O35474	--	-0,007		1,48E-01	
PRKCB	protein kinase C beta	P68404	P68404	-0,006	-0,002	5,73E-01	5,73E-01
**SOD1**	**superoxide dismutase 1**	P08228	--	-0,006		4,63E-01	
HMOX2	heme oxygenase 2	O70252	--	-0,006		1,66E-01	
GANAB	glucosidase II alpha subunit	Q8BHN3	Q8BHN3	-0,006	0,004	5,16E-03	1,28E-03
RUVBL1	RuvB like AAA ATPase 1	P60122	--	-0,006		1,33E-01	
HARS	histidyl-tRNA synthetase	Q61035	--	-0,005		3,23E-01	
VPS35	VPS35, retromer complex component	Q9EQH3	--	-0,005		2,59E-01	
FLNC	filamin C	Q8VHX6	Q8VHX6	-0,004	0,035	3,51E-05	4,65E-02
**EIF3B**	**eukaryotic translation initiation factor 3 subunit B**	Q8JZQ9	Q8JZQ9	-0,004	-0,026	6,60E-05	3,98E-02
PPP2CA	protein phosphatase 2 catalytic subunit alpha	P63330	--	-0,003		4,64E-01	
HNRNPAB	heterogeneous nuclear ribonucleoprotein A/B	Q99020	--	-0,002		1,18E-01	
SF3B1	splicing factor 3b subunit 1	Q99NB9	--	-0,002		1,28E-03	
PPP3CA	protein phosphatase 3 catalytic subunit alpha	P63328	--	-0,001		1,36E-01	
PPIF	peptidylprolyl isomerase F	Q99KR7	--	-0,001		6,07E-01	
MTPN	myotrophin	P62774	P62774	0	-0,163	1,31E-01	2,65E-01
CNN3	calponin 3	Q9DAW9	--	0		1,96E-02	
**RPS17**	**ribosomal protein S17**	P63276	--	0,001		3,36E-01	
**IGF2R**	**insulin like growth factor 2 receptor**	Q07113	--	0,002		2,93E-02	
LZIC	leucine zipper and CTNNBIP1 domain containing	Q8K3C3	--	0,003		1,53E-01	
PSMD11	proteasome 26S subunit, non-ATPase 11	Q8BG32	--	0,005		1,92E-02	
**MYO1B**	**myosin IB**	P46735	--	0,005		1,06E-02	
YWHAG	tyrosine 3-monooxygenase/tryptophan 5-monooxygenase activation protein gamma	P61982	--	0,006		1,13E-01	

**GPX1**	**glutathione peroxidase 1**	P11352	--	0,006		5,09E-01	
COPB1	coatomer protein complex subunit beta 1	Q9JIF7	Q9JIF7	0,006	0,08	1,14E-02	4,49E-02
SLC25A24	solute carrier family 25 member 24	Q8BMD8	--	0,007		1,79E-02	
ATXN10	ataxin 10	P28658	--	0,007		1,87E-02	
**EIF2S1**	**eukaryotic translation initiation factor 2 subunit alpha**	Q6ZWX6	--	0,007		2,26E-01	
COL5A1	collagen type V alpha 1 chain	O88207	O88207	0,007	-0,02	3,14E-01	4,41E-05
NCAM1	neural cell adhesion molecule 1	P13595	--	0,009		3,30E-01	
PTPA	protein phosphatase 2 phosphatase activator	P58389	P58389	0,009	0,056	2,06E-01	3,18E-01
CAV1	caveolin 1	P49817	--	0,01		5,95E-01	
**HSPD1**	**heat shock protein family D (Hsp60) member 1**	P63038	--	0,01		3,07E-03	
**ALDH1L2**	**aldehyde dehydrogenase 1 family member L2**	Q8K009	Q8K009	0,012	-0,008	2,56E-01	3,08E-03
Rplp1 (incl	ribosomal protein, large, P1	P47955	--	0,012		1,82E-01	
RRAGA	Ras related GTP binding A	Q80X95	--	0,014		7,87E-02	
CSK	CSK, non-receptor tyrosine kinase	P41241	--	0,016		1,70E-01	
EPB41L3	erythrocyte membrane protein band 4.1 like 3	Q9WV92	--	0,016		3,66E-01	
ERMP1	endoplasmic reticulum metallopeptidase 1	Q3UVK0	--	0,017		3,39E-01	
TPD52L2	tumor protein D52 like 2	Q9CYZ2	--	0,018		2,25E-02	
FAM129B	family with sequence similarity 129 member B	Q8R1F1	--	0,018		4,16E-01	
PDIA6	protein disulfide isomerase family A member 6	Q922R8	--	0,018		3,28E-01	
PSMC4	proteasome 26S subunit, ATPase 4	P54775	--	0,019		4,94E-02	
**RALA**	**RAS like proto-oncogene A**	P63321	--	0,02		7,47E-02	
RBMX	RNA binding motif protein, X-linked	Q9WV02	--	0,02		4,85E-02	
PNKP	polynucleotide kinase 3'-phosphatase	Q9JLV6	--	0,02		1,66E-01	
CLIP1	CAP-Gly domain containing linker protein 1	Q922J3	--	0,021		6,06E-01	
Ubap2l	ubiquitin-associated protein 2-like	Q80X50	--	0,023		2,41E-01	
DCAKD	dephospho-CoA kinase domain containing	Q8BHC4	--	0,023		1,25E-01	
**RAB9A**	**RAB9A, member RAS oncogene family**	Q9R0M6	--	0,024		3,38E-01	
DCTN4	dynactin subunit 4	Q8CBY8	--	0,024		1,46E-02	
COPG1	coatomer protein complex subunit gamma 1	Q9QZE5	Q9QZE5	0,024	-0,068	8,23E-03	4,47E-03
Nes	nestin	Q6P5H2	Q6P5H2	0,025	0,067	1,16E-01	1,95E-07
**ACTN4**	**actinin alpha 4**	P57780	--	0,025		3,01E-02	
CTSD	cathepsin D	P18242	P18242	0,025	0,123	4,62E-01	2,52E-01
ERBIN	erbb2 interacting protein	Q80TH2	--	0,026		2,28E-01	

IGSF10	immunoglobulin superfamily member 10	Q3V1M1	--	0,027		4,85E-02	
CLTC	clathrin heavy chain	Q68FD5	Q68FD5	0,028	-0,102	3,09E-01	4,83E-04
SH3GLB1	SH3 domain containing GRB2 like, endophilin B1	Q9JK48	--	0,028		3,37E-01	
LSP1	lymphocyte-specific protein 1	P19973	--	0,028		2,66E-01	
Dazap1	DAZ associated protein 1	Q9JII5	--	0,029		3,36E-01	
PDIA4	protein disulfide isomerase family A member 4	P08003	--	0,029		1,83E-03	
PPT2	palmitoyl-protein thioesterase 2	O35448	--	0,029		4,65E-01	
**IGF1R**	**insulin like growth factor 1 receptor**	Q60751	Q60751	0,03	0,202	1,63E-01	5,04E-01
ABRAXAS2	abraxas 2, BRISC complex subunit	Q3TCJ1	--	0,03		5,53E-01	
PELP1	proline, glutamate and leucine rich protein 1	Q9DBD5	--	0,03		2,74E-02	
**HSPA5**	**heat shock protein family A (Hsp70) member 5**	P20029	P20029	0,03	-0,051	5,81E-01	6,21E-02
BAG3	BCL2 associated athanogene 3	Q9JLV1	--	0,031		3,54E-01	
**MYO9B**	**myosin IXB**	Q9QY06	--	0,031		9,51E-02	
LMNA	lamin A/C	P48678	P48678	0,031	-0,019	2,25E-02	7,24E-02
CLIP2	CAP-Gly domain containing linker protein 2	Q9Z0H8	--	0,031		1,42E-02	
CSNK2A2	casein kinase 2 alpha 2	O54833	--	0,032		1,83E-01	
HADHA	hydroxyacyl-CoA dehydrogenase/3-ketoacyl-CoA thiolase/enoyl-CoA hydratase (trifunctional protein), alpha subunit	Q8BMS1	--	0,034		3,52E-08	
ELAVL1	ELAV like RNA binding protein 1	P70372	P70372	0,034	0,012	5,20E-02	5,39E-01
BRIX1	BRX1, biogenesis of ribosomes	Q9DCA5	Q9DCA5	0,034	-0,013	1,45E-03	2,19E-02
MMP14	matrix metallopeptidase 14	P53690	--	0,035		2,48E-01	
**CYCS**	**cytochrome c, somatic**	P62897	--	0,036		2,07E-01	
AP1M1	adaptor related protein complex 1 mu 1 subunit	P35585	--	0,036		6,05E-01	
PYGB	glycogen phosphorylase B	Q8CI94	Q8CI94	0,036	-0,193	2,31E-02	3,52E-02
DBN1	drebrin 1	Q9QXS6	--	0,037		2,35E-03	
**RPS12**	**ribosomal protein S12**	P63323	--	0,037		1,30E-02	
GLIPR2	GLI pathogenesis related 2	Q9CYL5	--	0,037		1,12E-01	
**EIF2S2**	**eukaryotic translation initiation factor 2 subunit beta**	Q99L45	--	0,037		7,05E-03	
FN1	fibronectin 1	P11276	P11276	0,037	-0,045	6,21E-02	4,98E-06
FAM98A	family with sequence similarity 98 member A	Q3TJZ6	--	0,039		6,05E-01	
SMARCC2	SWI/SNF related, matrix associated, actin dependent regulator of chromatin subfamily c member 2	Q6PDG5	--	0,039		1,26E-01	

GART	phosphoribosylglycinamide formyltransferase, phosphoribosylglycinamide synthetase, phosphoribosylaminoimidazole synthetase	Q64737	--	0,04		5,75E-01	
**RPL4**	**ribosomal protein L4**	Q9D8E6	Q9D8E6	0,04	0,197	6,66E-03	3,11E-03
PPP1R18	protein phosphatase 1 regulatory subunit 18	Q8BQ30	--	0,041		8,85E-02	
EGFR	epidermal growth factor receptor	Q01279	Q01279	0,041	0,069	4,64E-01	4,16E-01
HINT1	histidine triad nucleotide binding protein 1	P70349	--	0,042		4,96E-01	
CMPK1	cytidine/uridine monophosphate kinase 1	Q9DBP5	--	0,044		3,17E-02	
**HK2**	**hexokinase 2**	O08528	O08528	0,044	0,088	1,26E-01	8,40E-05
LEMD2	LEM domain containing 2	Q6DVA0	--	0,044		2,43E-02	
**ATP5O**	**ATP synthase, H+ transporting, mitochondrial F1 complex, O subunit**	Q9DB20	--	0,044		2,32E-01	
**PC**	**pyruvate carboxylase**	Q05920	--	0,045		2,32E-02	
FNDC3B	fibronectin type III domain containing 3B	Q6NWW9	--	0,045		8,74E-03	
**RHOJ**	**ras homolog family member J**	Q9ER71	--	0,046		5,96E-01	
**TRAP1**	**TNF receptor associated protein 1**	Q9CQN1	Q9CQN1	0,046	0,093	5,33E-01	5,65E-01
EPS15L1	epidermal growth factor receptor pathway substrate 15 like 1	Q60902	--	0,047		1,64E-01	
SCAMP3	secretory carrier membrane protein 3	O35609	--	0,049		8,62E-02	
MFGE8	milk fat globule-EGF factor 8 protein	P21956	--	0,049		2,12E-01	
PTK7	protein tyrosine kinase 7 (inactive)	Q8BKG3	Q8BKG3	0,049	0,014	4,92E-01	7,10E-03
EPRS	glutamyl-prolyl-tRNA synthetase	Q8CGC7	--	0,049		2,85E-02	
VCL	vinculin	Q64727	--	0,05		6,07E-02	
CEPT1	choline/ethanolamine phosphotransferase 1	Q8BGS7	--	0,05		3,21E-01	
COL12A1	collagen type XII alpha 1 chain	Q60847	Q60847	0,05	0,093	4,83E-01	9,55E-07
CEBPZ	CCAAT/enhancer binding protein zeta	P53569	P53569	0,05	-0,027	4,43E-01	5,82E-01
**HSP90B1**	**heat shock protein 90 beta family member 1**	P08113	--	0,051		7,54E-02	
ACP6	acid phosphatase 6, lysophosphatidic	Q8BP40	--	0,052		5,73E-01	
Sf1	splicing factor 1	Q64213	--	0,052		8,83E-05	
FABP3	fatty acid binding protein 3	P11404	--	0,053		2,34E-01	
COL18A1	collagen type XVIII alpha 1 chain	P39061	P39061	0,053	0,001	4,47E-01	3,56E-01
**RHOG**	**ras homolog family member G**	P84096	--	0,055		6,29E-02	
**ACO1**	**aconitase 1**	P28271	P28271	0,055	0,01	5,62E-01	2,37E-01
CNN2	calponin 2	Q08093	--	0,056		1,54E-02	

MYADM	myeloid associated differentiation marker	O35682	--	0,056		2,48E-03	
FAM120A	family with sequence similarity 120A	Q6A0A9	--	0,056		2,21E-04	
USP39	ubiquitin specific peptidase 39	Q3TIX9	--	0,056		2,00E-01	
**MYO5A**	**myosin VA**	Q99104	--	0,057		3,82E-02	
CALR	calreticulin	P14211	P14211	0,059	0,02	5,53E-01	2,37E-01
MCL1	MCL1, BCL2 family apoptosis regulator	P97287	--	0,06		4,92E-01	
CHCHD6	coiled-coil-helix-coiled-coil-helix domain containing 6	Q91VN4	--	0,061		5,50E-01	
**PLCB3**	**phospholipase C beta 3**	P51432	--	0,062		1,32E-01	
KANK2	KN motif and ankyrin repeat domains 2	Q8BX02	--	0,062		1,97E-02	
ESYT1	extended synaptotagmin 1	Q3U7R1	--	0,062		4,59E-01	
ISOC2	isochorismatase domain containing 2	P85094	--	0,063		1,67E-01	
CLPTM1	CLPTM1, transmembrane protein	Q8VBZ3	--	0,063		4,22E-02	
SYVN1	synoviolin 1	Q9DBY1	Q9DBY1	0,064	0,267	4,32E-01	5,79E-01
KIF2A	kinesin family member 2A	P28740	--	0,064		1,75E-01	
CLASP1	cytoplasmic linker associated protein 1	Q80TV8	--	0,065		3,94E-01	
Cdc42	cell division cycle 42	P60766	--	0,065		2,22E-01	
P4HB	prolyl 4-hydroxylase subunit beta	P09103	--	0,066		4,87E-01	
BCAP31	B-cell receptor-associated protein 31	Q61335	--	0,066		3,84E-02	
CCDC80	coiled-coil domain containing 80	Q8R2G6	--	0,066		2,86E-01	
VAT1	vesicle amine transport 1	Q62465	--	0,066		4,81E-02	
SPG21	SPG21, maspardin	Q9CQC8	--	0,067		2,27E-02	
**IMMT**	**inner membrane mitochondrial protein**	Q8CAQ8	--	0,067		3,20E-01	
DOCK7	dedicator of cytokinesis 7	Q8R1A4	--	0,067		3,55E-02	
DDX39A	DExD-box helicase 39A	Q8VDW0	--	0,068		3,18E-02	
**HSPA8**	**heat shock protein family A (Hsp70) member 8**	P63017	--	0,068		6,34E-03	
DAP3	death associated protein 3	Q9ER88	--	0,068		5,46E-01	
PPP1CA	protein phosphatase 1 catalytic subunit alpha	P62137	P62137	0,068	-0,33	6,38E-02	7,93E-04
AFG3L2	AFG3 like matrix AAA peptidase subunit 2	Q8JZQ2	--	0,069		1,33E-03	
PRKACB	protein kinase cAMP-activated catalytic subunit beta	P68181	--	0,069		1,05E-01	
GTF2I	general transcription factor IIi	Q9ESZ8	--	0,07		3,91E-01	
SPTBN1	spectrin beta, non-erythrocytic 1	Q62261	--	0,07		1,11E-02	
PSMA2	proteasome subunit alpha 2	P49722	--	0,071		4,38E-01	
**DNAJC1**	**DnaJ heat shock protein family (Hsp40) member C1**	Q61712	--	0,072		1,63E-02	
CD36	CD36 molecule	Q08857	--	0,073		4,08E-01	

CALU	calumenin	O35887	O35887	0,075	-0,011	1,53E-02	4,88E-02
**LAMB1**	**laminin subunit beta 1**	P02469	P02469	0,075	-0,047	1,62E-03	4,79E-02
GRWD1	glutamate rich WD repeat containing 1	Q810D6	--	0,076		1,17E-01	
MCFD2	multiple coagulation factor deficiency 2	Q8K5B2	--	0,076		1,35E-01	
**RAB11FIP**	**RAB11 family interacting protein 5**	Q8R361	--	0,076		3,48E-02	
**ATP5A1**	**ATP synthase, H+ transporting, mitochondrial F1 complex, alpha subunit 1, cardiac muscle**	Q03265	--	0,076		1,48E-02	
PHB	prohibitin	P67778	--	0,077		3,78E-02	
**ACTN1**	**actinin alpha 1**	Q7TPR4	Q7TPR4	0,079	-0,085	2,90E-02	1,12E-02
PLEC	plectin	Q9QXS1	Q9QXS1	0,082	0,072	1,11E-04	2,72E-02
SMARCA4	SWI/SNF related, matrix associated, actin dependent regulator of chromatin, subfamily a, member 4	Q3TKT4	Q3TKT4	0,082	-0,051	1,28E-01	2,23E-01
**MYL12A**	**myosin light chain 12A**	Q3THE2	--	0,083		5,72E-02	
PDLIM7	PDZ and LIM domain 7	Q3TJD7	--	0,084		1,67E-01	
DYNC1H1	dynein cytoplasmic 1 heavy chain 1	Q9JHU4	--	0,084		1,65E-06	
**FASN**	**fatty acid synthase**	P19096	P19096	0,085	-0,189	2,80E-10	1,39E-02
**NDUFB8**	**NADH:ubiquinone oxidoreductase subunit B8**	Q9D6J5	--	0,085		2,07E-01	
SRC	SRC proto-oncogene, non-receptor tyrosine kinase	P05480	P05480	0,085	0,074	3,26E-02	1,13E-01
YWHAZ	tyrosine 3-monooxygenase/tryptophan 5-monooxygenase activation protein zeta	P63101	--	0,085		3,60E-01	
**HSPA9**	**heat shock protein family A (Hsp70) member 9**	P38647	--	0,085		7,82E-06	
TGFB3	transforming growth factor beta 3	P17125	P17125	0,087	-0,528	2,21E-01	3,72E-01
TNS3	tensin 3	Q5SSZ5	Q5SSZ5	0,087	0,092	4,56E-02	5,34E-01
FLNA	filamin A	Q8BTM8	Q8BTM8	0,089	0,08	1,09E-02	1,83E-04
**NDUFB7**	**NADH:ubiquinone oxidoreductase subunit B7**	Q9CR61	--	0,09		5,40E-01	
PRNP	prion protein	P04925	--	0,09		2,21E-01	
PTPN23	protein tyrosine phosphatase, non-receptor type 23	Q6PB44	--	0,09		5,57E-01	
**MRPL14**	**mitochondrial ribosomal protein L14**	Q9D1I6	--	0,091		4,34E-01	
ERP29	endoplasmic reticulum protein 29	P57759	--	0,092		8,94E-03	
**PLSCR3**	**phospholipid scramblase 3**	Q9JIZ9	--	0,092		6,32E-02	
UGGT1	UDP-glucose glycoprotein glucosyltransferase 1	Q6P5E4	--	0,092		5,90E-01	
PRPF19	pre-mRNA processing factor 19	Q99KP6	--	0,092		1,10E-01	
**ACADS**	**acyl-CoA dehydrogenase, C-2 to C-3 short chain**	Q07417	--	0,093		4,24E-02	
CMC1	C-X9-C motif containing 1	Q9CPZ8	--	0,094		2,10E-01	
ECI2	enoyl-CoA delta isomerase 2	Q9WUR2	Q9WUR2	0,094	0,157	2,99E-02	2,87E-02

**AFAP1**	**actin filament associated protein 1**	Q80YS6	--	0,095		1,58E-01	
**GPX7**	**glutathione peroxidase 7**	Q99LJ6	--	0,096		3,06E-02	
**MDH2**	**malate dehydrogenase 2**	P08249	--	0,096		1,30E-02	
NUCB1	nucleobindin 1	Q02819	Q02819	0,096	-0,033	8,05E-02	1,80E-02
SLC25A3	solute carrier family 25 member 3	Q8VEM8	--	0,097		7,62E-03	
Nedd4	neural precursor cell expressed, developmentally down-regulated 4	P46935	--	0,098		5,73E-03	
TMLHE	trimethyllysine hydroxylase, epsilon	Q91ZE0	--	0,099		3,03E-01	
**PLA2G15**	**phospholipase A2 group XV**	Q8VEB4	--	0,1		1,38E-01	
YIPF4	Yip1 domain family member 4	Q8C407	--	0,101		1,49E-01	
**MT-CO2**	**cytochrome c oxidase subunit II**	P00405	--	0,102		8,89E-03	
ITGAV	integrin subunit alpha V	P43406	P43406	0,102	0,008	7,00E-02	5,00E-01
CSNK2A1	casein kinase 2 alpha 1	Q60737	--	0,104		3,07E-03	
**ALDH6A1**	**aldehyde dehydrogenase 6 family member A1**	Q9EQ20	--	0,108		6,94E-02	
**ACTA2**	**actin, alpha 2, smooth muscle, aorta**	P62737	--	0,108		8,99E-02	
S100A6	S100 calcium binding protein A6	P14069	--	0,109		3,94E-01	
AK1	adenylate kinase 1	Q9R0Y5	--	0,109		1,20E-01	
**NDUFA4**	**NDUFA4, mitochondrial complex associated**	Q62425	--	0,109		2,06E-02	
SLC25A22	solute carrier family 25 member 22	Q9D6M3	--	0,111		3,55E-01	
MVP	major vault protein	Q9EQK5	--	0,111		9,65E-03	
**NDUFB3**	**NADH:ubiquinone oxidoreductase subunit B3**	Q9CQZ6	--	0,112		5,05E-02	
**NDUFS1**	**NADH:ubiquinone oxidoreductase core subunit S1**	Q91VD9	--	0,112		1,82E-02	
IPO5	importin 5	Q8BKC5	--	0,112		2,63E-02	
**HSPB1**	**heat shock protein family B (small) member 1**	P14602	--	0,115		8,88E-02	
CD44	CD44 molecule (Indian blood group)	P15379	P15379	0,115	0,041	2,00E-01	4,61E-01
SLC25A5	solute carrier family 25 member 5	P51881	--	0,115		5,60E-01	
**RAC1**	**ras-related C3 botulinum toxin substrate 1 (rho family, small GTP binding protein Rac1)**	P63001	--	0,116		1,80E-01	
GCAT	glycine C-acetyltransferase	O88986	--	0,117		2,06E-01	
IMPAD1	inositol monophosphatase domain containing 1	Q80V26	--	0,117		8,02E-03	
MAN2A1	mannosidase alpha class 2A member 1	P27046	--	0,118		2,70E-02	
GNAI2	G protein subunit alpha i2	P08752	--	0,119		8,77E-02	
RTN4	reticulon 4	Q99P72	--	0,12		5,95E-05	
1700047I1	family with sequence similarity 177, member A	Q8BR63	--	0,12		3,67E-02	
RELA	RELA proto-oncogene, NF-kB subunit	Q04207	--	0,12		3,95E-01	
PPIB	peptidylprolyl isomerase B	P24369	--	0,122		2,95E-01	

**EIF3M**	**eukaryotic translation initiation factor 3 subunit M**	Q99JX4	--	0,122		2,67E-02	
TMOD3	tropomodulin 3	Q9JHJ0	--	0,122		1,08E-01	
ANXA2	annexin A2	P07356	P07356	0,123	-0,185	4,55E-01	8,43E-03
**POR**	**cytochrome p450 oxidoreductase**	P37040	P37040	0,123	0,093	3,89E-02	5,87E-01
TLN1	talin 1	P26039	--	0,124		2,49E-01	
VCP	valosin containing protein	Q01853	Q01853	0,124	0,091	2,53E-02	4,06E-02
PDLIM1	PDZ and LIM domain 1	O70400	--	0,125		3,07E-03	
P4HA1	prolyl 4-hydroxylase subunit alpha 1	Q60715	--	0,125		4,03E-01	
AP1B1	adaptor related protein complex 1 beta 1 subunit	O35643	--	0,125		2,30E-02	
PHLDA3	pleckstrin homology like domain family A member 3	Q9WV95	--	0,126		1,86E-01	
**MYH11**	**myosin heavy chain 11**	O08638	--	0,127		2,66E-01	
**PRDX5**	**peroxiredoxin 5**	P99029	--	0,127		2,97E-01	
AP2A1	adaptor related protein complex 2 alpha 1 subunit	P17426	P17426	0,127	0,186	5,37E-02	2,32E-01
**ACAT1**	**acetyl-CoA acetyltransferase 1**	Q8QZT1	--	0,129		1,39E-02	
NAXE	NAD(P)HX epimerase	Q8K4Z3	--	0,129		1,74E-01	
**ATP6V0C**	**ATPase H+ transporting V0 subunit c**	P63082	--	0,13		1,70E-02	
CHCHD4	coiled-coil-helix-coiled-coil-helix domain containing 4	Q8VEA4	--	0,13		6,62E-02	
GTPBP10	GTP binding protein 10	Q8K013	--	0,131		4,11E-01	
FZD7	frizzled class receptor 7	Q61090	--	0,131		2,04E-01	
MAP2K1	mitogen-activated protein kinase kinase 1	P31938	P31938	0,134	0,122	3,29E-01	5,33E-01
NAPA	NSF attachment protein alpha	Q9DB05	--	0,134		2,39E-02	
NCSTN	nicastrin	P57716	--	0,134		3,39E-02	
**PLBD2**	**phospholipase B domain containing 2**	Q3TCN2	--	0,135		4,41E-01	
PSEN1	presenilin 1	P49769	P49769	0,135	0,202	4,01E-01	1,41E-01
SPTLC1	serine palmitoyltransferase long chain base subunit 1	O35704	--	0,138		3,25E-02	
ECH1	enoyl-CoA hydratase 1	O35459	--	0,138		2,40E-01	
COL7A1	collagen type VII alpha 1 chain	Q63870	--	0,138		2,29E-01	
HSD17B12	hydroxysteroid 17-beta dehydrogenase 12	O70503	--	0,139		3,17E-01	
**PLPP3**	**phospholipid phosphatase 3**	Q99JY8	Q99JY8	0,139	0,306	2,06E-02	6,75E-02
RAI14	retinoic acid induced 14	Q9EP71	--	0,14		6,45E-05	
LAMTOR1	late endosomal/lysosomal adaptor, MAPK and MTOR activator 1	Q9CQ22	--	0,14		1,38E-02	
SLC9A3R2	SLC9A3 regulator 2	Q9JHL1	--	0,14		5,70E-01	
DDX46	DEAD-box helicase 46	Q569Z5	--	0,14		2,04E-01	
ITGB6	integrin subunit beta 6	Q9Z0T9	Q9Z0T9	0,141	0,077	3,84E-01	5,92E-01
RDH14	retinol dehydrogenase 14 (all-trans/9-cis/11-cis)	Q9ERI6	--	0,141		3,10E-02	

ZMPSTE24	zinc metallopeptidase STE24	Q80W54	--	0,141		2,76E-01	
HYOU1	hypoxia up-regulated 1	Q9JKR6	--	0,142		4,07E-03	
**MYL6**	**myosin light chain 6**	Q60605	--	0,143		9,80E-03	
GNG12	G protein subunit gamma 12	Q9DAS9	--	0,143		3,34E-02	
JUP	junction plakoglobin	Q02257	--	0,144		8,02E-04	
MAVS	mitochondrial antiviral signaling protein	Q8VCF0	--	0,144		5,99E-01	
PTPRA	protein tyrosine phosphatase, receptor type A	P18052	--	0,144		4,40E-01	
RAB18	RAB18, member RAS oncogene family	P35293	--	0,144		2,82E-02	
**MTOR**	**mechanistic target of rapamycin**	Q9JLN9	Q9JLN9	0,145	0,185	3,48E-01	5,61E-01
SNAP47	synaptosome associated protein 47	Q8R570	--	0,146		3,19E-01	
FHL2	four and a half LIM domains 2	O70433	--	0,146		1,53E-03	
PIEZO1	piezo type mechanosensitive ion channel component 1	E2JF22	--	0,146		1,53E-01	
DLST	dihydrolipoamide S-succinyltransferase	Q9D2G2	--	0,148		2,19E-02	
MOV10	Mov10 RISC complex RNA helicase	P23249	--	0,149		4,41E-01	
ALCAM	activated leukocyte cell adhesion molecule	Q61490	--	0,149		4,46E-03	
COMTD1	catechol-O-methyltransferase domain containing 1	Q8BIG7	--	0,15		1,57E-02	
TMEM30A	transmembrane protein 30A	Q8VEK0	--	0,15		2,67E-03	
**MYH10**	**myosin heavy chain 10**	Q61879	--	0,15		3,50E-02	
FECH	ferrochelatase	P22315	P22315	0,151	0,235	4,76E-01	3,29E-01
SPRYD4	SPRY domain containing 4	Q91WK1	--	0,152		1,36E-03	
**LAMC1**	**laminin subunit gamma 1**	P02468	--	0,152		1,89E-01	
DLAT	dihydrolipoamide S-acetyltransferase	Q8BMF4	Q8BMF4	0,152	0,136	1,09E-02	6,23E-03
RAB11A	RAB11A, member RAS oncogene family	P62492	--	0,153		1,83E-02	
F2R	coagulation factor II thrombin receptor	P30558	P30558	0,153	-0,027	3,19E-01	3,51E-01
CAVIN1	caveolae associated protein 1	O54724	--	0,154		4,27E-04	
NOL7	nucleolar protein 7	Q9D7Z3	--	0,155		2,64E-01	
CAND1	cullin associated and neddylation dissociated 1	Q6ZQ38	--	0,155		3,65E-04	
PLEKHF1	pleckstrin homology and FYVE domain containing 1	Q3TB82	--	0,156		3,18E-01	
STK25	serine/threonine kinase 25	Q9Z2W1	--	0,156		1,64E-01	
CKAP4	cytoskeleton associated protein 4	Q8BMK4	--	0,156		5,78E-01	
ATP2A2	ATPase sarcoplasmic/endoplasmic reticulum Ca2+ transporting 2	O55143	O55143	0,156	0,132	1,10E-05	3,85E-01
SLC25A20	solute carrier family 25 member 20	Q9Z2Z6	--	0,157		2,93E-03	
RHOA	ras homolog family member A	Q9QUI0	Q9QUI0	0,158	0,131	5,89E-01	5,87E-01
**PDHB**	**pyruvate dehydrogenase (lipoamide) beta**	Q9D051	--	0,16		2,78E-01	

STX8	syntaxin 8	O88983	--	0,16		1,85E-02	
LAMTOR2	late endosomal/lysosomal adaptor, MAPK and MTOR activator 2	Q9JHS3	--	0,161		1,02E-01	
IST1	IST1, ESCRT-III associated factor	Q9CX00	Q9CX00	0,162	0,314	2,02E-02	1,14E-02
NCS1	neuronal calcium sensor 1	Q8BNY6	--	0,162		2,54E-02	
ERLEC1	endoplasmic reticulum lectin 1	Q8VEH8	--	0,163		2,13E-01	
**GLRX3**	**glutaredoxin 3**	Q9CQM9	--	0,164		4,50E-01	
FTL	ferritin light chain	P29391	--	0,166		1,04E-02	
GSTO1	glutathione S-transferase omega 1	O09131	--	0,167		2,45E-01	
SEC63	SEC63 homolog, protein translocation regulator	Q8VHE0	--	0,167		3,94E-03	
HOMER3	homer scaffolding protein 3	Q99JP6	--	0,167		7,19E-03	
RRAS2	related RAS viral (r-ras) oncogene homolog 2	P62071	--	0,167		1,99E-02	
MYDGF	myeloid derived growth factor	Q9CPT4	--	0,167		5,31E-01	
PTK2	protein tyrosine kinase 2	P34152	P34152	0,167	0,391	7,05E-02	2,14E-01
SPARC	secreted protein acidic and cysteine rich	P07214	P07214	0,169	-0,158	2,62E-01	1,27E-01
**UQCR11**	**ubiquinol-cytochrome c reductase, complex III subunit XI**	Q9CPX8	--	0,17		2,42E-01	
**UQCR11**	**ubiquinol-cytochrome c reductase, complex III subunit XI**	Q9CPX8	--	0,17		2,42E-01	
**UQCR11**	**ubiquinol-cytochrome c reductase, complex III subunit XI**	Q9CPX8	--	0,17		2,42E-01	
**UQCR11**	**ubiquinol-cytochrome c reductase, complex III subunit XI**	Q9CPX8	--	0,17		2,42E-01	
**UQCR11**	**ubiquinol-cytochrome c reductase, complex III subunit XI**	Q9CPX8	--	0,17		2,42E-01	
AGA	aspartylglucosaminidase	Q64191	--	0,17		4,96E-03	
**CAT**	**catalase**	P24270	P24270	0,17	-0,021	2,13E-01	2,26E-01
SYNPO2	synaptopodin 2	Q91YE8	Q91YE8	0,172	0,098	5,80E-03	7,62E-03
CHCHD3	coiled-coil-helix-coiled-coil-helix domain containing 3	Q9CRB9	--	0,173		1,81E-03	
FTH1	ferritin heavy chain 1	P09528	--	0,173		7,67E-02	
STOM	stomatin	P54116	P54116	0,176	0,23	1,43E-02	1,10E-02
FLOT2	flotillin 2	Q60634	--	0,178		3,89E-02	
PRKAR2A	protein kinase cAMP-dependent type II regulatory subunit alpha	P12367	--	0,178		2,26E-02	
HINT2	histidine triad nucleotide binding protein 2	Q9D0S9	--	0,179		1,01E-02	
ITGB1	integrin subunit beta 1	P09055	P09055	0,179	-0,021	1,97E-03	1,97E-01
MMP2	matrix metallopeptidase 2	P33434	--	0,179		6,21E-02	
SNAP23	synaptosome associated protein 23	O09044	--	0,18		6,29E-02	
SFXN3	sideroflexin 3	Q91V61	--	0,18		1,46E-02	
STAM2	signal transducing adaptor molecule 2	O88811	--	0,181		9,39E-03	
RAP2B	RAP2B, member of RAS oncogene family	P61226	--	0,182		4,47E-02	
SORBS1	sorbin and SH3 domain containing 1	Q62417	--	0,182		1,17E-01	

Ppp1r12b	protein phosphatase 1, regulatory (inhibitor) subunit 12B	Q8BG95	Q8BG95	0,183	0,175	2,99E-02	3,34E-02
**IGF2BP1**	**insulin like growth factor 2 mRNA binding protein 1**	O88477	O88477	0,183	0,062	2,80E-01	5,94E-01
**NDUFS2**	**NADH:ubiquinone oxidoreductase core subunit S2**	Q91WD5	--	0,184		6,07E-01	
SYNPO	synaptopodin	Q8CC35	--	0,184		6,04E-01	
UCK2	uridine-cytidine kinase 2	Q99PM9	--	0,184		4,79E-01	
ENG	endoglin	Q63961	--	0,184		5,77E-02	
**ACSF2**	**acyl-CoA synthetase family member 2**	Q8VCW8	--	0,185		2,13E-02	
**NDUFA3**	**NADH:ubiquinone oxidoreductase subunit A3**	Q9CQ91	--	0,185		4,65E-01	
**MYH9**	**myosin heavy chain 9**	Q8VDD5	--	0,185		2,79E-06	
PTGS2	prostaglandin-endoperoxide synthase 2	Q05769	--	0,186		2,94E-04	
RDH13	retinol dehydrogenase 13	Q8CEE7	Q8CEE7	0,186	0,313	2,13E-01	2,08E-02
CCDC127	coiled-coil domain containing 127	Q3TC33	--	0,186		8,11E-02	
HIGD1A	HIG1 hypoxia inducible domain family member 1A	Q9JLR9	--	0,187		2,91E-01	
RAB5A	RAB5A, member RAS oncogene family	Q9CQD1	--	0,189		1,07E-02	
INHBA	inhibin beta A subunit	Q04998	--	0,192		2,62E-02	
**ACTB**	**actin beta**	P60710	P60710	0,193	0,043	5,82E-02	4,01E-01
RAB22A	RAB22A, member RAS oncogene family	P35285	--	0,193		2,73E-02	
Crip2	cysteine rich protein 2	Q9DCT8	--	0,194		1,41E-02	
MGEA5	meningioma expressed antigen 5 (hyaluronidase)	Q9EQQ9	Q9EQQ9	0,195	0,193	4,87E-01	5,76E-01
PSAP	prosaposin	Q61207	--	0,195		5,31E-02	
RAB1B	RAB1B, member RAS oncogene family	Q9D1G1	Q9D1G1	0,196	0,278	4,47E-01	1,33E-01
ERLIN2	ER lipid raft associated 2	Q8BFZ9	--	0,196		1,91E-03	
ITGA6	integrin subunit alpha 6	Q61739	--	0,196		1,85E-04	
TRIM16	tripartite motif containing 16	Q99PP9	--	0,197		4,44E-02	
**MRPS23**	**mitochondrial ribosomal protein S23**	Q8VE22	--	0,198		1,37E-01	
CTNNB1	catenin beta 1	Q02248	--	0,198		3,83E-01	
C21orf33/I	chromosome 21 open reading frame 33	Q9D172	--	0,199		1,04E-02	
MUT	methylmalonyl-CoA mutase	P16332	--	0,201		1,66E-01	
AKT1S1	AKT1 substrate 1	Q9D1F4	--	0,201		1,80E-01	
MPP1	membrane palmitoylated protein 1	P70290	--	0,201		3,91E-03	
ECE1	endothelin converting enzyme 1	Q4PZA2	Q4PZA2	0,207	0,169	9,26E-03	5,82E-01
LETM1	leucine zipper and EF-hand containing transmembrane protein 1	Q9Z2I0	--	0,207		9,81E-04	
GNAO1	G protein subunit alpha o1	P18872	--	0,208		1,65E-02	

**ATP5D**	**ATP synthase, H+ transporting, mitochondrial F1 complex, delta subunit**	Q9D3D9	--	0,209		6,13E-02	
CTNNA1	catenin alpha 1	P26231	--	0,209		2,98E-04	
PRKCSH	protein kinase C substrate 80K-H	O08795	--	0,214		8,10E-05	
C17orf62	chromosome 17 open reading frame 62	Q3TYS2	--	0,214		4,82E-01	
CTGF	connective tissue growth factor	P29268	P29268	0,214	-0,058	1,88E-06	5,73E-02
SEC11A	SEC11 homolog A, signal peptidase complex subunit	Q9R0P6	--	0,215		5,68E-01	
SLC35B2	solute carrier family 35 member B2	Q91ZN5	--	0,216		1,23E-01	
**VDAC3**	**voltage dependent anion channel 3**	Q60931	--	0,216		1,70E-02	
STX7	syntaxin 7	O70439	O70439	0,216	0,129	2,46E-02	4,85E-02
NCLN	nicalin	Q8VCM8	--	0,216		4,48E-01	
ATP1B1	ATPase Na+/K+ transporting subunit beta 1	P14094	--	0,217		9,88E-03	
TUFM	Tu translation elongation factor, mitochondrial	Q8BFR5	--	0,217		2,40E-03	
HLA-A	major histocompatibility complex, class I, A	P01899	--	0,217		6,87E-05	
**ACADSB**	**acyl-CoA dehydrogenase, short/branched chain**	Q9DBL1	--	0,217		4,21E-02	
EEF1G	eukaryotic translation elongation factor 1 gamma	Q9D8N0	Q9D8N0	0,218	0,455	2,08E-01	9,07E-03
HBA1/HBA	hemoglobin subunit alpha 2	P01942	--	0,219		2,19E-01	
GNS	glucosamine (N-acetyl)-6-sulfatase	Q8BFR4	--	0,219		8,45E-03	
ECM1	extracellular matrix protein 1	Q61508	--	0,22		5,26E-03	
ITGB5	integrin subunit beta 5	O70309	--	0,22		3,08E-02	
SLC33A1	solute carrier family 33 member 1	Q99J27	--	0,222		1,50E-02	
**SIRT2**	**sirtuin 2**	Q8VDQ8	--	0,223		5,18E-03	
COPS3	COP9 signalosome subunit 3	O88543	--	0,223		5,19E-03	
**ACOX3**	**acyl-CoA oxidase 3, pristanoyl**	Q9EPL9	--	0,223		1,02E-02	
GNA11	G protein subunit alpha 11	P21278	--	0,223		2,75E-01	
FLNB	filamin B	Q80X90	--	0,224		3,19E-09	
DGKE	diacylglycerol kinase epsilon	Q9R1C6	--	0,225		2,41E-01	
**ATP6AP1**	**ATPase H+ transporting accessory protein 1**	Q9R1Q9	--	0,226		1,61E-02	
NOMO1 (ir	NODAL modulator 1	Q6GQT9	--	0,226		3,07E-03	
MTDH	metadherin	Q80WJ7	Q80WJ7	0,227	0,355	2,26E-02	1,61E-02
PIGK	phosphatidylinositol glycan anchor biosynthesis class K	Q9CXY9	--	0,227		4,46E-02	
ARL5A	ADP ribosylation factor like GTPase 5A	Q80ZU0	--	0,227		2,05E-02	
VWA8	von Willebrand factor A domain containing 8	Q8CC88	--	0,229		4,01E-02	
PDLIM5	PDZ and LIM domain 5	Q8CI51	--	0,23		1,19E-01	

SPTLC2	serine palmitoyltransferase long chain base subunit 2	P97363	--	0,231		1,14E-01	
SLC9A3R1	SLC9A3 regulator 1	P70441	--	0,233		2,77E-01	
AMPD3	adenosine monophosphate deaminase 3	O08739	--	0,233		2,97E-01	
GPC4	glypican 4	P51655	--	0,233		7,14E-05	
**CYC1**	**cytochrome c1**	Q9D0M3	--	0,233		3,36E-05	
GBA	glucosylceramidase beta	P17439	--	0,237		1,06E-03	
RAB21	RAB21, member RAS oncogene family	P35282	--	0,237		1,70E-03	
MFN2	mitofusin 2	Q80U63	--	0,238		3,49E-02	
SNAPIN	SNAP associated protein	Q9Z266	--	0,238		1,41E-02	
PRKCA	protein kinase C alpha	P20444	P20444	0,241	-0,007	5,04E-01	3,13E-01
**NDUFB5**	**NADH:ubiquinone oxidoreductase subunit B5**	Q9CQH3	--	0,241		4,29E-01	
ENDOD1	endonuclease domain containing 1	Q8C522	--	0,241		4,57E-02	
ITGA1	integrin subunit alpha 1	Q3V3R4	--	0,241		1,36E-02	
**IGFBP5**	**insulin like growth factor binding protein 5**	Q07079	Q07079	0,242	0,089	5,01E-01	1,93E-01
**BAK1**	**BCL2 antagonist/killer 1**	O08734	--	0,243		1,25E-02	
SQOR	sulfide quinone oxidoreductase	Q9R112	--	0,244		1,18E-02	
LONP1	lon peptidase 1, mitochondrial	Q8CGK3	Q8CGK3	0,245	0,077	3,70E-01	5,49E-01
PRPF8	pre-mRNA processing factor 8	Q99PV0	Q99PV0	0,246	0,262	1,53E-03	4,17E-02
DBT	dihydrolipoamide branched chain transacylase E2	P53395	--	0,246		3,88E-03	
SERPINC1	serpin family C member 1	P32261	P32261	0,247	0,09	2,12E-02	5,57E-01
DNAJB6	DnaJ heat shock protein family (Hsp40) member B6	O54946	--	0,247		9,65E-02	
**ALDH2**	**aldehyde dehydrogenase 2 family (mitochondrial)**	P47738	P47738	0,248	0,093	4,00E-03	5,19E-01
PML	promyelocytic leukemia	Q60953	Q60953	0,248	-0,004	4,99E-01	4,79E-01
VPS39	VPS39, HOPS complex subunit	Q8R5L3	--	0,25		1,12E-02	
SCARB1	scavenger receptor class B member 1	Q61009	Q61009	0,25	0,515	8,74E-03	4,80E-01
LRPAP1	LDL receptor related protein associated protein 1	P55302	P55302	0,251	0,014	2,66E-01	4,91E-01
POGLUT1	protein O-glucosyltransferase 1	Q8BYB9	--	0,252		2,55E-04	
DAD1	defender against cell death 1	P61804	--	0,252		9,94E-03	
TAF4B	TATA-box binding protein associated factor 4b	G5E8Z2	--	0,252		2,84E-01	
**GLRX5**	**glutaredoxin 5**	Q80Y14	--	0,253		9,68E-03	
**ATP6V1E1**	**ATPase H+ transporting V1 subunit E1**	P50518	--	0,253		4,49E-02	
APP	amyloid beta precursor protein	P12023	P12023	0,253	-0,145	5,73E-01	1,83E-02
ATL1	atlastin GTPase 1	Q8BH66	--	0,254		4,38E-02	
PURA	purine rich element binding protein A	P42669	--	0,254		5,19E-02	
SH3GLB2	SH3 domain containing GRB2 like, endophilin B2	Q8R3V5	--	0,255		1,20E-01	

TMED10	transmembrane p24 trafficking protein 10	Q9D1D4	--	0,255		5,25E-03	
SERPINF1	serpin family F member 1	P97298	--	0,256		3,80E-01	
DYSF	dysferlin	Q9ESD7	Q9ESD7	0,258	0,348	6,56E-05	6,18E-02
LIMA1	LIM domain and actin binding 1	Q9ERG0	--	0,259		5,97E-06	
DES	desmin	P31001	P31001	0,259	0,291	1,11E-02	1,71E-06
**ACADVL**	**acyl-CoA dehydrogenase, very long chain**	P50544	P50544	0,259	0,101	1,69E-06	1,96E-04
**COX7A2**	**cytochrome c oxidase subunit 7A2**	P48771	--	0,259		7,48E-03	
CKAP5	cytoskeleton associated protein 5	A2AGT5	--	0,259		1,62E-01	
USMG5	up-regulated during skeletal muscle growth 5 homolog (mouse)	Q78IK2	--	0,261		4,22E-02	
PTTG1IP	PTTG1 interacting protein	Q8R143	--	0,262		8,94E-02	
C1QTNF5	C1q and TNF related 5	Q8K479	--	0,262		2,51E-02	
ICMT	isoprenylcysteine carboxyl methyltransferase	Q9EQK7	--	0,264		3,46E-01	
ETFDH	electron transfer flavoprotein dehydrogenase	Q921G7	--	0,264		4,47E-01	
**NDUFAB1**	**NADH:ubiquinone oxidoreductase subunit AB1**	Q9CR21	Q9CR21	0,264	-0,34	3,25E-01	3,18E-01
TGFB2	transforming growth factor beta 2	P27090	P27090	0,268	0,125	6,01E-01	2,44E-01
**TMBIM6**	**transmembrane BAX inhibitor motif containing 6**	Q9D2C7	--	0,272		5,38E-01	
TOR1B	torsin family 1 member B	Q9ER41	--	0,273		4,33E-01	
**MYO1E**	**myosin IE**	E9Q634	E9Q634	0,275	0,405	8,45E-02	4,35E-03
P2RX4	purinergic receptor P2X 4	Q9JJX6	--	0,276		4,02E-02	
TSPAN3	tetraspanin 3	Q9QY33	--	0,277		4,73E-02	
RAB5C	RAB5C, member RAS oncogene family	P35278	--	0,277		4,35E-02	
**GPD2**	**glycerol-3-phosphate dehydrogenase 2**	Q64521	--	0,279		2,41E-02	
UBXN4	UBX domain protein 4	Q8VCH8	--	0,28		2,26E-02	
**DNAJC8**	**DnaJ heat shock protein family (Hsp40) member C8**	Q6NZB0	--	0,281		1,41E-01	
TMCC3	transmembrane and coiled-coil domain family 3	Q8R310	--	0,283		1,37E-01	
**VDAC2**	**voltage dependent anion channel 2**	Q60930	--	0,288		5,19E-03	
HM13	histocompatibility minor 13	Q9D8V0	Q9D8V0	0,288	0,277	2,28E-02	2,58E-01
PPP3R1	protein phosphatase 3 regulatory subunit B, alpha	Q63810	--	0,29		3,40E-02	
**ACOT13**	**acyl-CoA thioesterase 13**	Q9CQR4	--	0,291		2,45E-03	
PDLIM2	PDZ and LIM domain 2	Q8R1G6	Q8R1G6	0,291	0,313	1,67E-02	5,91E-03
GNAQ	G protein subunit alpha q	P21279	--	0,296		1,26E-01	
BMPR2	bone morphogenetic protein receptor type 2	O35607	--	0,297		2,47E-01	
**TMBIM1**	**transmembrane BAX inhibitor motif containing 1**	Q8BJZ3	--	0,298		3,74E-02	

**ATP5J**	**ATP synthase, H+ transporting, mitochondrial Fo complex subunit F6**	P97450	--	0,299		4,11E-02	
**ACOX1**	**acyl-CoA oxidase 1**	Q9R0H0	Q9R0H0	0,299	-0,061	6,05E-01	2,21E-01
ATG7	autophagy related 7	Q9D906	--	0,301		4,90E-02	
FUNDC1	FUN14 domain containing 1	Q9DB70	--	0,302		6,34E-02	
**ALDH4A1**	**aldehyde dehydrogenase 4 family member A1**	Q8CHT0	Q8CHT0	0,302	0,274	2,83E-04	4,24E-02
**HK1**	**hexokinase 1**	P17710	--	0,305		7,36E-02	
NPC1	NPC intracellular cholesterol transporter 1	O35604	--	0,306		7,62E-02	
SGPL1	sphingosine-1-phosphate lyase 1	Q8R0X7	--	0,306		8,55E-04	
FLRT2	fibronectin leucine rich transmembrane protein 2	Q8BLU0	--	0,306		3,82E-05	
HIP1	huntingtin interacting protein 1	Q8VD75	Q8VD75	0,307	0,111	3,61E-04	2,28E-03
ITGA3	integrin subunit alpha 3	Q62470	--	0,312		1,91E-01	
PBXIP1	PBX homeobox interacting protein 1	Q3TVI8	--	0,312		1,33E-04	
**DNAJB12**	**DnaJ heat shock protein family (Hsp40) member B12**	Q9QYI4	--	0,313		4,81E-01	
**HTRA1**	**HtrA serine peptidase 1**	Q9R118	--	0,315		4,82E-02	
**HADHB**	**hydroxyacyl-CoA dehydrogenase/3-ketoacyl-CoA thiolase/enoyl-CoA hydratase (trifunctional protein), beta subunit**	Q99JY0	--	0,315		4,80E-02	
HTATIP2	HIV-1 Tat interactive protein 2	Q9Z2G9	--	0,316		3,22E-02	
ADAM9	ADAM metallopeptidase domain 9	Q61072	--	0,318		1,19E-03	
VTI1B	vesicle transport through interaction with t-SNAREs 1B	O88384	--	0,319		5,95E-03	
**FIS1**	**fission, mitochondrial 1**	Q9CQ92	--	0,321		1,01E-01	
**NDUFS6**	**NADH:ubiquinone oxidoreductase subunit S6**	P52503	--	0,321		2,17E-02	
EFNB2	ephrin B2	P52800	--	0,324		9,59E-02	
**RAB7A**	**RAB7A, member RAS oncogene family**	P51150	--	0,324		4,69E-06	
ESYT2	extended synaptotagmin 2	Q3TZZ7	--	0,324		3,85E-05	
FUT8	fucosyltransferase 8	Q9WTS2	--	0,325		1,05E-01	
SLMAP	sarcolemma associated protein	Q3URD3	--	0,326		4,67E-03	
**ATP6V1C1**	**ATPase H+ transporting V1 subunit C1**	Q9Z1G3	--	0,329		1,43E-05	
PTGES2	prostaglandin E synthase 2	Q8BWM0	--	0,331		1,10E-02	
ILVBL	ilvB acetolactate synthase like	Q8BU33	--	0,332		4,91E-05	
TSPAN6	tetraspanin 6	O70401	--	0,334		1,97E-02	
RAB32	RAB32, member RAS oncogene family	Q9CZE3	--	0,336		9,62E-03	
IVD	isovaleryl-CoA dehydrogenase	Q9JHI5	--	0,337		2,60E-02	
BCAS2	BCAS2, pre-mRNA processing factor	Q9D287	--	0,338		1,93E-01	
THBS1	thrombospondin 1	P35441	--	0,339		8,48E-12	
SVIL	supervillin	Q8K4L3	--	0,339		4,72E-02	

**TIMM22**	**translocase of inner mitochondrial membrane 22**	Q9CQ85	--	0,34		2,99E-03	
HPCAL1	hippocalcin like 1	P62748	--	0,341		1,70E-03	
PURB	purine rich element binding protein B	O35295	--	0,342		1,60E-01	
PTPRZ1	protein tyrosine phosphatase, receptor type Z1	B9EKR1	--	0,343		1,91E-01	
**PMPCB**	**peptidase, mitochondrial processing beta subunit**	Q9CXT8	--	0,346		2,75E-02	
EFEMP2	EGF containing fibulin like extracellular matrix protein 2	Q9WVJ9	--	0,347		3,11E-02	
**ATP6V0A2**	**ATPase H+ transporting V0 subunit a2**	P15920	--	0,348		9,41E-03	
LMF1	lipase maturation factor 1	Q3U3R4	--	0,351		2,40E-03	
CPNE2	copine 2	P59108	--	0,353		7,87E-02	
CCND1	cyclin D1	P25322	P25322	0,36	0,491	5,73E-03	6,89E-02
HMOX1	heme oxygenase 1	P14901	P14901	0,363	0,078	2,22E-01	4,34E-02
SNF8	SNF8, ESCRT-II complex subunit	Q9CZ28	--	0,365		3,31E-01	
PAWR	pro-apoptotic WT1 regulator	Q925B0	--	0,366		7,24E-05	
**RIPK1**	**receptor interacting serine/threonine kinase 1**	Q60855	--	0,367		2,96E-02	
STIM1	stromal interaction molecule 1	P70302	--	0,369		5,58E-01	
CDH2	cadherin 2	P15116	--	0,369		1,13E-08	
CHST14	carbohydrate sulfotransferase 14	Q80V53	--	0,37		4,97E-02	
MPDU1	mannose-P-dolichol utilization defect 1	Q9R0Q9	--	0,372		3,26E-02	
MAOA	monoamine oxidase A	Q64133	--	0,374		2,18E-06	
EPHA2	EPH receptor A2	Q03145	--	0,377		4,99E-04	
PTGFRN	prostaglandin F2 receptor inhibitor	Q9WV91	--	0,385		1,58E-02	
**MYH14**	**myosin heavy chain 14**	Q6URW6	--	0,386		2,07E-03	
GPRC5A	G protein-coupled receptor class C group 5 member A	Q8BHL4	Q8BHL4	0,386	0,185	1,51E-02	3,95E-02
SYNJ2BP	synaptojanin 2 binding protein	Q9D6K5	--	0,387		9,85E-02	
**ALDH3A2**	**aldehyde dehydrogenase 3 family member A2**	P47740	P47740	0,388	0,203	1,81E-02	1,97E-01
TGM2	transglutaminase 2	P21981	--	0,39		1,75E-06	
CTNNA2	catenin alpha 2	Q61301	--	0,394		2,80E-02	
SORBS2	sorbin and SH3 domain containing 2	Q3UTJ2	--	0,395		1,03E-05	
**IGFBP2**	**insulin like growth factor binding protein 2**	P47877	--	0,395		1,04E-05	
RNF13	ring finger protein 13	O54965	--	0,395		3,69E-03	
MECP2	methyl-CpG binding protein 2	Q9Z2D6	--	0,395		5,00E-01	
NINJ1	ninjurin 1	O70131	--	0,401		6,30E-02	
TIMP3	TIMP metallopeptidase inhibitor 3	P39876	P39876	0,404	0,024	1,62E-02	2,65E-02
CD9	CD9 molecule	P40240	--	0,407		2,09E-01	
**ALDH7A1**	**aldehyde dehydrogenase 7 family member A1**	Q9DBF1	--	0,411		3,51E-01	
THBD	thrombomodulin	P15306	--	0,411		5,52E-04	
RHOQ	ras homolog family member Q	Q8R527	--	0,417		1,87E-02	

ITIH4	inter-alpha-trypsin inhibitor heavy chain family member 4	A6X935	--	0,421		4,33E-01	
CNBP	CCHC-type zinc finger nucleic acid binding protein	P53996	--	0,425		3,23E-02	
CENPV	centromere protein V	Q9CXS4	--	0,428		1,59E-01	
NPC2	NPC intracellular cholesterol transporter 2	Q9Z0J0	--	0,435		3,70E-04	
CD81	CD81 molecule	P35762	--	0,435		4,63E-04	
TMEM19	transmembrane protein 19	Q91W52	--	0,436		4,76E-02	
ROCK1	Rho associated coiled-coil containing protein kinase 1	P70335	--	0,441		1,46E-01	
DPY19L1	dpy-19 like 1	A6X919	--	0,449		2,57E-02	
ICAM1	intercellular adhesion molecule 1	P13597	--	0,45		1,32E-02	
LY6E	lymphocyte antigen 6 family member E	Q64253	--	0,452		1,34E-02	
CSF1	colony stimulating factor 1	P07141	P07141	0,472	0,517	2,00E-01	2,00E-01
HRAS	HRas proto-oncogene, GTPase	Q61411	Q61411	0,472	-0,118	1,41E-01	4,30E-01
**NDUFB10**	**NADH:ubiquinone oxidoreductase subunit B10**	Q9DCS9	--	0,475		8,04E-02	
WWC2	WW and C2 domain containing 2	Q6NXJ0	--	0,491		2,11E-02	
**Plpp1**	**phospholipid phosphatase 1**	Q61469	--	0,5		1,17E-01	
APOA1	apolipoprotein A1	Q00623	Q00623	0,502	0,453	3,69E-01	3,06E-02
**DNAJA3**	**DnaJ heat shock protein family (Hsp40) member A3**	Q99M87	--	0,508		4,12E-02	
ABAT	4-aminobutyrate aminotransferase	P61922	--	0,511		3,10E-02	
EPHX2	epoxide hydrolase 2	P34914	--	0,52		4,26E-02	
TMEM127	transmembrane protein 127	Q8BGP5	--	0,522		2,26E-01	
LRP1	LDL receptor related protein 1	Q91ZX7	Q91ZX7	0,522	0,105	6,03E-01	6,21E-02
UNC5B	unc-5 netrin receptor B	Q8K1S3	--	0,533		2,41E-02	
MIEN1	migration and invasion enhancer 1	Q9CQ86	--	0,544		4,32E-02	
**ACTG1**	**actin gamma 1**	P63260	--	0,55		1,79E-02	
A2M	alpha-2-macroglobulin	Q6GQT1	--	0,553		2,07E-01	
ATP13A3	ATPase 13A3	Q5XF89	Q5XF89	0,567	0,068	2,38E-02	2,26E-02
F3	coagulation factor III, tissue factor	P20352	P20352	0,577	0,278	9,01E-12	2,20E-03
FABP7	fatty acid binding protein 7	P51880	--	0,577		3,61E-02	
CHID1	chitinase domain containing 1	Q922Q9	--	0,588		2,71E-02	
HIGD2A	HIG1 hypoxia inducible domain family member 2A	Q9CQJ1	--	0,59		2,55E-02	
PRRT3	proline rich transmembrane protein 3	Q6PE13	--	0,593		4,65E-02	
LPCAT2	lysophosphatidylcholine acyltransferase 2	Q8BYI6	Q8BYI6	0,615	0,293	2,77E-03	1,35E-02
ITIH2	inter-alpha-trypsin inhibitor heavy chain 2	Q61703	--	0,619		7,31E-02	
STXBP1	syntaxin binding protein 1	O08599	--	0,65		1,74E-01	
ERC1	ELKS/RAB6-interacting/CAST family member 1	Q99MI1	--	0,675		1,34E-01	
**PLCD1**	**phospholipase C delta 1**	Q8R3B1	--	0,732		1,05E-02	

VTN	vitronectin	P29788	P29788	0,735	0,474	1,86E-01	2,60E-01
**MT-ND3**	**NADH dehydrogenase, subunit 3 (complex I)**	P03899	--	0,775		4,13E-02	
**SDHAF4**	**succinate dehydrogenase complex assembly factor 4**	Q8BTE0	--	0,807		2,54E-01	
TRMT61A	tRNA methyltransferase 61A	Q80XC2	--	0,817		1,35E-01	
CFB	complement factor B	P04186	--	0,826		3,32E-01	
RXFP1	relaxin/insulin like family peptide receptor 1	Q6R6I7	--	0,852		3,48E-01	
Krt10	keratin 10	P02535	P02535	0,871	0,85	1,13E-04	4,67E-04
FRMD4A	FERM domain containing 4A	Q8BIE6	--	0,882		1,48E-02	
**ACTG2**	**actin, gamma 2, smooth muscle, enteric**	P63268	--	0,923		6,00E-03	
**RLIM**	**ring finger protein, LIM domain interacting**	Q9WTV7	--	0,955		6,09E-01	
UPRT	uracil phosphoribosyltransferase homolog	B1AVZ0	--	0,975		3,21E-02	
KRT17	keratin 17	Q9QWL7	Q9QWL7	1,007	0,374	3,86E-03	2,05E-02
KRT16	keratin 16	Q9Z2K1	--	1,021		4,58E-02	
SFN	stratifin	O70456	--	1,12		3,37E-02	
KRT6B	keratin 6B	P50446	--	1,173		6,11E-06	
KRT5	keratin 5	Q922U2	--	1,176		1,12E-02	
ALB	albumin	P07724	--	1,302		3,63E-02	
ZFHX3	zinc finger homeobox 3	Q61329	--	1,332		1,29E-01	
GFAP	glial fibrillary acidic protein	P03995	--	1,491		1,45E-01	
Krt42	keratin 42	Q6IFX2	--	2,018		5,38E-03	
ATP1A2	ATPase Na+/K+ transporting subunit alpha 2	--	--				
ATP2A1	ATPase sarcoplasmic/endoplasmic reticulum Ca2+ transporting 1	--	--				
MAP1LC3	--	--	--				
MAP2K2	mitogen-activated protein kinase kinase 2	--	--				
YY1	YY1 transcription factor	--	--				
COMMD1	copper metabolism domain containing 1	--	Q8K4M5		0,679		4,91E-01
STON1	stonin 1	--	Q8CDJ8		0,53		4,68E-02
BLVRA	biliverdin reductase A	--	Q9CY64		0,424		3,22E-01
ITM2C	integral membrane protein 2C	--	Q91VK4		0,338		4,56E-01
GTF2H4	general transcription factor IIH subunit 4	--	O70422		0,314		4,44E-01
PUSL1	pseudouridylate synthase-like 1	--	A2ADA5		0,307		2,69E-02
SH3BP4	SH3 domain binding protein 4	--	Q921I6		0,3		2,79E-02
FKBP10	FK506 binding protein 10	--	Q61576		0,291		4,83E-02
ANXA11	annexin A11	--	P97384		0,271		4,65E-02
ZCCHC24	zinc finger CCHC-type containing 24	--	B2RVL6		0,239		3,26E-02

MLLT11	myeloid/lymphoid or mixed-lineage leukemia; translocated to, 11	--	P97783	0,233	4,74E-02
GGA1	golgi associated, gamma adaptin ear containing, ARF binding protein 1	--	Q8R0H9	0,209	4,52E-01
KDM1A	lysine demethylase 1A	--	Q6ZQ88	0,209	4,27E-01
NOTCH2	notch 2	--	O35516	0,204	5,91E-01
HGSNAT	heparan-alpha-glucosaminide N-acetyltransferase	--	Q3UDW8	0,17	2,38E-02
PSMB4	proteasome subunit beta 4	--	P99026	0,17	3,21E-02
RTN1	reticulon 1	--	Q8K0T0	0,154	5,25E-01
**ACAD8**	**acyl-CoA dehydrogenase family member 8**	--	Q9D7B6	0,15	1,82E-02
PIP4K2B	phosphatidylinositol-5-phosphate 4-kinase type 2 beta	--	Q80XI4	0,131	3,39E-02
S100A10	S100 calcium binding protein A10	--	P08207	0,125	5,26E-03
SMTN	smoothelin	--	Q921U8	0,121	1,77E-02
**COX5A**	**cytochrome c oxidase subunit 5A**	--	P12787	0,119	4,73E-02
SOAT1	sterol O-acyltransferase 1	--	Q61263	0,119	5,43E-01
HARS2	histidyl-tRNA synthetase 2, mitochondrial	--	Q99KK9	0,115	4,90E-02
SH3KBP1	SH3 domain containing kinase binding protein 1	--	Q8R550	0,115	4,63E-02
EBP	emopamil binding protein (sterol isomerase)	--	P70245	0,114	4,84E-02
HOOK2	hook microtubule tethering protein 2	--	Q7TMK6	0,114	4,02E-02
VPS26A	VPS26, retromer complex component A	--	P40336	0,114	5,89E-01
PPP1R9B	protein phosphatase 1 regulatory subunit 9B	--	Q6R891	0,113	2,19E-01
LRRFIP1	LRR binding FLII interacting protein 1	--	Q3UZ39	0,108	2,51E-02
**TXNDC9**	**thioredoxin domain containing 9**	--	Q9CQ79	0,106	2,87E-03
WNK1	WNK lysine deficient protein kinase 1	--	P83741	0,102	8,05E-04
CSNK1E	casein kinase 1 epsilon	--	Q9JMK2	0,1	5,63E-01
SYAP1	synapse associated protein 1	--	Q9D5V6	0,1	7,74E-02
**HSPA4**	**heat shock protein family A (Hsp70) member 4**	--	Q61316	0,095	6,00E-01
Abcb1b	ATP-binding cassette, sub-family B (MDR/TAP), member 1B	--	P06795	0,092	3,76E-01
CLCN5	chloride voltage-gated channel 5	--	Q9WVD4	0,083	1,73E-02
ATXN2	ataxin 2	--	O70305	0,081	4,41E-01
PRUNE1	prune exopolyphosphatase 1	--	Q8BIW1	0,08	4,77E-01
SNX17	sorting nexin 17	--	Q8BVL3	0,075	4,61E-01

PDIA5	protein disulfide isomerase family A member 5	--	Q921X9	0,069	4,56E-02
RCN1	reticulocalbin 1	--	Q05186	0,058	1,48E-02
ITM2B	integral membrane protein 2B	--	O89051	0,056	3,65E-01
ATAD3A	ATPase family, AAA domain containing 3A	--	Q925I1	0,055	8,61E-03
FTSJ3	FtsJ homolog 3	--	Q9DBE9	0,045	4,91E-02
PICALM	phosphatidylinositol binding clathrin assembly protein	--	Q7M6Y3	0,045	4,94E-01
RCC2	regulator of chromosome condensation 2	--	Q8BK67	0,033	3,57E-02
MTHFD1L	methylenetetrahydrofolate dehydrogenase (NADP+ dependent) 1-like	--	Q3V3R1	0,03	1,18E-02
**TXNDC12**	**thioredoxin domain containing 12**	--	Q9CQU0	0,02	4,28E-01
PCBP2	poly(rC) binding protein 2	--	Q61990	0,017	4,68E-02
PSME3	proteasome activator subunit 3	--	P61290	0,017	3,09E-01
CRYAB	crystallin alpha B	--	P23927	0,014	4,94E-01
PFN1	profilin 1	--	P62962	0,011	4,11E-01
Macf1	microtubule-actin crosslinking factor 1	--	Q9QXZ0	0,008	2,31E-02
RER1	retention in endoplasmic reticulum sorting receptor 1	--	Q9CQU3	-0,008	2,56E-01
G3BP2	G3BP stress granule assembly factor 2	--	P97379	-0,011	3,70E-02
EPN3	epsin 3	--	Q91W69	-0,013	6,07E-01
RTN3	reticulon 3	--	Q9ES97	-0,023	4,52E-01
ROCK2	Rho associated coiled-coil containing protein kinase 2	--	P70336	-0,029	1,07E-01
**ARPC2**	**actin related protein 2/3 complex subunit 2**	--	Q9CVB6	-0,03	3,89E-02
BIN1	bridging integrator 1	--	O08539	-0,03	4,05E-01
HNRNPA2B	heterogeneous nuclear ribonucleoprotein A2/B1	--	O88569	-0,043	4,82E-01
CTSB	cathepsin B	--	P10605	-0,05	1,07E-01
SPTAN1	spectrin alpha, non-erythrocytic 1	--	P16546	-0,05	4,00E-02
GDI2	GDP dissociation inhibitor 2	--	Q61598	-0,068	4,41E-02
APOB	apolipoprotein B	--	E9Q414	-0,076	1,93E-01
PACSIN1	protein kinase C and casein kinase substrate in neurons 1	--	Q61644	-0,077	4,45E-02
AGTRAP	angiotensin II receptor associated protein	--	Q9WVK0	-0,087	4,47E-01
**TXNDC5**	**thioredoxin domain containing 5**	--	Q91W90	-0,097	1,76E-02
TMEM59	transmembrane protein 59	--	Q9QY73	-0,107	4,26E-02
FABP5	fatty acid binding protein 5	--	Q05816	-0,115	3,54E-02
SRRT	serrate, RNA effector molecule	--	Q99MR6	-0,116	2,83E-02
PA2G4	proliferation-associated 2G4	--	P50580	-0,12	1,49E-02
ARCN1	archain 1	--	Q5XJY5	-0,13	2,41E-01

**RPS11**	**ribosomal protein S11**	--	P62281	-0,136		3,09E-03
RAB2B	RAB2B, member RAS oncogene family	--	P59279	-0,145		4,38E-02
PRMT1	protein arginine methyltransferase 1	--	Q9JIF0	-0,149		4,58E-02
**RPL30**	**ribosomal protein L30**	--	P62889	-0,159		4,24E-02
CYR61	cysteine rich angiogenic inducer 61	--	P18406	-0,174		3,25E-01
GUSB	glucuronidase beta	--	P12265	-0,175		2,02E-02
Hnrnpa1	heterogeneous nuclear ribonucleoprotein A1	--	P49312	-0,185		1,89E-02
EMB	embigin	--	P21995	-0,2		1,02E-02
**RRP12**	**ribosomal RNA processing 12 homolog**	--	Q6P5B0	-0,212		2,98E-02
PABPC1	poly(A) binding protein cytoplasmic 1	--	P29341	-0,22		7,16E-03
SNX27	sorting nexin family member 27	--	Q3UHD6	-0,232		3,72E-01
DKC1	dyskerin pseudouridine synthase 1	--	Q9ESX5	-0,249		4,68E-02
DHCR24	24-dehydrocholesterol reductase	--	Q8VCH6	-0,253		1,35E-02
NAA25	N(alpha)-acetyltransferase 25, NatB auxiliary subunit	--	Q8BWZ3	-0,253		3,65E-02
PRKCD	protein kinase C delta	--	P28867	-0,255		6,00E-01
SMC4	structural maintenance of chromosomes 4	--	Q8CG47	-0,288		3,79E-02
ABRACL	ABRA C-terminal like	--	Q4KML4	-0,359		3,92E-02
FBN2	fibrillin 2	--	Q61555	-0,475		2,02E-04
THOC5	THO complex 5	--	Q8BKT7	-0,529		5,26E-01
SYNJ1	synaptojanin 1	--	Q8CHC4	-0,612		4,92E-01
Symbol	Entrez Gene Name	GenPept/UniPro	GenPept/UniP		Expr False Di	Expr False Discovery Rate (q-value)(A4)
